# Extended Safety, Immunogenicity and Efficacy of a Blood-Stage Malaria Vaccine in Malian Children: 24-Month Follow-Up of a Randomized, Double-Blinded Phase 2 Trial

**DOI:** 10.1371/journal.pone.0079323

**Published:** 2013-11-18

**Authors:** Matthew B. Laurens, Mahamadou A. Thera, Drissa Coulibaly, Amed Ouattara, Abdoulaye K. Kone, Ando B. Guindo, Karim Traore, Idrissa Traore, Bourema Kouriba, Dapa A. Diallo, Issa Diarra, Modibo Daou, Amagana Dolo, Youssouf Tolo, Mahamadou S. Sissoko, Amadou Niangaly, Mady Sissoko, Shannon Takala-Harrison, Kirsten E. Lyke, Yukun Wu, William C. Blackwelder, Olivier Godeaux, Johan Vekemans, Marie-Claude Dubois, W. Ripley Ballou, Joe Cohen, Tina Dube, Lorraine Soisson, Carter L. Diggs, Brent House, Jason W. Bennett, David E. Lanar, Sheetij Dutta, D. Gray Heppner, Christopher V. Plowe, Ogobara K. Doumbo

**Affiliations:** 1 Malaria Group, Howard Hughes Medical Institute/Center for Vaccine Development, University of Maryland School of Medicine, Baltimore, Maryland, United States of America; 2 Malaria Research and Training Center, University of Science, Techniques and Technology, Bamako, Mali; 3 GlaxoSmithKline Vaccines, Rixensart, Belgium; 4 EMMES Corporation, Rockville, Maryland, United States of America; 5 Malaria Vaccine Development Program, U.S. Agency for International Development, Washington, DC, United States of America; 6 U.S. Military Malaria Vaccine Program, Walter Reed Army Institute of Research, Silver Spring, Maryland, United States of America; Kenya Medical Research Institute (KEMRI), Kenya

## Abstract

**Background:**

The FMP2.1/AS02_A_ candidate malaria vaccine was tested in a Phase 2 study in Mali. Based on results from the first eight months of follow-up, the vaccine appeared well-tolerated and immunogenic. It had no significant efficacy based on the primary endpoint, clinical malaria, but marginal efficacy against clinical malaria in secondary analyses, and high allele-specific efficacy. Extended follow-up was conducted to evaluate extended safety, immunogenicity and efficacy.

**Methods:**

A randomized, double-blinded trial of safety, immunogenicity and efficacy of the candidate *Plasmodium falciparum* apical membrane antigen 1 (AMA1) vaccine FMP2.1/AS02_A_ was conducted in Bandiagara, Mali. Children aged 1–6 years were randomized in a 1∶1 ratio to receive FMP2.1/AS02_A_ or control rabies vaccine on days 0, 30 and 60. Using active and passive surveillance, clinical malaria and adverse events as well as antibodies against *P. falciparum* AMA1 were monitored for 24 months after the first vaccination, spanning two malaria seasons.

**Findings:**

400 children were enrolled. Serious adverse events occurred in nine participants in the FMP2.1/AS02_A_ group and three in the control group; none was considered related to study vaccination. After two years, anti-AMA1 immune responses remained significantly higher in the FMP2.1/AS02_A_ group than in the control group. For the entire 24-month follow-up period, vaccine efficacy was 7.6% (p = 0.51) against first clinical malaria episodes and 9.9% (p = 0.19) against all malaria episodes. For the final 16-month follow-up period, vaccine efficacy was 0.9% (p = 0.98) against all malaria episodes. Allele-specific efficacy seen in the first malaria season did not extend into the second season of follow-up.

**Interpretation:**

Allele-specific vaccine efficacy was not sustained in the second malaria season, despite continued high levels of anti-AMA1 antibodies. This study presents an opportunity to evaluate correlates of partial protection against clinical malaria that waned during the second malaria season.

**Trial Registration:**

Clinicaltrials.gov NCT00460525 NCT00460525

## Introduction

A highly efficacious malaria vaccine that would reduce morbidity and mortality worldwide and advance the eradication agenda continues to be a research priority [1]. The apical membrane antigen 1 (AMA1) candidate malaria vaccine FMP2.1/AS02_A_ was developed for potential use as a stand-alone vaccine and/or as a component of a multi-antigen vaccine to improve on the efficacy of RTS,S/AS01 [2]. FMP2.1 is recombinant AMA1 based on the 3D7 clone of *P. falciparum* that is produced in and purified from *Escherichia coli* [3]. The FMP2.1 protein was formulated with GlaxoSmithKline’s proprietary adjuvant, AS02_A_, due to the strong immunogenicity and favorable safety profile of this adjuvant system in infants and children [4]–[11], and with potential combination with other subunit antigens using similar adjuvant systems in mind [2], [12], [13].

AMA1 is an 83-kilodalton surface protein expressed by mature intra-erythrocytic malaria parasites that is processed to a 66-kilodalton protein before being exported to the merozoite surface around the time of rupture of the infected erythrocyte [14]. It is highly polymorphic [15], [16], allowing the parasite to evade antibody-mediated inhibition of parasite growth [17]. The extracellular domain of AMA1 is divided into three sub-domains based on the pattern of disulfide bonds. The preponderance of the polymorphism in AMA1 is located in Domain 1, which has 32 polymorphic amino acid positions. Domain 2 has 11 such polymorphic sites while 9 reside in Domain 3 [18]. The remaining polymorphic sites are in the pro-sequence region (9) and the cytosolic region (3). Domain 1 is divided into three clusters (clusters 1, 2 and 3) based on amino acid spatial proximity [19], and the most polymorphic region of AMA1 is a loop within cluster 1 (the cluster 1 loop, c1L) containing eight highly polymorphic codons that encode as many as six different amino acids at position 197, and four amino acids each at positions 200 and 201. Antigenic escape residue modeling [20], *in vitro* analysis [21] and molecular epidemiology studies [22] identified the polymorphic amino acids in c1L (residues 196, 197, 199, 200, 201, 204, 206 and 207) as being the main targets of naturally-acquired protective antibodies. It was therefore hypothesized that these amino acids could be important in determining allele-specific efficacy in clinical trials of AMA1-based malaria vaccines.

The current report follows previous studies of the FMP2.1/AS02_A_ candidate vaccine conducted at the study site. Phase 1 dose-escalation testing in semi-immune adults found that the vaccine was well-tolerated and highly immunogenic, with evidence of enhanced parasite growth inhibition activity in sera from high dose vaccinees compared to controls [23]. Phase 1 dose-escalation testing in children confirmed findings of tolerability and immunogenicity [24]. Results of the primary analysis of Phase 2 testing for the first eight months of follow-up, through the first malaria season after vaccination, were also reported [25]. No serious adverse events related to vaccination were detected and laboratory safety tests revealed no significant differences in out-of-range values between groups. Local reactions were similar to those reported in phase 1 testing, with increased injection site swelling reported in the AMA1 vaccine group. Anti-AMA1 antibody titers peaked at 30 days after the last vaccination and remained at high levels through study day 240 (the primary observation period). Efficacy against the primary end point was 17.4% (p = 0.18). Efficacy against first and multiple clinical episodes defined using different parasite density thresholds was approximately 20% with varying statistical significance. Efficacy against clinical malaria caused by parasites with AMA1 corresponding to the vaccine strain at the eight pre-defined polymorphic amino acid sites in c1L was 64.3% (p = 0.03). Detailed molecular analyses concluded that just one of these amino acid positions, at codon 197, was critical for allele-specific efficacy [26]. Cumulative parasite density in all asymptomatic and symptomatic infections measured as area-under-the-curve (AUC) was significantly lower in the AMA1 vaccine group. Results of evaluation of safety, immunogenicity and efficacy of the FMP2.1/AS02_A_ candidate vaccine over the entire 24-month follow-up period are reported here.

## Methods

### Ethics Statement

The study protocol was approved by institutional review boards of the University of Sciences, Techniques and Technology Faculty of Medicine, Pharmacy and Dentistry in Bamako, Mali; the University of Maryland, Baltimore; the Walter Reed Army Institute of Research; and the United States Army Surgeon General. Written informed consent was obtained before starting screening and enrollment. Verbal consent of illiterate parents or guardians was administered and then documented using their thumbprints, a process verified by signatures of independent witnesses. The trial was monitored by the National Institute of Allergy and Infectious Diseases and the United States Army Medical Material Development Activity. An independent data and safety monitoring board and a local safety monitor were appointed. The trial was conducted in compliance with the International Conference on Harmonization of Good Clinical Practices, the Declaration of Helsinki and regulatory requirements of Mali.

### Study Design and Participants

Details of the study design and enrollment including the study protocol and statistical analysis plan were published previously for this double-blinded, randomized, controlled Phase 2 clinical trial [25]. The CONSORT checklist for this trial is available as supporting information; see Checklist S1. Participants received either the FMP2.1/AS02_A_ vaccine or rabies vaccine (control). The study population included children aged 1–6 years at the time of enrollment (first vaccination) after informed consent was obtained from a parent or guardian. Participants were screened for acute and chronic illnesses by medical history, physical examination, laboratory testing (hematology, serology for Hepatitis B and C, renal function and hepatic function). Active and passive surveillance for malaria began at the first vaccination and continued throughout the follow-up period. Venous blood samples were obtained for safety analyses on the days of vaccination, one week after each vaccination, and one month after the last vaccination. Venous blood samples were obtained for immunogenicity analyses on the days of vaccination and 1, 3, 6, 10, 16 and 22 months after the last vaccination. Information on bed net use was obtained monthly for the first 7 months of follow-up, and at 12, 18 and at 24 months after randomization.

Participants were given either 50 µg of lyophilized FMP2.1 (Walter Reed Army Institute of Research, Silver Spring, USA) resuspended shortly before vaccination in 0.5 mL of AS02_A_ (GSK, Rixensart, Belgium) [23] or 1 mL of purified chick embryo rabies vaccine (RabAvert rabies vaccine, Chiron Vaccines). After the end of the study, treatment assignment was unblinded, and participants randomized to receive the FMP2.1/AS02_A_ vaccine were offered immunization with three doses of rabies vaccine.

### Randomization and Blinding

Participants were randomly assigned in a 1∶1 ratio to the FMP2.1/AS02_A_ or control vaccine groups according to a computer-generated predefined block randomization scheme by age stratum (1–2, 3–4 and 5–6 years). Study pharmacists were unblinded to treatment assignment at vaccination, and senior study investigators were unblinded after the primary study endpoint was reached (study day 240). Study staff at the field site, participants and guardians remained blinded to treatment assignment until the end of the study (study day 730). Vaccines were prepared in identical syringes and covered to conceal their contents. Study vaccinators were not involved in post-vaccination participant assessments of safety or reactogenicity.

## Procedures

Parents and guardians were asked to return to the project clinic on the grounds of the district hospital any time a participant became ill. Study physicians were available at all times for evaluation, treatment and documentation of all adverse events, including serious adverse events. The study paid for laboratory testing and medication for participants during the study follow-up period.

Clinical malaria was defined as fever (axillary temperature of 37.5°C or higher) with an asexual *P. falciparum* density of at least 2500 parasites/ µl. Severe malaria was recorded as a serious adverse event and was diagnosed based on WHO diagnostic criteria modified to include two additional criteria based on a previous study of severe malaria at the site [27]: inability to eat or drink, and protracted vomiting. Malaria case detection was both active and passive. At each scheduled study visit that included a blood draw, a thick blood smear was prepared and read in real time if the participant had any clinical symptom consistent with malaria, including fever. Smears from asymptomatic children were batched and read at a later time. Parents and guardians were instructed to bring ill participants to the study clinic where clinicians and laboratory staff were available at all times. Participants who presented with any symptom of malaria at unscheduled clinic visits had a thick blood smear read immediately for prompt treatment of acute malaria. The primary endpoint of vaccine efficacy against first clinical malaria episodes during the first eight months of follow-up was previously reported [25]. A secondary endpoint was to determine vaccine efficacy against the first clinical malaria episode and all clinical episodes using increasing parasitemia thresholds occurring during two years after randomization.

To assess immunogenicity, serum was collected at baseline and at study months 1, 2, 3, 5, 8, 12, 18, and 24 to measure anti-AMA1 antibody by a standard, optimized ELISA [3], [28]. Briefly, IgG ELISAs were performed using FMP2.1 as the capture antigen, in a serial two-fold dilution, and the titer was defined as the serum dilution required to yield an optical density of 1.0 in our assay. Results were reported in optical density units that were converted to units of µg/mL based on a standard curve.

Hemoglobin was measured at each vaccination, 7 days after each vaccination, and at study months 3, 4, 5, 6, 7, 8, 12, 18 and 24. Other hematologic and biochemical parameters were monitored up to 30 days after the last vaccination.

Parasite DNA extracted from dried blood filter paper samples was used to sequence the gene encoding *P. falciparum* AMA1. QiaAmp manufacturer’s instructions for the 96 DNA blood kit (Qiagen, Valencia CA) were followed to extract malaria parasite DNA. The entire 1861 base pair ectodomain coding sequence of the *ama1* gene was amplified using a previously described nested PCR [29]. Sequences were edited and aligned using 3D7 (Genbank number AF512508) as the reference sequence.

## Statistical Analysis

The study statistical analysis plan was agreed upon by the study sponsor, investigators and statistical consultant before analyses commenced. Statistical analyses of secondary safety and efficacy endpoints were done with SAS 9.2 statistical software (Cary, North Carolina, USA). The sample size was calculated based on the primary study endpoint–the time to first clinical malaria episode occurring between randomization and six months after the assigned date of the third vaccination.

Analyses of safety were done on data collected from all vaccinated children. The percentage of those with a serious adverse event, as classified by the MedDRA preferred-term level, reported from 0–24 months was tabulated. The frequency distributions of biochemical (serum creatinine and serum alanine aminotransferase) and hematological (hemoglobin, white blood cells, platelets) laboratory values beyond predefined reference ranges were compared using predefined severity grades. Analysis of immunogenicity was conducted on all samples available from participants according to their treatment assignment. Anti-AMA1 antibody titers were summarized by geometric mean titers with 95% confidence intervals.

The study was designed to have 90% power to detect 20% efficacy based on a 75% incidence of clinical malaria in the control group during the first eight months of follow-up. Primary analysis of vaccine efficacy was conducted in the intention-to-treat cohort (all randomized children, and data collected starting on the day of the first vaccination) for the first eight months after randomization. Analyses of vaccine efficacy were also calculated for the per-protocol cohort (all randomized children who received all 3 doses of the vaccine to which they were randomized and who completed at least 14 days of follow-up after the third vaccination). Predefined secondary analyses of vaccine efficacy included efficacy against all clinical episodes during the first eight months of follow-up, and efficacy against first clinical malaria episode and all clinical episodes using a range of parasitemia thresholds occurring in the 24 months of follow-up in the intention-to-treat cohort. Protocol-defined exploratory analyses included vaccine efficacy against malaria infection using asexual *P. falciparum* parasitemia; vaccine efficacy against episodes of clinical malaria due to parasites that had AMA1 genotypes identical to the 3D7 vaccine strain with respect to designated immunologically important AMA1 polymorphisms (codons 196, 197, 199, 200, 201, 204, 206, and 207) in the cluster 1 loop of domain I; vaccine efficacy against cumulative asexual *P. falciparum* parasite density measured for each child as the total AUC for parasitemia in both clinical malaria episodes and asymptomatic infections detected in monthly surveys; vaccine efficacy against anemia measured at active (study days 7, 30, 37, 60, 67, 90, 120, 150, 180, 210, 240, 364, 547, and 730) and passive follow-up timepoints; and vaccine efficacy against the incidence of severe malaria in the intention-to-treat cohort.

Efficacy estimates were obtained for the hazard ratio for first or only episode and for all episodes of *P. falciparum* clinical malaria. For analysis of first or only episodes, hazard ratios were estimated using a standard Cox regression model unadjusted for covariates unless otherwise indicated. Efficacy against multiple clinical episodes was assessed using Poisson regression, which was not adjusted for other covariates. Cumulative incidence of clinical malaria for 22 months after the third dose was estimated by the Kaplan Meier method to account for loss to follow-up. Fisher’s exact test was used to compare proportions. All p-values presented are two-sided.

Sequencher 4.8 software (Gene Codes Corporation, Michigan, USA) was used to align and edit parasite DNA sequences obtained from infections causing clinical malaria episodes. *Ama1* sequences were defined as collected from single/predominant infections or from multiple infections based on nucleotide base peak heights on the sequencing chromatogram. Multiple-allele infections were defined as those with a secondary base peak height of 50% or more of the primary peak height at any polymorphic site. Haplotypes were defined only for sequences determined to correspond to single/predominant infections. Polymorphic codons in c1L were used to define haplotypes for exploratory measures of extended allele-specific efficacy [20]. MEGA 4.50.3 [30] and DNASP 4.50.3 [31] software were used to estimate amino acid differences between the 3D7 vaccine-strain sequence and all single/predominant sequences.

To assess the extended strain-specific efficacy of FMP2.1/AS02_A_, we used SAS 9.2 statistical software (Cary, North Carolina, USA) to assess the time to the first clinical malaria episode with a vaccine-type 3D7 (DERHFDKY) and non-3D7 haplotypes of AMA1 c1L. To evaluate the cross-protective efficacy of the malaria vaccine, we modeled the time to first clinical malaria episode with alleles matching the most frequently observed non-vaccine-type AMA1 c1L haplotypes during the second malaria transmission season (day 241 to 730): Fab9 (DQRHFDKY), Dd2 (DRRLLDED), M5 (NGRDLNEY) and FVO (NGRDFNEY). A Cox proportional hazards regression model was used to assess the association between treatment arms and risk of a clinical episode with parasites having an AMA1 c1L haplotype identical to 3D7 or any of the most common non-vaccine alleles, while a χ^2^ test was used to compare the frequency of c1L haplotypes in the two treatment arms.

## Results

**Figure 1:**
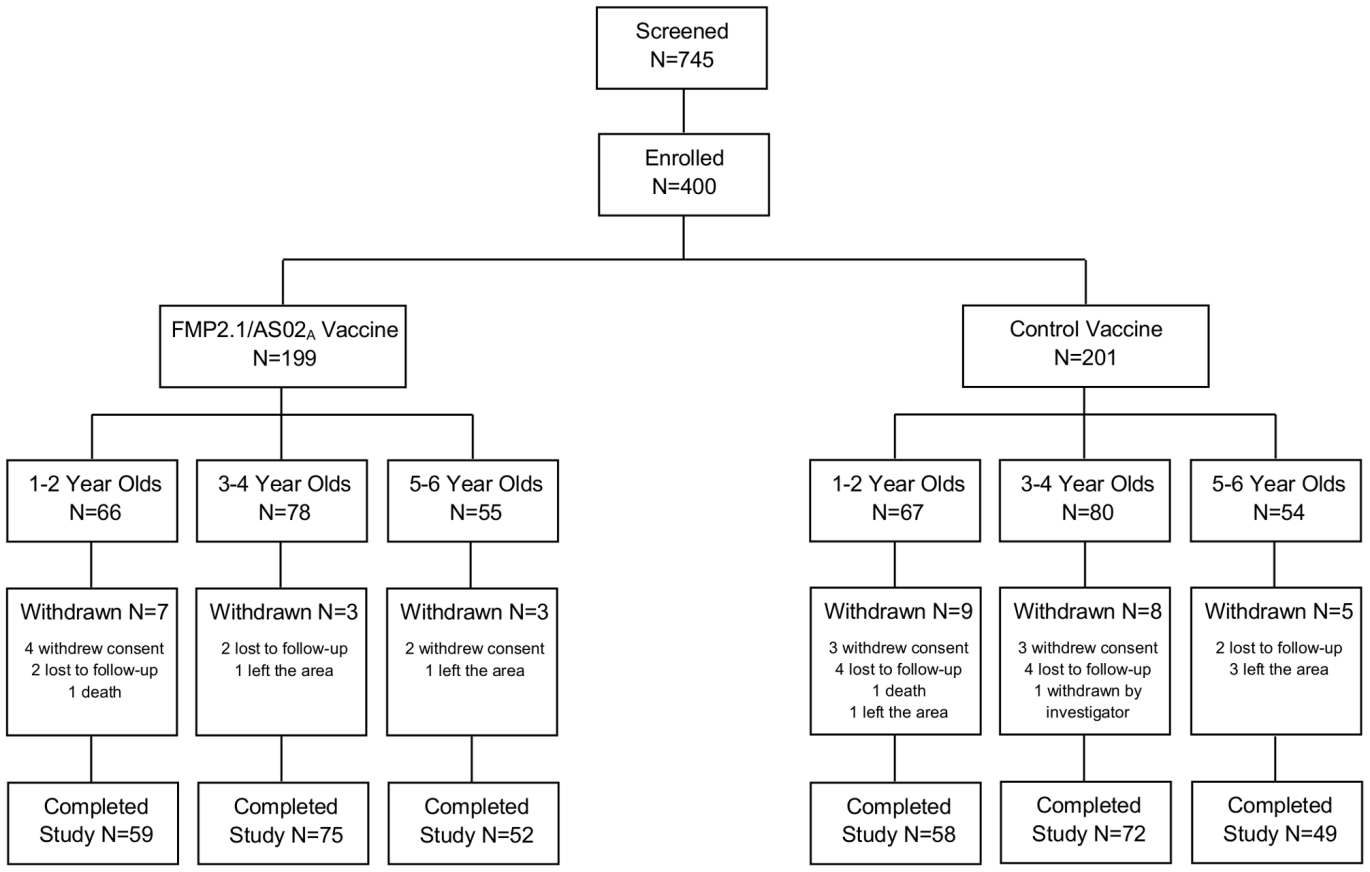
Disposition of study participants.

Four hundred children were enrolled from May 28 to July 4, 2007 at the Bandiagara Malaria Project research clinic on the grounds of the Bandiagara District Hospital in Bandiagara, Mali. All received at least one vaccination (Figure 1). The main reason for withdrawal from the study was loss to follow-up. Participants in both groups were balanced in terms of age and sex at enrollment. Mean age was 3.4 years (standard deviation 1.5), and 46% of participants were male. Insecticide treated bed net use was 31% at baseline in both groups, and was 62% in the FMP2.1/AS02_A_ group and 58% in the control group at the end of follow-up.

From study months 0–24, the number of children with at least one serious adverse event was greater in the FMP2.1/AS02_A_ compared to the control group (Table 1), but this difference was not statistically significant at the conventional level of 0.05. These serious adverse events largely reflected hospitalization rates in each group, as any adverse event requiring hospitalization was deemed serious. No participant experienced more than one serious adverse event. Three serious adverse events were due to malaria illness: two episodes of severe malaria and one episode of cerebral malaria, all occurring in the FMP2.1/AS02_A_ group at least four months after the last vaccination. One child from each group died during the study: one in the FMP2.1/AS02_A_ group from cerebral malaria 434 days after the last vaccination; and one in the control group as a result of status epilepticus, 256 days after the last vaccination. No serious adverse event was deemed related to vaccination and none occurred with a higher incidence in the malaria vaccine group compared to the control group that was of clinical concern to investigators or monitors.

**Table 1:** Serious adverse events experienced by all subjects (months 0–24, all randomized participants).

Description	FMP2.1/AS02A group (n = 199)	Control group (n = 201)	Outcome
Paralytic ileus	1 (0.5% [0.0–3.1])	0 (0.0% [0.0–2.3])	Resolved
Pyrexia	1 (0.5% [0.0–3.1])	0 (0.0% [0.0–2.3])	Resolved
Severe malaria	2 (1.0% [0.0–3.8])	0 (0.0% [0.0–2.3])	Resolved
Cerebral malaria	1 (0.5% [0.0–3.1])	0 (0.0% [0.0–2.3])	Death
Dehydration	1 (0.5% [0.0–3.1])	0 (0.0% [0.0–2.3])	Resolved
Febrile convulsion	1 (0.5% [0.0–3.1])	1 (0.5% [0.0–3.1])	Resolved
Status epilepticus	0 (0.0% [0.0–2.3])	1 (0.5% [0.0–3.1])	Death
Respiratory distress	1 (0.5% [0.0–3.1])	0 (0.0% [0.0–2.3])	Resolved
TOTAL	8 (4.0% [1.9–7.9])	2 (1.0% [0.0–3.8])	

Data are reported as: Number of participants with a serious adverse event (% [95% confidence interval]) among participants given at least one dose of vaccine.

No cases of severe anemia occurred during the study follow-up period. Anemia experienced in each group by severity is shown in Table 2. In the FMP2.1/AS02_A_ group, 40 episodes of anemia were recorded compared to 46 in the control group (p = 0.60). The incidence of Grade 2 anemia was higher in the control group (p = 0.02).

**Table 2:** Anemia experienced by all subjects (months 0–24, all randomized participants).

Description	FMP2.1/AS02_A_ group (n = 199)	Control group (n = 201)	p-value (Fisher’s exact)
Grade 1 (7.5–8.3 mg/dL)	34 (10.3 [7.1–14.4])	31 (9.3 [6.3–13.3])	0.80
Grade 2 (6.1–7.4 mg/dL)	4 (1.2 [0.3–3.1])	15 (4.5 [2.5–7.5])	0.02*
Grade 3 (5.0–6.0 mg/dL)	2 (6.0 [0.7–21.8])	0 (0.0 [0.0–11.1)])	0.50
TOTAL	40 (12.1 [9]–[16])	46 (13.9 [10]–[18])	0.60

Data are reported as incidence of participants with at least one episode of anemia per 100 person-years at risk (PYAR) with 95% confidence intervals (incidence per PYAR [95% confidence interval]) among participants given at least one dose of vaccine. Based on 331.2 PYAR for FMP2.1/AS02_A_ group and 331.9 PYAR for control group. *P<0.05.

**Figure 2:**
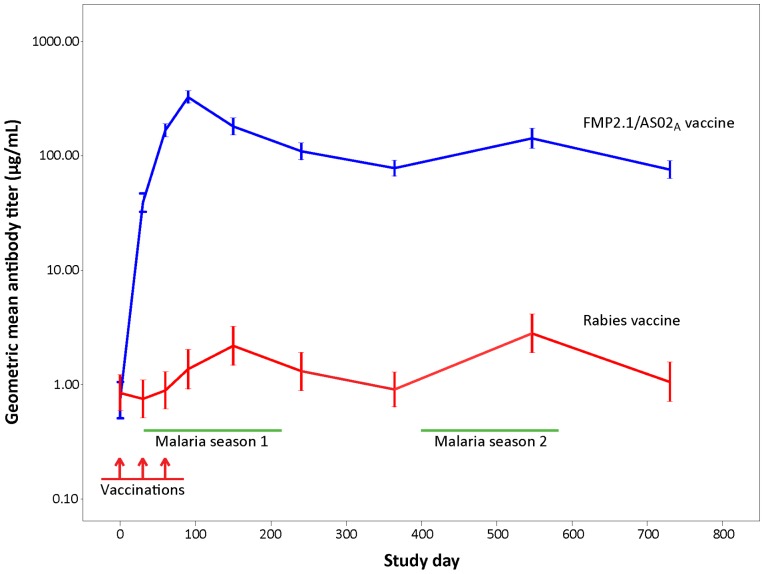
Levels of anti-apical membrane antigen 1 (AMA1) antibody to homologous recombinant AMA1 for FMP2.1/AS02_A_ vaccine and rabies vaccine recipients during the entire follow-up period (study days 0–730). Error bars indicate 95% confidence intervals.

Baseline anti-AMA1 antibody titers were similar in both groups [25]. By day 90, mean anti-AMA1 antibody titer in the FMP2.1/AS02_A_ group increased to levels similar to those measured in semi-immune adults at the site [23], with 93.3% experiencing an eight-fold or greater increase in antibody titer compared to baseline versus 16.2% in the control group. Anti-AMA1 antibodies remained at this high level for the remainder of the follow-up period in the FMP2.1/AS02_A_ group with 84.4% and 83.2% demonstrating an eight-fold increase at months 12 and 24 compared to 7.6% and 11.9% in the control group. Control group participants showed smaller increases in anti-AMA1 antibody levels, corresponding to peaks of natural exposure during the malaria transmission season (Figure 2).

**Figure 3:**
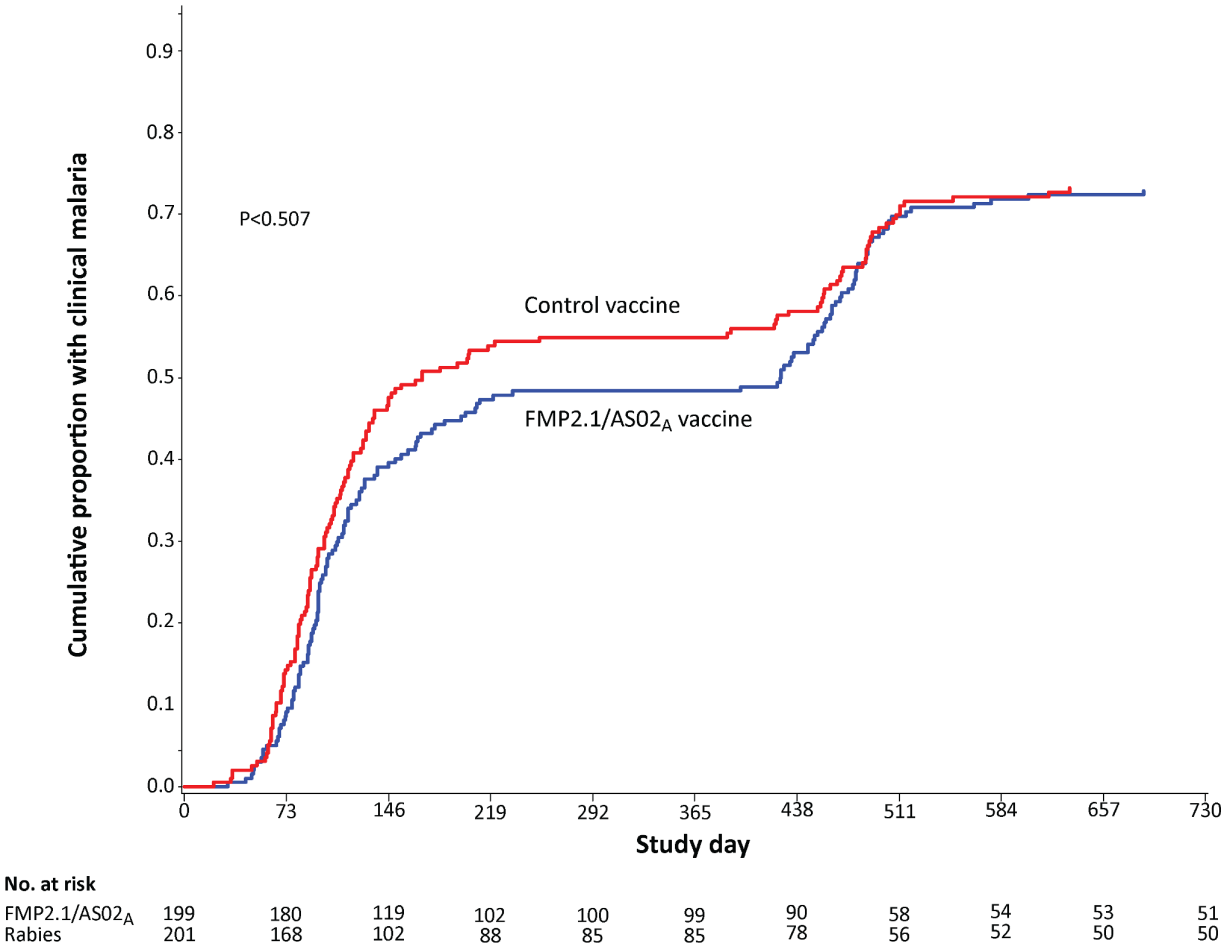
Cumulative incidence of first clinical malaria episode by treatment group during the entire follow-up period (study days 0–730), intention-to-treat.

During the entire follow-up period of 730 days in the intention-to-treat analysis, 142 FMP2.1/AS02_A_ vaccinees and 141 control vaccinees experienced at least one clinical episode of malaria. Clinical malaria incidence was 0.77 per person-year at risk (PYAR) in the FMP2.1/AS02_A_ group compared to 0.85 per PYAR in the control group. Vaccine efficacy against malaria in the FMP2.1/AS02_A_ group for months 0–24 was 7.6% (p = 0.51) for first or only malaria episodes and 9.9% (p = 0.19) for all malaria episodes (Table 3). The cumulative proportion of participants with at least one episode of *P. falciparum* malaria during the entire 730 days of follow-up was equivalent (Figure 3). During the period of follow-up from day 241–730, participants in the FMP2.1/AS02_A_ group experienced 178 total episodes of clinical malaria (0.68/PYAR) compared to 175 episodes in the control group (0.69/PYAR). Vaccine efficacy against all clinical episodes was 0.9% (95% CI: -22.2 to 19.5). Vaccine efficacy analyses are reported for the per-protocol dataset in the Supporting Information (Table S1).

**Table 3:** Vaccine efficacy against *P. falciparum* malaria, intention-to-treat cohort.

	FMP2.1/AS02A				Control				Vaccine Efficacy	
	n	Events	PYAR	Rate†	n	Events	PYAR	Rate†	% (95% CI)	p-value
**Months 0–24**										
First or only malariaepisode	199	142	196.4	0.72	201	141	178.7	0.79	7.6% (−16.7, 26.8%)*	0.51
All malaria episodes	199	299	388.5	0.77	201	325	380.5	0.85	9.9% (−5.4, 23.0%)‡	0.19
**Months 0–8**										
First malaria episode	199	95	94.1	1.01	201	106	87.3	1.21	17.6% (−8.7, 37.5%)*	0.17
All malaria episodes	199	121	127.2	0.95	201	150	125.8	1.19	20.2% (−1.4, 37.2%)‡	0.06
**Months 9–24**										
All malaria episodes	186	178	261.3	0.68	191	175	254.7	0.69	ND	ND

PYAR, Person Years At Risk. CI, confidence interval. ND, not done.

* Efficacy was calculated as 1– hazard ratio obtained using Cox proportional hazards modeling.

† Rate of malaria episodes per person-year at risk.

‡ Efficacy was calculated as 1– risk ratio obtained using Poisson regression.

**Figure 4:**
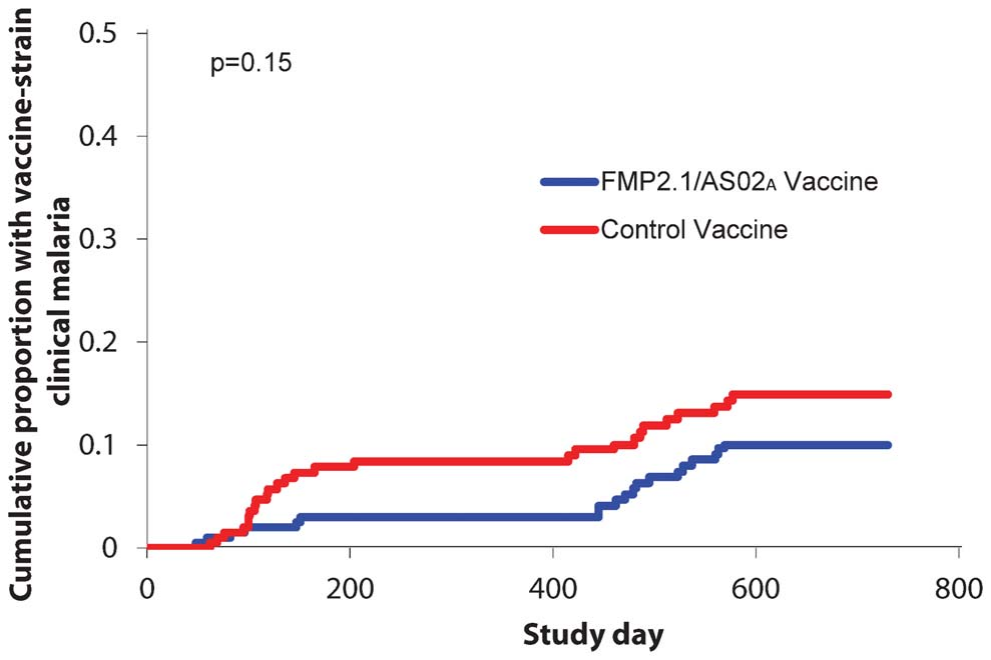
Vaccine efficacy against first episode of clinical malaria with apical membrane antigen 1 (AMA1) cluster 1 loop (c1L) identical to the vaccine strain 3D7 during the entire follow-up period (study days 0–730), intention-to-treat.

*Ama1* sequences from clinical malaria episodes occurring from study day 241 to 730 of follow-up were analyzed to assess the duration of allele-specific efficacy–the ability of FMP2.1/AS02_A_ to provide protection against clinical malaria with vaccine-type AMA1 c1L alleles. We observed 42 clinical episodes with *ama1* c1L sequences identical to 3D7 during the day 241–730 follow-up period. Twenty-two (52%) of these episodes were observed in the malaria vaccine group, and twenty (48%) in the control group. Seven of the 42 clinical episodes observed during the second malaria season were clinical episodes with *ama1* c1L sequences identical to 3D7 for participants who previously had clinical malaria with a 3D7 AMA1 c1L allele during the initial 240 days of follow-up, with three episodes diagnosed in malaria vaccine participants and four in control participants. The cumulative proportion of participants with at least one clinical episode due to an infecting 3D7 AMA1 c1L allele strain was comparable between the two treatment arms (p = 0.81) during the second period of follow-up. The overall allele-specific efficacy against first episodes of clinical malaria with vaccine-type AMA1 c1L alleles from the entire follow-up period from days 0–730 was 35% (95% C.I. -17%–64%, Table 4 and Figure 4).

**Table 4:** Allele-specific vaccine efficacy against *P. falciparum* malaria, intention-to-treat cohort.

	FMP2.1/AS02_A_				Control				Allele-Specific Efficacy	
	n	Events	PYAR†	Rate†	N	Events	PYAR	Rate†	% (95% CI)	p-value
**Months 0–24**										
First or only malariaepisode	199	19	371.0	0.05	201	25	363.7	0.07	35% (−17, 64%)*	0.15
All malaria episodes	199	28	388.5	0.07	201	36	380.5	0.09	24% (−12, 50%)†	0.17
**Months 0–8**										
First or only malariaepisode	199	6	128.5	0.05	201	16	126.7	0.13	64% (8, 86%)*	0.03
All malaria episodes	199	6	127.2	0.05	201	16	125.8	0.13	64% (8, 86%)†	0.03
**Months 9–24**										
First or only malariaepisode	186	13	182.4	0.07	175	9	172.7	0.05	ND	ND
All malaria episodes	186	22	261.3	0.08	175	20	254.7	0.08	ND	ND

PYAR, Person Years At Risk. CI, confidence interval. ND, not done.

* Efficacy was calculated as 1– hazard ratio obtained using Cox proportional hazards modeling.

† Rate of malaria episodes per person-year at risk.

‡ Efficacy was calculated as 1– risk ratio obtained using Poisson regression.

Analysis of vaccine efficacy against cumulative asexual parasite density measured as the total AUC did not show a protective effect in the FMP2.1/AS02_A_ group during the extended efficacy follow-up period from study days 241–730 for both the per-protocol and intention-to-treat datasets.

Although not part of the statistical analysis plan and not intended to estimate vaccine efficacy, to explore the possibility of a “rebound” increase in risk of malaria in the second season, we estimated the time without clinical malaria due to a 3D7 AMA1 c1L allele by setting the starting time to day 241, corresponding to the end of the first transmission season after vaccination. While the FMP2.1/AS02_A_ vaccine and control groups were no longer similar at this modified baseline due to the allele-specific efficacy demonstrated up to that time point, we felt it important to conduct this post-hoc analysis to examine whether there was any of increased risk of malaria after a period of lower risk as was previously documented in a study of short-term antimalarial drug prophylaxis at the study site [32]. There was no difference in the time to first clinical malaria episode with a 3D7 AMA1 c1L allele between the FMP2.1/AS02_A_ vaccine and the control vaccine groups (p = 0.40) (data not shown). The estimated vaccine efficacy against clinical malaria episodes showed no difference between FMP2.1/AS02_A_ and control vaccine (hazard ratio 1.43, 95% CI: 0.61–3.35). Moreover, the incidence of clinical malaria with 3D7 AMA1 c1L was similarly distributed between the FMP2.1/AS02_A_ and control vaccine groups (p = 0.81). Finally, when we compared the incidence of 3D7 AMA1 c1L type clinical episodes observed during the first 240 days in all participants to 3D7 c1L alleles identified during days 241–270 of follow-up in all participants, the frequencies were similar (p = 0.63), signifying a stable incidence of 3D7 AMA1 c1L type infections during the entire follow-up period. These results indicate that the allele-specific efficacy of the malaria vaccine did not extend into the second malaria season and that the incidence of 3D7 AMA1 c1L allele was stable during the two transmission seasons.

## Discussion

During the first 240 days of follow-up, vaccination of malaria-exposed Malian children aged 1–6 years with FMP2.1/AS02_A_ according to a 0, 1, 2-month schedule did not show result in significant efficacy against the primary endpoint, but secondary analyses showed approximately 20% efficacy against first and multiple clinical malaria episodes defined using different parasite density thresholds. The vaccine had 64.3% efficacy (p = 0.03) against clinical malaria caused by parasites with AMA1 corresponding to the vaccine strain at pre-defined polymorphic amino acids sites. The safety profile was favorable based on comparison of reactogenicity, laboratory values and adverse event rates in FMP2.1/AS02_A_ vaccinees compared to controls. High anti-AMA1 antibody titers were induced and sustained during follow-up, and cumulative parasitemia as measured by AUC was reduced in FMP2.1/AS02_A_ vaccinees compared to controls [25].

During the entire follow-up period from study days 0–730, the FMP2.1/AS02_A_ vaccine did not demonstrate allele-specific efficacy as seen during the first malaria season. The vaccine likewise provided no efficacy against first clinical malaria episodes, all malaria episodes, or episodes due to parasites matching the vaccine strain in the extended efficacy surveillance period for our study population. The rate of Grade 2 anemia during the entire follow-up period was slightly higher in the control group, although this event was uncommon in the study population (15 versus 4 episodes, p = 0.02). Although more serious adverse events occurred in the FMP2.1/AS02_A_ group compared to controls, this difference was not statistically significant and none of the severe events was deemed related to vaccination. Overall, the extended follow-up showed favorable safety data, with no concerning findings.

No evidence of a “rebound”–a period of increased risk of malaria following a period of protection by vaccines, drugs, nets or other interventions [33]–was observed in the FMP2.1/AS02_A_ group during months 9–24 of follow-up. Neither the overall incidence of clinical malaria nor incidence of clinical malaria due to the vaccine strain was increased in FMP2.1/AS02_A_ vaccinees during this interval after the initial efficacy follow-up period. This is reassuring as a previous study at the site found that after an initial period of protection, those given a single dose of sulfadoxine-pyrimethamine at the start of the malaria transmission season experienced delayed but then subsequently increased incidence of clinical malaria compared to controls who did not receive sulfadoxine-pyrimethamine [32].

Severe malaria occurred in three participants, all in the FMP2.1/AS02_A_ group. This low number of cases is likely related to early diagnosis and treatment of uncomplicated malaria in this study population with close active and passive surveillance that included continuous access to and frequent contact with study physicians and malaria diagnostics.

The FMP2.1/AS02_A_ vaccine induced high levels of anti-AMA1 antibody that peaked at study day 90 and persisted at high levels throughout the entire 24 months of follow-up. The increase in antibody level from baseline to the measurement just before the first episode of clinical malaria correlated with protection in the first eight months of the study. The results of the extended follow-up found no evidence of overall or strain-specific vaccine efficacy in the second malaria season of follow-up. The waning of strain-specific efficacy occurred despite a sustained high level of anti-AMA1 antibody in FMP2.1/AS02_A_ vaccinees, but this level may have been insufficient to protect against clinical disease in the second malaria season. As no efficacy was observed in the second malaria season, change in anti-AMA1 antibody level cannot be correlated with extended protection, but it may be possible to identify a marker of strain-specific protective immune response that was not measured and that did not persist through the second malaria season. Planned analyses of antibody avidity and subclass, cell-mediated immunity and growth inhibition analysis may help to better understand the strain-specific protection observed in the first malaria season following vaccination and provide insights into how to develop more broadly efficacious next generation malaria vaccines.

## Supporting Information

Table S1 **Vaccine efficacy against** ***P. falciparum*** **malaria, per-protocol cohort.**
(DOCX)

Checklist S1 **CONSORT checklist.**
(DOC)

Protocol S1 **Trial Protocol.**
(PDF)
