# Supplementary material for: Extended Safety, Immunogenicity and Efficacy of a Blood-Stage Malaria Vaccine in Malian Children: 24-Month Follow-Up of a Randomized, Double-Blinded Phase 2 Trial
Source: PLoS One. 2013 Nov 18;8(11):e79323. doi: 10.1371/journal.pone.0079323 (PMC3832522; doi:10.1371/journal.pone.0079323)
Supplement: Table S1 — Vaccine efficacy against P. falciparum malaria, per-protocol cohort. (DOCX) [file pone.0079323.s001.docx]

**Table S1. Vaccine efficacy against *P. falciparum* malaria, per-protocol cohort**

|  | **FMP2.1/AS02_A_** | | | |  | **Control** | | | |  | | **Vaccine Efficacy** | | | |  |
| --- | --- | --- | --- | --- | --- | --- | --- | --- | --- | --- | --- | --- | --- | --- | --- | --- |
|  | **n** | **Events** | **PYAR** | **Rate**† |  | **n** | **Events** | **PYAR** | **Rate**† | |  | | **% (95% CI)** | **p-value** | | |
| **Months 2-24** | | | | | | | | | | | | | | | | |
| First or only malaria episode | 186 | 132 | 156.9 | 0.84 |  | 191 | 130 | 156.6 | 0.83 | |  | | -2.5% (-30.6, 19.6%)* | 0.84 | | |
| All malaria episodes | 186 | 265 | 331.2 | 0.80 |  | 191 | 285 | 331.9 | 0.86 | |  | | 6.8% (-10.1, 21.1%)‡ | 0.19 | | |
| **Months 2-8** | | | | | | | | | | | | | | | | |
| First or only malaria episode | 186 | 84 | 55.9 | 1.50 |  | 191 | 90 | 54.6 | 1.65 | |  | | 7.5% (-24.5, 31.3%)* | | 0.17 | |
| All malaria episodes | 186 | 99 | 82.9 | 1.19 |  | 191 | 117 | 84.1 | 1.39 | |  | | 24.3% (-12.1, 34.3%)‡ | | 0.06 | |
| **Months 9-24** | | | | | | | | | | | | | | | | |
| All malaria episodes | 186 | 166 | 248.3 | 0.67 |  | 191 | 168 | 254.7 | 0.66 | |  | | - | | - | |

PYAR = Person Years At Risk

* Efficacy was calculated as 1 – hazard ratio obtained using Cox proportional hazards modeling

† Rate of malaria episodes per person-year at risk

‡ Efficacy was calculated as 1 – risk ratio obtained using Poisson regression

CI = confidence interval
