# Supplementary material for: Extended Safety, Immunogenicity and Efficacy of a Blood-Stage Malaria Vaccine in Malian Children: 24-Month Follow-Up of a Randomized, Double-Blinded Phase 2 Trial
Source: PLoS One. 2013 Nov 18;8(11):e79323. doi: 10.1371/journal.pone.0079323 (PMC3832522; doi:10.1371/journal.pone.0079323)
Supplement: Protocol S1 — Trial Protocol. (PDF) [file pone.0079323.s003.pdf]

# RANDOMIZED, CONTROLLED PHASE 2 CLINICAL TRIAL TO EVALUATE THE SAFETY, IMMUNOGENICITY AND EFFICACY OF THE AMA-1 MALARIA VACCINE FMP2.1/AS02A VS. RABIES VACCINE IN 1-6 YEAR OLD CHILDREN IN BANDIAGARA, MALI

**DMID Protocol Number:** 07-0003

**MMVDU Protocol Number:** MV-07

**UMB IRB Protocol Number:** H-28884

**HSRRB Protocol Number:** A-14262

**IND Number:** 11140

**GSK Biologicals Protocol:** Malaria-056 (110060)

**DMID Funding Mechanism:** NIAID U19AI065683

**Pharmaceutical Support Provided by:** GlaxoSmithKline Biologicals

**IND Sponsor:** Office of the Surgeon General, US Army

**Principal Investigator:** Mahamadou A. Thera MD MPH

**DMID Protocol Champion:** Abdollah Naficy MD

**DMID Medical Monitor:** Mirjana Nesin MD

**DMID Clinical Affairs Specialist:** Josephine Vinson MS

**DMID Regulatory Affairs Specialist:** Blossom Smith

**Draft or Version Number:** 1.0

23 March 2007

# STATEMENT OF COMPLIANCE

The study described in this protocol will be conducted according to current Good Clinical Practices (US 21 CFR Part 50-Protection of Human Subjects and Part 56-Institutional Review Boards, US 45 CFR 46, 21 CFR 312, 32 CFR 219, 10 USC 980 and the applicable rules and regulations of Mali).

The University of Bamako Faculty of Medicine, Pharmacy and Odonto-stomatology IRB (FWA00001769), the University of Maryland IRB (FWA00007145), the Walter Reed Army Institute of Research IRB (FWA00000015) and the Division of Microbiology and Infectious Diseases (DMID), NIAID, NIH will review and approve the protocol prior to study start. Documentation of the approval by these bodies will be kept in the PI's study file and Sponsor's regulatory files.

## SIGNATURE PAGE

The signatures below constitute the approval of this protocol and the attachments, and provide the necessary assurances that this trial will be conducted according to all stipulations of the protocol, including all statements regarding confidentiality, and according to local legal and regulatory requirements and applicable US federal regulations and ICH guidelines.

Research Center Director:

Signed: ______________________________ Date: ______________

Ogobara K Doumbo MD PhD
Professor

Site Principal Investigator:

Signed: ______________________________ Date: ______________

Mahamadou A. Thera MD MPH
Associate Professor

Co-Principal Investigator:

Signed: ______________________________ Date: ______________

Christopher V. Plowe MD MPH
Professor

# TABLE OF CONTENTS

Page
Statement of Compliance....2
Signature Page....3
Table of Contents....4
Protocol Summary....10
1 Background Information and Scientific Rationale....20
1.1 Background information....20
Malaria parasite life cycle....20
1.2 Disease burden....20
Malaria in Mali....20
1.3 Rationale....21
The AMA-1 antigen....21
Genetic diversity in AMA-1....22
1.4 The FMP2.1/AS02A vaccine....22
Preclinical toxicity, safety and reactogenicity of FMP2.1/AS02A....23
Clinical experience with FMP2.1/AS02A....23
Phase 1 trial in US adults....24
Phase 1 trial in Malian adults....27
Phase 1 trial in Malian children....32
Summary of clinical experience with FMP2.1/AS02A....41
Justification of 0-, 1-, 2-month schedule....41
Justification of the vaccine dosing regimen and next steps for the vaccine....41
Comparator vaccine....42
Safety of RabAvert/Rabipur® rabies vaccine....43
1.5 Potential risks and benefits....44
Potential risks....44
Vaccination....44
Known potential benefits....44
Risks to study personnel....45
2 Objectives....46
2.1 Study objectives....46
Overall study objective....46
Primary specific objective....46
Secondary specific objectives....46
Exploratory objectives....46
2.2 Study outcome measures....47
Primary outcome measures....47
Secondary outcome measures....47
3 Study Design....49
3.1 Overview....49
4 Study Enrollment and Withdrawal....51
4.1 Site description....51

Availability of medical and preventive care ........ 52
Insecticide treated nets ........ 52
4.2 Subject inclusion criteria ........ 53
4.3 Subject exclusion criteria ........ 53
Rationale for using clinical assessment of immunosuppression ........ 54
4.4 Treatment assignment procedures ........ 55
Randomization procedures ........ 55
Masking procedures ........ 55
Reasons for withdrawal ........ 56
Handling of withdrawals ........ 57
Termination of study ........ 57
5 Study Intervention/Investigational Product ........ 58
5.1 Study product descriptions ........ 58
Acquisition ........ 58
Formulation and packaging ........ 58
Product storage and stability ........ 59
5.2 Dosage, preparation and administration of investigational product ........ 60
Administration of vaccines ........ 60
5.3 Accountability procedures for the investigational product ........ 60
5.4 Concomitant medications/treatments ........ 60
6 Study Schedule ........ 61
6.1 Screening ........ 61
6.2 Enrollment/baseline ........ 62
6.3 Vaccination process ........ 62
6.4 Follow-up ........ 62
7 Methods for measuring efficacy ........ 63
7.1 Follow up methods ........ 63
Passive case detection ........ 63
Active surveillance ........ 63
7.2 Case definitions ........ 64
Clinical malaria ........ 64
Anemia ........ 64
Severe malaria ........ 64
7.3 Primary efficacy endpoint ........ 65
7.4 Secondary efficacy endpoints ........ 65
7.5 Exploratory efficacy endpoints ........ 65
8 Study Procedures/Evaluations ........ 66
8.1 Standard operating procedures (SOPs) ........ 66
8.2 Daily study procedures ........ 67
Outline of study procedures ........ 72
Health care provision ........ 73
8.3 Final study visit ........ 74
8.4 Early termination visit ........ 74
8.5 Unscheduled visit ........ 75

9 Sample handling and analysis ........ 76
9.1 Overview of sample collection, handling, transport and shipping. ........ 76
9.2 Overview of collection time points ........ 76
Safety 76
Serology ........ 76
CMI 77
9.3 Laboratory Assays ........ 77
Hematology and biochemistry ........ 77
Serology ........ 77
Malaria smears ........ 78
Additional assays ........ 78
10 Assessment of Safety ........ 81
10.1 Specification of safety parameters ........ 81
10.2 Methods and timing for assessing, recording, and analyzing safety parameters ... 81
Adverse events ........ 81
Surveillance period for adverse events ........ 82
Recording adverse events ........ 82
Solicited adverse events ........ 82
Unsolicited adverse events ........ 83
10.3 Assessment of intensity of non-serious adverse events ........ 83
10.4 Assessment of causality ........ 85
10.5 Following-up of adverse events and assessment of outcome ........ 85
10.6 Clinical malaria ........ 86
10.7 Serious adverse events ........ 86
Definition of a serious adverse event ........ 86
Reporting serious adverse events ........ 87
10.8 Treatment of adverse events ........ 89
10.9 Halting rules ........ 89
10.10 Safety oversight ........ 90
Local Medical Monitor ........ 90
10.11 Data and safety monitoring board (DSMB) ........ 91
11 Clinical Monitoring ........ 93
11.1 Site monitoring plan ........ 93
12 Statistical Considerations ........ 94
12.1 Case definition: Clinical malaria episode ........ 94
12.2 Primary endpoints ........ 94
12.3 Secondary endpoints ........ 94
12.4 Exploratory endpoints ........ 95
12.5 Study cohorts/datasets to be evaluated ........ 95
12.6 Sample size considerations ........ 96
12.7 Factors that may impact malaria risk ........ 98
Age and gender ........ 98
Insecticide treated nets ........ 98
Residence ........ 98

Malaria treatment ........ 98
12.8 Final analysis plan ........ 99
Analysis of demographics ........ 99
Analysis of efficacy ........ 99
Analysis of allele-specific efficacy ........ 99
Analysis of immunogenicity ........ 100
Analysis of safety ........ 101
Analysis of CMI responses ........ 101
13 Source Documents and Access to Source Data/Documents ........ 103
14 Quality Control and Quality Assurance ........ 104
15 Ethics/Protection of Human Subjects ........ 105
15.1 Ethical standard ........ 105
15.2 Institutional review board ........ 105
15.3 Informed consent process ........ 106
Screening and study informed consent ........ 106
Screening recruitment radio announcement text ........ 108
Compensation ........ 109
15.4 Subject confidentiality ........ 109
15.5 Study discontinuation ........ 110
15.6 Future use of stored specimens ........ 110
15.7 Justification for conducting the study in children ........ 110
15.8 Justification for exclusion ........ 111
16 Data Handling and Record Keeping ........ 112
16.1 Data management responsibilities ........ 112
16.2 Data capture methods ........ 112
16.3 Types of data ........ 113
16.4 Study records retention ........ 113
16.5 Protocol deviations ........ 113
17 Obligations to the USAMRMC, Office of Research Protections (ORP), Human Research Protection Office (HRPO) and Walter Reed Army Institute of Research Human Use Review Committee ........ 114
18 Publication Policy ........ 116
19 Literature ........ 117
20 SupplementS/Appendices ........ 122

List of Abbreviations

| | |
|---|---|
| 3D7: | A laboratory clone of *Plasmodium falciparum* |
| AE: | Adverse event |
| ALT: | Alanine aminotransferase |
| AMA-1: | Apical Membrane Antigen-1 of *P. falciparum* |
| AST: | Aspartate aminotransferase |
| AS02A: | Adjuvant system 2 of GlaxoSmithKline Biologicals |
| BMP: | Bandiagara Malaria Project (clinical trial site) |
| CBER: | Center for Biologics Evaluation and Research |
| CBC: | Complete blood count |
| CFR: | Code of Federal Regulations |
| CMI: | Cell mediated immunity |
| CRF: | Case report form |
| CS gene: | Circumsporozoite gene of *P. falciparum* |
| CSP: | Circumsporozoite protein |
| CTL: | Cytotoxic T lymphocyte |
| CVD: | Center for Vaccine Development, University of Maryland Baltimore |
| DMID: | Division of Microbiology & Infectious Diseases, NIAID, NIH |
| DNA: | Deoxyribonucleic acid |
| DSMB: | Data and Safety Monitoring Board |
| EGF: | Epidermal growth factor |
| EPI | Expanded Programme on Immunization |
| FDA: | US Food and Drug Administration |
| FWA: | Federal-Wide Assurance |
| ELISA: | Enzyme linked immunosorbent assay |
| FMP1: | Falciparum Malaria Protein 1 (3D7 MSP1-42 vaccine candidate antigen) |
| FMP2.1: | Falciparum Malaria Protein 2.1 (3D7 AMA-1 vaccine candidate antigen) |
| FMPOS: | Faculty of Medicine, Pharmacy and Odonto-stomatology, University of Bamako, Mali |
| GCP: | Good clinical practice |
| GIA: | Growth inhibition assay |
| GSK: | GlaxoSmithKline Biologicals |
| GMT: | Geometric mean titer |
| HDCV: | Human Diploid Cell Vaccine (Rabies) |
| HSRRB: | US Army Medical Research and Materiel Command's Human Subjects Research Review Board |
| HURC | WRAIR Human Use Review Committee (IRB) |
| ICH: | International Conference on Harmonisation |
| ID: | Identification |
| IDES: | Internet Data Entry System (AdvantageEDC) |
| IEC: | Institutional Ethical Committee |
| IM: | Intramuscular |
| IND: | Investigational new drug |
| IRB: | Institutional Review Board (ethical review committee), for this study including the |

| | |
|---|---|
| | University of Bamako Faculty of Medicine IRB, the University of Maryland Baltimore IRB, and the US Army's Human Subjects Research Review Board |
| ITN | Insecticide treated nets |
| LMM | Local Medical Monitor |
| MPL: | Monophosphoryl lipid A |
| 3D-MPL | 3-deacylated monophosphoryl lipid A |
| MRTC: | Malaria Research and Training Center |
| MMVDU: | Mali Malaria Vaccine Development Unit |
| MSP-1: | Merozoite surface protein-1 of *P. falciparum* |
| MSP-2: | Merozoite surface protein-2 of *P. falciparum* |
| MVDB: | Malaria Vaccine Development Branch, NIAID, NIH |
| NIAID: | National Institute of Allergy and Infectious Diseases |
| NIH: | US National Institutes of Health |
| NANP: | Repeat epitopes of the circumsporozoite protein |
| PBMC: | Peripheral blood mononuclear cells |
| PI: | Principal Investigator |
| OCRA: | Office of Clinical Research Affairs, DMID, NIAID, NIH |
| QA: | Quality assurance |
| QC: | Quality control |
| QS21: | *Quillaja saponaria* 21 (saponin derivative) |
| RTS,S: | Fusion protein between circumsporozoite protein based antigen and Hepatitis B surface antigen and 226 amino acid polypeptide corresponding to the surface antigen of hepatitis B virus (adw serotype) |
| SBAS2: | SmithKline Beecham Adjuvant System 2 |
| SAE: | Serious adverse event |
| SME: | Sponsor's Medical Expert |
| SMC: | Safety Monitoring Committee |
| SNP | Single nucleotide polymorphism |
| SOP: | Standard Operating Procedure |
| UMB: | University of Maryland Baltimore |
| USAID: | US Agency for International Development |
| USAMMDA: | US Army Medical Materiel Development Activity |
| WHO: | World Health Organization |
| WRAIR: | Walter Reed Army Institute of Research |

# PROTOCOL SUMMARY

| | |
|---|---|
| **Title:** | Randomized, controlled, Phase 2 clinical trial to evaluate the efficacy, safety and immunogenicity of the AMA-1 malaria vaccine FMP2.1/AS02A vs. rabies vaccine in 1-6 year old children in Bandiagara, Mali |
| **Phase:** | 2 |
| **Population:** | 400 healthy children aged 1-6 years residing in Bandiagara, Mali |
| **Number of Sites:** | 1 |
| **Study Duration:** | 27 months |
| **Subject Participation Duration:** | 26 months (including screening and extended follow-up) |
| **Description of Agent or Intervention:** | Three doses administered a month apart of:<br>• 50 µg recombinant subunit protein FMP2.1 (*Plasmodium falciparum* Apical Membrane Antigen-1 from strain 3D7 expressed in and purified from E*scherichia coli*), adjuvanted with:<br>• 0.5 mL of GSK's AS02A (proprietary oil-in-water emulsion and phosphate buffered saline with the immunostimulants monophosphoral lipid A and QS21) |
| **Objectives:** | Primary:<br>• Determine the efficacy of FMP2.1/AS02A in children aged 1-6 years against first clinical malaria episodes (axillary temperature of $\geq$ 37.5 $^{o}$C and parasitemia of $\geq$2500/mm$^3$) occurring between randomization and six months after the assigned date of the third immunization<br>• Assess the safety of the vaccine in children aged 1-6 years<br>Secondary:<br>• To describe the dynamics of anti-AMA-1 antibody responses in recipients of the malaria vaccine compared to natural responses in the control group<br>• To determine whether serum anti-AMA-1 IgG titer by ELISA one month after the third immunization correlates with protection against clinical malaria episode |

| | |
|---|---|
| | • To measure allele-specific efficacy against parasites with AMA-1 genotypes homologous to and heterologous to the 3D7 clone of *P. falciparum*<br>• To determine vaccine efficacy against clinical malaria episodes occurring between randomization and six months after the assigned date of the third immunization<br>• If efficacy is observed based on the primary endpoint, to determine vaccine efficacy against first clinical malaria episode and all clinical episodes (using increasing parasitemia thresholds) occurring during two years after randomization<br><br>Exploratory:<br>• To determine vaccine efficacy of FMP2.1/AS02A in children aged 1-6 years against clinical malaria episodes using increasingly specific definitions of clinical episodes (parasitemia thresholds of any parasitemia, 100, 1000, 2500, 5000, 10,000, 20,000, 50,000 and 100,000/mm$^3$; and any symptoms consistent with malaria, with and without measured fever) occurring between randomization and six months after the assigned date of the third immunization<br>• To measure the age-specific vaccine efficacy in children aged 1-2, 3-4 and 5-6 years<br>• To determine whether three doses of vaccine significantly protect against malaria infection, as measured by the prevalence and density of parasitemia by comparing malaria and control vaccine recipients with regard to rates of asexual *P. falciparum* parasite density and gametocyte counts, as determined at active surveillance time points.<br>• To determine vaccine efficacy against the incidence of anemia<br>• To determine vaccine efficacy against the incidence of severe malaria<br>• To measure cellular immune responses to FMP2.1 at baseline and after immunization and assess the association of these responses with vaccine efficacy<br>• To determine the specificity of antibodies and cell mediated immune responses to diverse AMA-1 genotypes in addition to 3D7, by measuring by ELISA, growth inhibition assay (GIA), immune fluorescence assay (IFA) and cell-mediated immune (CMI) responses to parasites with typed AMA-1 |

| | |
|---|---|
| **Description of Study Design:** | Randomized, controlled Phase 2 trial of the FMP2.1/AS02A malaria vaccine, using rabies vaccine as a control. Four hundred subjects will be randomized in a 1:1 ratio to receive either 50 µg of FMP2.1 in 0.5 mL AS02A or rabies vaccine. Immunizations will be given on days 0, 30 and 60. Solicited adverse events will be recorded on the days of immunization and at 1, 2, 3 and 7 days after each immunization. Unsolicited adverse events will be recorded for 30 days after each immunization. Passive case detection will be used to capture clinical malaria episodes and adverse events including SAEs, and will occur by continuous availability of clinical care in a population with high utilization of this care. Active surveillance will be used to capture malaria infections and adverse events including SAEs. For active case detection, following the third dose, participants will be followed monthly for six months and then at 12, 18 and 24 months after randomization, for clinical assessment, malaria smear and hemoglobin. Routine monthly malaria smears will not be read immediately unless symptoms are present. Children will be followed for two years after the first immunization. Sera will be collected for anti-FMP2.1 antibody titers on the days of immunization and one, three, six, eight, 12, 18 and 24 months after the first immunization. PBMCs will be collected on the days of immunization, 30 days after the third immunization and eight, 12, 18 and 24 months after the first immunization. The study Final Report will be based on data collected up to six months after the assigned date of the third immunization. A supplemental report will include data from the entire 24 month observation period. |
| **Estimated Time to Complete Enrollment:** | 45 days |

**Figure 1 Study schema**

| Study phase | Study day |
|---|---|
| Screening | -45 to -1 |
| Immunizations | 0<br>30<br>60 |
| Primary efficacy follow up | 90<br>120<br>150<br>180<br>210<br>240 |
| Extended follow up | 364<br>547<br>730 |

## Key Roles

For questions regarding this protocol, contact:

Christopher V. Plowe MD MPH, Professor
Center for Vaccine Development
University of Maryland School of Medicine
685 West Baltimore Street, HSF 480
Baltimore, Maryland 21201 USA
Tel 410-706-3082, Fax 410-706-6205
E-mail: cplowe@medicine.umaryland.edu

**Individuals:**

**DMID Protocol Champion:**
Abdi Naficy MD, MPH, Medical Officer
Parasitology & International Programs Branch
Division of Microbiology & Infectious Diseases
National Institute of Allergy & Infectious Diseases
National Institutes of Health
6610 Rockledge Drive, MSC 6603
Bethesda, MD 20892, USA
Tel: (301) 435 2866, Fax: (301) 402 0659
E-mail: anaficy@niaid.nih.gov

**IND Sponsor:**
COL Jerome Pierson, Sponsor's Representative
Commander, US Army Medical Materiel Development Activity
1430 Veterans Drive
Fort Detrick, MD 21702
Tel: (301) 619-7643
E-mail: Jerome.pierson@us.army.mil

**Investigator Roles and Responsibilities:**

[1]Supervision of clinical trial conduct
[2]Participant enrollment, follow-up and care
[3]Study design and planning, protocol development, data analysis
[4]Clinical laboratory testing
[5]Specimen collection and transport
[6]Primary immunogenicity assays
[7]Additional analyses
[8]Vaccine handling and preparation
[9]Vaccine administration
[10]Severe adverse event reporting

**Principal Investigator:**

Mahamadou A. Thera MD, MPH, Associate Professor[1,2,3,5,7,10]
Malaria Research and Training Center
Department of Epidemiology of Parasitic Diseases
Faculty of Medicine, Pharmacy and Dentistry
University of Bamako
BP 1805, Point G
Bamako, Mali, West Africa
Tel/Fax 223-222-8109
E-mail: mthera@mrtcbko.org

**Co-Principal Investigator:**
Christopher V. Plowe MD MPH, Professor[1,3,7,9,10]
Center for Vaccine Development
University of Maryland School of Medicine
685 West Baltimore Street, HSF 480
Baltimore, Maryland 21201 USA
Tel 410-706-3082, Fax 410-706-6205
E-mail: cplowe@medicine.umaryland.edu

**Clinical Investigators:**
*University of Bamako*
Ogobara K. Doumbo MD, PhD, Professor and Director[1,2,3,7]
Dapa A. Diallo MD, Professor[1,2,3,7]
Drissa Coulibaly MD, Research Associate[1,2,3,5,7]
Abdoulaye Koné MD, Research Associate[2,3,5,7]
Ando Guindo MD, Research Associate[2,3,5,7]
Karim Traoré MD, Research Associate[2,3,5,7]
Drissa Traore MD candidate, Fellow[2,3,5,7]
Malaria Research and Training Center
Department of Epidemiology of Parasitic Diseases
Faculty of Medicine, Pharmacy and Dentistry
University of Bamako
BP 1805, Point G
Bamako, Mali, West Africa
Tel/Fax: + 223-222 81 09; 221 63 61; 222 49 87

*University of Maryland*
Kirsten E. Lyke MD, Assistant Professor[1,3,5,7,9]
Matthew B. Laurens MD MPH, Fellow[2,3,5,7]
Center for Vaccine Development
University of Maryland School of Medicine
685 West Baltimore Street, HSF 480
Baltimore, Maryland 21201 USA
Tel 410-706-5328, Fax 410-706-6205

*WRAIR*
(WRAIR clinical investigators may be on site during parts of the trial in an advisory capacity but will not be directly responsible for clinical aspects of the trial)
D. Gray Heppner MD[3,7]
James F. Cummings MD[3]
Michele D. Spring MD[3] [Contractor]
Department of Immunology
503 Robert Grant Avenue
Silver Spring, MD 20910
Contact: D. Gray Heppner MD
Tel 301-319-9414, Fax 301-319-7358

**Laboratory Investigators:**

*University of Bamako*
Mounirou Baby PharmD, Associate Professor, Clinical Lab Manager[3,4,5,7]
Amadou Niangaly PharmD, Research Associate[3,4,5,7]
Mady Cissoko PharmD, Research Associate, Clinical Lab Supervisor[3,4,5,7]
Amagana Dolo PharmD, PhD, Professor, Study Pharmacist[3,8]
Modibo Daou PharmD, Research Associate, Assistant Pharmacist[3,8]
Bourema Kouriba PharmD, PhD, Associate Professor[3,5,7]
Issa Diarra PharmD, Research Associate[3,4,7]
Malaria Research and Training Center
Department of Epidemiology of Parasitic Diseases
Faculty of Medicine, Pharmacy and Dentistry
University of Bamako
BP 1805, Point G
Bamako, Mali, West Africa
Tel/Fax: + 223-222 81 09; 221 63 61; 222 49 87

*University of Maryland*
Marcelo B. Sztein MD, Professor, for cellular immunology assays[3,7]
Shannon L. Takala PhD, for molecular assays[7]
Amed Ouattara PharmD, for molecular assays[7]
Center for Vaccine Development
University of Maryland School of Medicine
685 West Baltimore Street, HSF 480
Baltimore, Maryland 21201 USA
Tel 410-706-5328, Fax 410-706-6205

*WRAIR*
V. Ann Stewart DVM PhD, for primary immunogenicity assays[3,6,7]
Evelina Angov PhD, for additional laboratory assays[3,7]
Elke Bergmann-Leitner, PhD, for additional laboratory assays[3,7]

David Lanar PhD, for additional laboratory assays[3,7]
Sheetij Dutta PhD, for additional laboratory assays[3,7]
Christian Ockenhouse MD PhD, for additional laboratory assays[3,7]
Department of Immunology
503 Robert Grant Avenue
Silver Spring, MD 20910
Contact: D. Gray Heppner MD
Tel 301-319-9414, Fax 301-319-7358

**Co-Sponsors:**

*USAID*

(USAID provides financial support for the trial)

Carter Diggs MD PhD[3]
Tel 202-712-5728
Lorraine Soisson PhD[3]
Tel 908-369-7704
USAID Malaria Vaccine Development Program

*GSK*

(GSK provides the adjuvant for the trial)

Olivier Godeaux MD[3]
Clinical Development
GSK Biologicals
89 Rue de l'Institut
1330, Rixensart, Belgium
Tel +32 656 6910 Fax +32-2-656-6160
E mail: olivier.godeaux@gskbio.com

**Statistical Consultant:**

Don Stablein, PhD

(NIH contractor will provide data management system and statistical support)

The EMMES Corporation
401 N. Washington St., Suite 700
Rockville, MD 20850
Tel: 301-315-6145, Fax: 301-251-1355
Email: dstablein@emmes.com

**Clinical Trial Coordinator:**

Drissa Coulibaly MD
Malaria Research and Training Center
Department of Epidemiology of Parasitic Diseases
Faculty of Medicine, Pharmacy and Dentistry
University of Bamako
BP 1805, Point G
Bamako, Mali, West Africa

Tel/Fax: + 223-222 81 09; 221 63 61

**Data Quality Manager:**
Issaka Sagara MD MSPH
Malaria Research and Training Center
Department of Epidemiology of Parasitic Diseases
Faculty of Medicine, Pharmacy and Dentistry
University of Bamako
BP 1805, Point G
Bamako, Mali, West Africa
Tel/Fax: + 223-222 81 09; 221 63 61; 222 49 87

**Regulatory Affairs Specialists:**
(Coordination of communications with sponsors and IRBs)
Karen Ball RN MS
Center for Vaccine Development
University of Maryland
685 West Baltimore Avenue
Baltimore MD 21201
Phone: 410-706-0431, Fax: 410-706-4171
E-mail: kball@medicine.umaryland.edu

H. Marc Howell PhD (communications with FDA)
Division of Regulatory Affairs
U. S. Army Medical Materiel Development Activity
1430 Veterans Drive
Fort Detrick, MD 21702
Phone : 301-619-0317, Fax 301-619-0197
E-mail : marc.howell@amedd.army.mil

Lisa Ware
Department of Immunology
503 Robert Grant Avenue
Silver Spring, MD 20910
Tel: (301) 319-9941, Fax: (301) 319-7358
E-mail: lisa.ware@us.army.mil

**USAMMDA Medical Monitor:**
Lieutenant Colonel Arthur G. Lyons
WRAIR
Division of CD&I, Room 3A04
Silver Spring, MD 20910
Tel: (301) 319-9021
E-mail: Arthur.lyons@amedd.army.mil

**DMID Medical Monitor:**
Mirjana Nesin MD
Office of Clinical Research Affairs (OCRA)
Division of Microbiology & Infectious Diseases
National Institute of Allergy & Infectious Diseases
National Institutes of Health
6610 Rockledge Drive
Bethesda, MD 20892, USA
Tel: (301) 496 7067
E-mail: nesinm@niaid.nih.gov

**Local Medical Monitor**
Mariam Traore MD
Chief, Pediatric Service
Mopti Regional Hospital
Mopti, Mali
Tel: 223 601 0130

**Institutions:** University of Bamako
Malaria Research and Training Center
Department of Epidemiology of Parasitic Diseases
Faculty of Medicine, Pharmacy and Dentistry
BP 1805, Point G
Bamako, Mali, West Africa
Contact: Mahamadou A. Thera MD MPH
Tel/Fax 223-222-8109
E-mail: mthera@mrtcbko.org

University of Maryland School of Medicine
Center for Vaccine Development
685 West Baltimore Street, HSF 480
Baltimore, Maryland 21201 USA
Contact: Christopher V. Plowe MD MPH
Tel 410-706-3082, Fax 410-706-6205
E-mail: cplowe@medicine.umaryland.edu

Walter Reed Army Institute of Research
Division of Malaria Vaccine Development
503 Robert Grant Avenue
Silver Spring, MD 20910
Contact: James F. Cummings MD
Tel 301-319-9414, Fax 301-319-7358
E-mail: james.cummings@na.amedd.army.mil

The EMMES Corporation (NIH contractor for data and statistical support)
401 N. Washington St., Suite 700
Rockville, MD 20850
Tel: 301-315-6145, Fax: 301-251-1355
Email: mwolff@emmes.com

Office of the Surgeon General, US Army
US Army Medical Materiel Development Activity
1430 Veterans Drive
Fort Detrick, MD 21702
Contact: Colonel Jerome Pierson
Tel: (301) 619-7643
E-mail: Jerome.pierson@us.army.mil

GlaxoSmithKline Biologicals
89 Rue de l'Institut
Rixensart 1330, Belgium
Contact: Amanda Leach MSc MRCPCH
Tel: +32-2-656-7788, Fax: +32-2-656-6160
E-mail: amanda.leach@gskbio.com

USAID Malaria Vaccine Program
Contact : Carter Diggs MD PhD
Tel 202-712-5728
E-mail: cdiggs@usaid.gov

# 1 BACKGROUND INFORMATION AND SCIENTIFIC RATIONALE

## 1.1 Background information

### Malaria parasite life cycle

Among the four species of Plasmodium that cause human malaria, *Plasmodium falciparum* is responsible for most disease and death. Its life cycle is complex. Anopheline mosquitoes inject sporozoites which travel to the liver and invade hepatocytes. About six to ten days later, each infected hepatocyte releases thousands of merozoites into the bloodstream. Within the red blood cells, *P. falciparum* merozoites develop into schizonts over 48 hours and produce 15-30 new merozoites, each able to invade other red cells. The blood stages of the infection produce clinical symptoms and malaria disease.

Particular to *P. falciparum* is its ability to modify the surface of the red blood cell in a way that the infected cells can adhere to the vascular endothelium and other tissues, where they cause disease. Parasite sequestration in various organs (brain, heart, liver, kidney, placenta) contributes to the pathogenesis of malarial disease. A small proportion of asexual parasites convert to sexual forms that transmit the infection to others through female Anopheline mosquitoes.

## 1.2 Disease burden

Africa bears the heaviest burden of malaria. *P. falciparum* is responsible for more than one million deaths worldwide each year. More than 90% of these deaths occur among sub-Saharan African children under five years of age. In areas of stable malaria transmission, 25% of all-cause mortality in children aged four years or less has been directly attributed to malaria (1). Studies done in West Africa indicate that malaria could account for as much as 60% of all-cause mortality in children aged less than five years old (2-4).

### Malaria in Mali

In Mali, West Africa, where childhood mortality exceeds 20%, malaria is a leading cause of morbidity in the general population and of mortality in children aged less than ten years. The study site, Bandiagara (pop. 13,364), is 700 km northeast of Bamako on the Dogon plateau. Here the malaria transmission season is from July through December, with peak transmission in September and peak disease incidence in October. About 85% of Bandiagara children aged 0-10 years have at least one clinical episode of uncomplicated malaria during the malaria season, and the average number of clinical episodes of malaria per child and per transmission season is two, with a few

children experiencing a maximum of four clinical episodes (5).The incidence of severe malaria among children aged six years or less in Bandiagara was 2.5% (n=2284) in 2000. (6).

## 1.3 Rationale

A safe and effective malaria vaccine would represent an important tool for malaria prevention and control. Two critical steps of the malaria life cycle have been closely examined as potential targets for vaccine-induced immunity: the invasion of liver cells by sporozoites and the invasion of red blood cells by merozoites. Vaccines directed against pre-erythrocytic stages including sporozoites are intended to prevent disease by blocking infection, and those directed against blood stage parasites are intended to prevent disease by inhibiting parasite replication in the blood.

The circumsporozoite protein (CSP), which coats the surface of the sporozoite, is critical for the invasion of liver cells and contains a peptide sequence that binds to the surface of liver cells (7). *P. falciparum* CSP is composed of a central repeating sequence (NANP) flanked by two non-repeat sequences. GlaxoSmithKline Biologicals (GSK) and the Walter Reed Army Institute of Research (WRAIR) developed a recombinant molecule that contains important T helper epitopes from CSP as well as the $(NANP)_{19}$ repeat fused to hepatitis B (8). A recent Phase 2b trial in more than 2000 children aged 1-4 years in Mozambique reported 30% efficacy against incidence of first clinical episodes of *P. falciparum* malaria and efficacy against severe malaria of 58% (9). The duration of protection against both clinical episodes and severe malaria was reported to have lasted at least 18 months in this trial (10).

To improve on the efficacy of RTS,S, WRAIR, GSK and other partners including the investigators for this protocol are developing other vaccines directed against blood-stage antigens using the same adjuvant platform. These vaccines may be combined with RTS,S in a multi-stage, multi-antigen vaccine to improve on the efficacy of RTS,S (11) and/or be developed as stand-alone malaria blood-stage vaccines appropriate for preventing malaria disease in endemic populations including African children.

### The AMA-1 antigen

The apical membrane antigen 1 (AMA-1) is a surface protein expressed during the asexual blood stage of *P. falciparum*. AMA-1 is produced as an 83-kDa polypeptide by mature schizonts in infected erythrocytes (12), and localizes in the microneme, an apical secretory organelle of the merozoite containing ligands for binding red cell receptors (13). The protein is processed to a 66-kDa protein that is subsequently exported to the merozoite surface where it plays a critical role in invading erythrocytes (14-17).

*P. falciparum* AMA-1 consists of a signal sequence, a large extracellular domain (ectodomain) of 546 amino acids), a transmembrane domain (21 amino acids), and a C-terminal cytoplasmic domain (55 amino acids) (18). Comparisons between all of the known amino acid sequences of AMA-1 homologues indicate greater than 50% sequence identity, with 16 cysteine residues conserved in all sequences (19-21). All of the cysteines are found in the ectodomain of the molecule, which is stabilized by eight intramolecular disulfide bonds (22).

Seroepidemiologic studies conducted in West Africa and western Kenya demonstrate that natural antibody responses to AMA-1 are widespread and highly prevalent (23). Serological and cellular immune responses to AMA-1 increase with age in populations subject to continued exposure to malaria infection (24). Preliminary studies in Mali demonstrate that natural antibodies exist in a large proportion of individuals (>90%) in this area of intense, seasonal transmission. In a cross-sectional study of 200 individuals aged six months to 45 years conducted in 2002 and 2003, median anti-AMA-1 antibody titers peaked coindicident with the peak of malaria transmission, although with significant variations in titer was seen between different age groups (25).

Naturally acquired antibodies to AMA-1 found in people living in malaria-endemic areas inhibit the growth of *P. falciparum* in vitro (26). This inhibition was dose-dependent and anti-AMA-1 antibodies recognized both strain-specific and conserved epitopes. From this evidence, it is reasonable to postulate that boosting the natural antibody response to AMA-1 through vaccination may protect an individual from illness due to the asexual blood stage of *P. falciparum* infection.

### Genetic diversity in AMA-1

In vitro experiments and studies in both animals and humans have indicated some degree of allele-specificity in the antibody responses to genetically different forms of AMA-1 (26-30). We have sequenced domain I of AMA-1 from 518 *P. falciparum* infections that occurred in 100 individuals participating in a prospective cohort study of malaria infection and disease in Bandiagara in 1999, 2000, and 2001. A very high degree of polymorphism in AMA-1 was observed within and between years. Thirty-seven polymorphic amino acid sites were identified, and 118 unique haplotypes or alleles (distinct genetic sequences of AMA-1 domain I) were observed among the 518 infections (31). The most common haplotype was identical to the 3D7 parasite strain of *P. falciparum* on which the FMP2.1 antigen is based. However, the 3D7 haplotype had a frequency of only about 10% each year; thus 90% of infections in Bandiagara were different from the vaccine strain with respect to one or more of the 37 polymorphic amino acid sites in domain I. The degree to which a 3D7-based vaccine will protect against non-3D7 type parasites in vivo is not known. To the extent possible, allele-specific efficacy will be investigated in this trial, although it is recognized that the small numbers of each haplotype result in limited statistical power for these analyses.

## 1.4 The FMP2.1/AS02A vaccine

FMP2.1 is a lyophilized preparation of the ectodomain of the 3D7 clone of *P. falciparum* AMA-1. FMP2.1 is comprised of 478 amino acids, 449 of which are derived from the merozoite surface protein AMA-1 of the malaria parasite, *P. falciparum*, 3D7 clone. The protein is produced in and

purified from *E. coli* bacteria at the WRAIR BioProduction Facility. The gene encoding the FMP2.1 protein was chemically synthesized to contain an *E. coli*-optimized codon usage to encode the amino acids representative of amino acids 83 to 531 of the AMA-1 protein from the 3D7 clone of *P. falciparum*. The purified antigen is adjuvanted with AS02A (proprietary oil-in-water emulsion and phosphate-buffered saline + MPL + QS21). This candidate vaccine is intended to limit malaria morbidity and mortality, and possibly infection, by stimulating host immune responses against the AMA-1 of *P. falciparum*.

Following a Phase 1 study in malaria-naïve US volunteers, the first Phase 1 study in a malaria-exposed population of the AMA-1-based vaccine, FMP2.1/AS02A, was completed in adults in Bandiagara in 2005. As detailed below, the vaccine was safe and well tolerated and showed a dose-dependent immune response in adults in Bandiagara. This was followed by a Phase 1 safety and immunogenicity study of three escalating doses of this vaccine in children, which began in November 2006 with completion of immunizations in February 2007. The present Phase 2 trial will add to the safety profile of the vaccine and provide the first assessment of vaccine efficacy. Demonstration of efficacy would then be followed by further development of an AMA-1 vaccine either alone or in a multi-antigen vaccine in combination with other malaria vaccines, possibly including RTS,S, and/or other genotypes of AMA-1.

### Preclinical toxicity, safety and reactogenicity of FMP2.1/AS02A

Clinical grade lots of FMP2.1 protein have been administered to mice, guinea pigs, rabbits, and rhesus monkeys. As summarized in the attached Investigators' Brochure, no safety concerns arose from preclinical studies.

### Clinical experience with FMP2.1/AS02A

FMP2.1/AS02A has been administered to a total of 72 adult volunteers and 75 children (Table 1) in two dose-escalation Phase 1 protocols in adults in the US and Mali, identified as Malaria-033 and Malaria-037 respectively, in a Phase 2a challenge study in US adults identified as Malaria-045, and in a Phase 1 pediatric protocol identified as Malaria-051. In Malaria-033, the three dosage levels (approximately 10 µg, 25 µg or 50 µg FMP2.1 in 0.5 mL AS02A) were found to be safe and well-tolerated in healthy malaria-naïve adults in the US In Malaria-037, two dosage levels (approximately 25 µg FMP2.1 in 0.25 mL AS02A and approximately 50 µg FMP2.1 in 0.5 mL AS02A) were tested and found to be safe and well-tolerated in healthy malaria-exposed adults in Mali.

**Table 1 Doses of AMA-1 FMP2.1/AS02A administered in clinical studies**

| Study Designation | ~50 µg FMP2.1 in 0.5 mL AS02A | | ~25 µg FMP2.1 in either 0.5 mL or 0.25 mL AS02A[1] | | ~10 µg FMP2.1 in 0.5 mL AS02A | | Total | |
|---|---|---|---|---|---|---|---|---|
| | Total number of subjects | Total number of doses | Total number of subjects | Total number of doses | Total number of subjects | Total number of doses | Total number of subjects | Total number of doses |

| | | | | | | | | |
|---|---|---|---|---|---|---|---|---|
| Malaria-033 | 7 | 19[2] | 8 | 22[3] | 8 | 22[4] | 23 | 63 |
| Malaria-037 | 20 | 59[5] | 20 | 60 | -- | -- | 40 | 119 |
| Malaria-045 | 15 | 43-45[6] | 0 | 0 | 0 | 0 | 15 | 43-45[6] |
| Malaria-051 | 30 | 27-30[7] | 30 | 25-30[7] | 15 | 14-15[7] | 75 | 66-75[7] |
| **TOTALS** | **72** | pending | **73** | pending | **23** | pending | **153** | pending |

[1] In Malaria-033 all doses of antigen were administered with a 0.5 mL AS02A. In Malaria-037 the doses were prepared using a fixed ratio of antigen to adjuvant (~25 µg FMP2.1 formulated in 0.25 mL AS02A and ~50 µg FMP2.1 formulated in 0.5 mL AS02A)

[2] Five of the seven volunteers in this group received three immunizations. Two volunteers received only two immunizations, one due to loss to follow-up and one due to being withdrawn after experiencing Grade 3 AEs as described below.

[3] Seven of the eight volunteers in this group received three immunizations. One volunteer received only one immunization, due to an SAE that was unrelated to vaccination as described below.

[4] Seven of the eight volunteers in this group received three immunizations. One volunteer received only one immunization due to loss to follow-up.

[5] Nineteen volunteers in this group received three immunizations. One volunteer received only two immunizations due to elevated ALT as described below.

[6] Two of 45 volunteers in this trial received only two doses, one due to scheduling conflict and one due to local reactogenicity.

[7] Up to 9 volunteers in this trial which is still blinded may have received fewer than 3 doses of FMP2.1/AS02A, 2 due to anemia on the scheduled day of immunization, 5 due to elevated ALT and/or documented hepatitis A, 1 due to heart murmur on day of immunization, and 1 due to refusal of blood draws.

Adverse events (AEs) and serious adverse events (SAEs) were defined in standard fashion as described in detail in the attached Investigator's Brochure.

## Phase 1 trial in US adults

Malaria-033: "Phase 1 dose escalation study to evaluate safety, reactogenicity, and immunogenicity of FMP2.1/AS02A in malaria-naïve adults in the US" was an open-label Phase 1, dose-escalation study to evaluate the safety, reactogenicity, and immunogenicity of FMP2.1 reconstituted with AS02A adjuvant. This study was initiated in September 2003 at the WRAIR Clinical Trials Center. Twenty three participants were enrolled and assigned to receive approximately 10 µg (N=8), 25 µg (N=8), or 50 µg (N=7) doses of antigen with 0.5 mL AS02A on a 0-, 1-, 2-month schedule by intramuscular injection. As shown in Table 2, in the 10 µg and 25 µg dosage groups, 22 of the 24 scheduled doses were given; seven of the eight volunteers in each of these groups received all three immunizations with the eighth volunteer receiving only one immunization. In the 50 µg dosage group, 19 of the 21 scheduled doses were given; five of the seven volunteers in this group received all three immunizations with the other two volunteers receiving two immunizations. All three doses were found to be safe and well tolerated. FMP2.1/AS02A was immunogenic in all volunteers.

### Safety/reactogenicity data

AEs were assessed for intensity. Injection site pain was graded as 0 = absent, 1 = painful on touch, 2 = painful when limb is moved, and 3 = spontaneously painful. Solicited symptoms were graded as

0 = normal, 1= easily tolerated, 2 = interferes with normal activity, and 3 = prevents normal daily activity. Additional grading scales were applied to visible swelling at the injection site; 0 = none, 1= >0 to 20 mm, 2 = >20 to 50 mm, and 3 = > 50 mm, and to oral temperature; 0 = < 37.5 $^{o}$C, 1 = 37.5 to 38 $^{o}$C, 2 = >38 to 39 $^{o}$C, and 3 = >39 $^{o}$C.

All vaccine-related AEs occurred within 72 hours after vaccination (Table 2). Ten Grade 3 reactions were reported with seven of ten occurring in the 50 µg dosage group and the remaining three occurring in the 25 µg dosage group (Table 2). Five of the Grade 3 reactions occurred in one individual in the 50 µg group after the second vaccination and were self-reported, as the individual did not return for follow-up during the time he was symptomatic. Local pain (43 incidents over 63 vaccinations), local swelling (8 incidents over 63 vaccinations) and headache (12 incidents over 63 vaccinations) accounted for most of the solicited AEs (Table 3).

There was one SAE reported during the study, an episode of paroxysmal supraventricular tachycardia that occurred two days following the first immunization in a volunteer in the 25 µg dosage group. It was determined the volunteer had had similar, unreported symptoms prior to entry into the study. She was evaluated by cardiology consultants and underwent extensive testing including electrocardiogram, blood testing, echocardiogram, nuclear medicine study and cardiac MRI. There was no evidence of structural abnormality, myocarditis, or pericarditis. Additionally, the case was reviewed by the Director of Cardiac Electrophysiology at Walter Reed Army Medical Center. The cardiology consultants concluded the event was consistent with paroxysmal supraventricular tachycardia that predated enrollment in the study and was not related to immunization. The volunteer was withdrawn from the study by the Principal Investigator. On follow up she was asymptomatic until seven months after vaccination when she experienced the same symptoms after placement of a purified protein derivative (PPD) for routine tuberculosis screening.

**Table 2 Summary of incidence of adverse events (Malaria-033)**

| Group | Grade 1 | | Grade 2 | | Grade 3 | |
|---|---|---|---|---|---|---|
| | First 72 hours after vaccinations | Day 7 | First 72 hours after vaccinations | Day 7 | First 72 hours after vaccinations | Day 7 |
| ~10 µg FMP2.1 in 0.5 mL AS02A | 11 | 0 | 6 | 0 | 0 | 0 |
| ~25 µg FMP2.1 in 0.5 mL AS02A | 17 | 0 | 25 | 0 | 3 | 0 |
| ~50 µg FMP2.1 in 0.5 mL AS02A | 23 | 0 | 14 | 0 | 7[1] | 0 |
| **Total** | **51** | **0** | **45** | **0** | **10** | **0** |

[1] Five of the seven Grade 3 reactions in this group were self-reported by one individual after second vaccination.

**Table 3 Participants with at least one AE (Malaria-033)**

| **Group** | |
|---|---|

| | **Local AE** No. subjects (%) | **General AE** No. subjects (%) | **Both Local & General AE** No. subjects (%) |
|---|---|---|---|
| **Group A** 10 µg FMP2.1 in 0.5 mL AS02A (n=8) | 6 (75.0%) | 3 (37.5%) | 2 (25.0%) |
| **Group B** 25 µg FMP2.1 in 0.5 mL AS02A (n=7) | 7 (100%) | 6 (85.7%) | 6 (85.7%) |
| **Group C** 50 µg FMP2.1 in 0.5 mL AS02A (n=7) | 7 (100%) | 7 (100%) | 7 (100%) |

**Table 4 Solicited adverse event totals during 7 days after immunizations (Malaria-033)**

| | ~10 µg FMP2.1 in 0.5 mL AS02A | | | ~25 µg FMP2.1 in 0.5 mL AS02A | | | ~50 µg FMP2.1 in 0.5 mL AS02A | | |
|---|---|---|---|---|---|---|---|---|---|
| | Grade1 | Grade2 | Grade3 | Grade1 | Grade2 | Grade3 | Grade1 | Grade2 | Grade3 |
| Pain | 6 | 4 | 0 | 6 | 13 | 1 | 6 | 7 | 0 |
| Redness | 0 | 0 | 0 | 0 | 0 | 1 | 2 | 0 | 0 |
| Swelling | 1 | 1 | 0 | 1 | 1 | 1 | 3 | 0 | 0 |
| Fever | 0 | 0 | 0 | 0 | 0 | 0 | 0 | 2 | 0 |
| GI | 0 | 0 | 0 | 2 | 0 | 0 | 0 | 0 | 0 |
| Headache | 2 | 0 | 0 | 2 | 3 | 0 | 2 | 1 | 2 |
| Malaise | 0 | 0 | 0 | 1 | 2 | 0 | 1 | 0 | 2 |
| Myalgia | 2 | 1 | 0 | 3 | 3 | 0 | 2 | 1 | 1 |
| Fatigue | 0 | 0 | 0 | 2 | 2 | 0 | 2 | 3 | 1 |
| Arthralgia | 0 | 0 | 0 | 0 | 1 | 0 | 2 | 0 | 1 |
| **Total** | **11** | **6** | **0** | **17** | **25** | **3** | **23** | **14** | **7** |

**Immunogenicity data**

There was a robust three-log anti-AMA-1 antibody response to all three vaccine dosages as determined by ELISA (Table 5 and Figure 2). After the second immunization, the antibody response in all three dosage groups was similar with no statistical differences between groups by analysis of variance. Increases in antibody titer were most pronounced after the first and second immunizations, with only a marginal increase after the third immunization. During the six months of follow-up after the third immunization, antibody titers decayed slightly less than one log. The

antibody response was consistent in all individuals. The average coefficient of variation of log titer at all subsequent time points after the second injection was less than 10% for all vaccine groups.

**Table 5 Anti-FMP2.1 Geometric Mean Antibody Titers by ELISA (Malaria-033 [Malaria-Naïve Adults])**

| Vaccine Group | Day 0[1] | Day 14 | Day 28[1] | Day 42 | Day 56[1] | Day 70 | Day 112 | Day 168 | Day 224 |
|---|---|---|---|---|---|---|---|---|---|
| ~10 µg FMP2.1 in 0.5mL AS02A | 15 | 55 | 290 | 52464 | 40591 | 93149 | 42792 | 18125 | 10212 |
| ~25 µg FMP2.1 in 0.5mL AS02A | 34 | 411 | 1181 | 72424 | 48827 | 92243 | 49495 | 25732 | 14888 |
| ~50 µg FMP2.1 in 0.5mL AS02A | 14 | 379 | 2827 | 73097 | 51216 | 116069 | 71707 | 31046 | 16754 |

[1] Immunizations were given on days 0, 28, and 56.

**Figure 2 Anti-FMP2.1 Antibody Response by ELISA (log scale) (Malaria-033)**

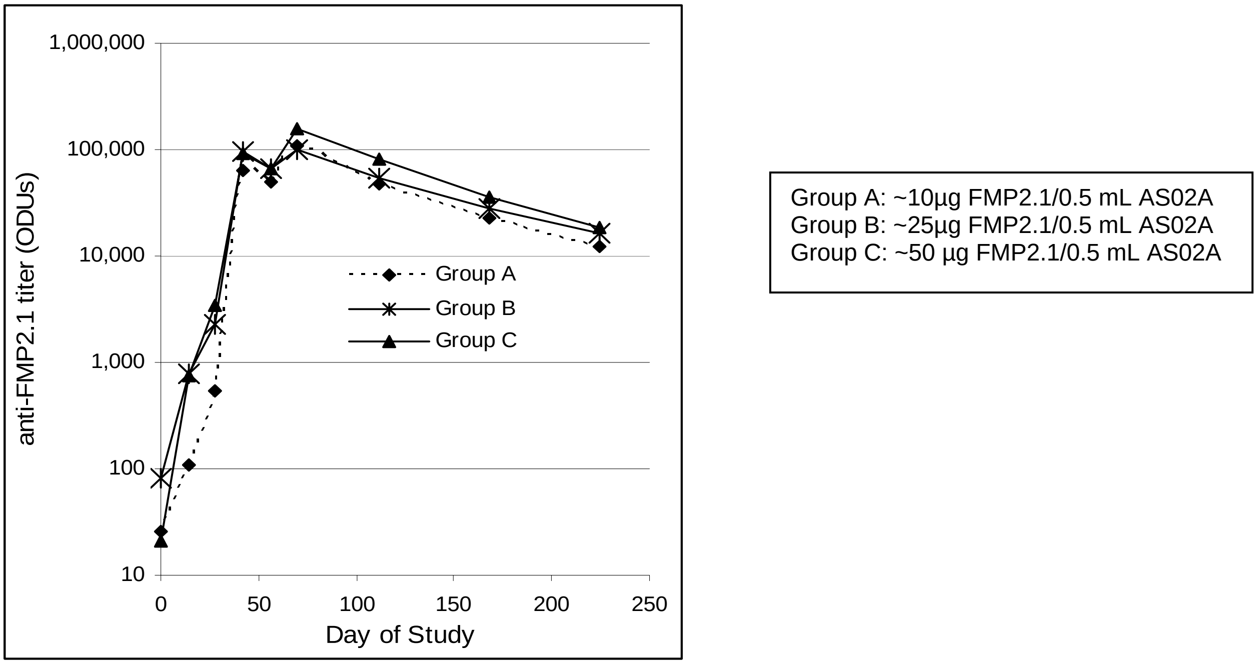


## Phase 1 trial in Malian adults

Based on the safety and immunogenicity results of Malaria-033, a protocol entitled "Double-blind, randomized, controlled Phase 1 dose escalation trial to evaluate the safety and immunogenicity of the WRAIR AMA-1 malaria antigen (FMP2.1) formulated in GSK's AS02A vs. rabies vaccine in

malaria-experienced adults in Bandiagara, Mali" (Malaria-037) began in November 2004. Sixty adults were randomized in a 2:1 fashion in two staggered cohorts of 30 to receive by deltoid intramuscular injection approximately 50 µg FMP2.1 in 0.5 mL AS02A (n=20) or rabies vaccine (n=10) (cohort 1); or approximately 25 µg FMP2.1 in 0.25 mL AS02A (n=20) or rabies vaccine (n=10) (cohort 2). Thus a fixed antigen:adjuvant ratio was used in Malaria-037 as has been done for subsequent trials, in contrast to Malaria-033 which used a fixed volume of adjuvant. The immunizations were administered on study days 0, 30, and 60.

One hundred seventy-five adults were screened and 60 healthy adults aged 18-55 were enrolled and immunized. Solicited symptoms were actively monitored for eight days after immunization. Subjects were queried regarding local signs and symptoms including injection site pain, erythema, swelling and arm motion limitation; and general systemic signs and symptoms including headache, fever, chills, nausea, myalgia, joint pain and malaise. Unsolicited symptoms were monitored for 30 days after each immunization, and SAEs were monitored for one year after the first immunization. Antibody titers to FMP2.1 were measured by ELISA on sera collected on study day 0 and 14, and 30 days after each immunization, as well as six, nine, and 12 months after the first immunization. All 60 participants received the first two immunizations and 59 participants received all three immunizations. One person in the FMP2.1 50 µg group had his third dose withheld due to a previous elevated ALT, described in the next section. Two participants were lost to follow-up, one shortly after receiving the third immunization and one before the final study visit. Both of those participants were from the 25 µg group and received all three doses of FMP2.1.

This study found that both dosage levels of FMP2.1/AS02A were safe and well tolerated by malaria-experienced adults in Bandiagara, and that the vaccine produced a significant, dose-dependent antibody response.

**Safety/reactogenicity data**

AEs were assessed for intensity. Grading scales used to determine the intensity of the following adverse events are described in Table 6.

- Grade 0 = No adverse event.
- Grade 1 = An adverse event that is easily tolerated by the subject, causing minimal discomfort and not interfering with everyday activities.
- Grade 2 = An adverse event that is sufficiently discomforting to interfere with normal everyday activities.
- Grade 3 = An adverse event that prevents normal, everyday activities. Such an adverse event would for example prevent attendance at work/school and would require the administration of corrective therapy.

**Table 6 Assessment of adverse event intensity**

| Adverse Event | Intensity grade | Intensity definition |
|---|---|---|
| Pain at injection site | 0 | None |
| | 1 | Pain that is easily tolerated |
| | 2 | Pain that interferes with daily activity |
| | 3 | Pain that prevents daily activity |
| Swelling at injection site<br>Record size | 0<br>1<br>2<br>3 | 0 mm<br>>0 - ≤ 20 mm<br>>20 - ≤ 50 mm<br>>50 mm |
| Erythema at injection site<br>Record size | 0<br>1<br>2<br>3 | 0 mm<br>>0 - ≤ 20 mm<br>>20 - ≤ 50 mm<br>>50 mm |
| Limitation of arm motion - Abduction at the shoulder | 0 | None |
| | 1 | >90° but <120° |
| | 2 | >30° but ≤90° |
| | 3 | ≤30° |
| Fever<br>Record oral temperature | 0<br>1<br>2<br>3 | <37.5ºC<br>37.5 - ≤38.0ºC<br>>38.0 - ≤39ºC<br>>39ºC |
| Chills | 0 | None |
| | 1 | Chills that are easily tolerated |
| | 2 | Chills that interfere with daily activity |
| | 3 | Chills that prevent daily activity |
| Nausea | 0<br>1<br>2<br>3 | None<br>Nausea that is easily tolerated<br>Nausea that interferes with daily activity<br>Nausea that prevents daily activity |
| Headache | 0 | None |
| | 1 | Headache that is easily tolerated |
| | 2 | Headache that interferes with daily activity |
| | 3 | Headache that prevents daily activity |
| Malaise | 0 | None |
| | 1 | Malaise that is easily tolerated |
| | 2 | Malaise that interferes with daily activity |
| | 3 | Malaise that prevents daily activity |
| Myalgia | 0 | None |
| | 1 | Myalgia that is easily tolerated |
| | 2 | Myalgia that interferes with daily activity |
| | 3 | Myalgia that prevents daily activity |
| Joint pain | 0 | None |
| | 1 | Joint pain that is easily tolerated |
| | 2 | Joint pain that interferes with daily activity |
| | 3 | Joint pain that prevents daily activity |

Solicited Grade 3 symptoms, which consisted entirely of local injection site swelling of greater than 5 cm across at the widest dimension, were more common in the 50 μg group than in the 25 μg group or control group (Table 8). This swelling did not interfere with normal daily activities and was usually unnoticed by the participants and detected by careful physical examination. AEs were balanced by group and were typical of common medical problems in Bandiagara (Table 7). Laboratory toxicities were graded using the DMID guidelines (available at: http://www.niaid.nih.gov/dmid/clinresearch/). One alanine aminotransferase (ALT) elevation to 562

U/L was detected in an individual in the 50 µg FMP2.1/AS02A group who had negative serological testing for viral hepatitis. The participant had been using a non-steroidal anti-inflammatory drug that is associated with elevated liver transaminases, and after peaking on study day 37 the ALT was normal on study day 60. In the opinion of the investigators and the local medical monitor this was not considered to be related to vaccination, but a decision was made to withhold the third vaccination from this individual. There were mild, clinically insignificant abnormalities in complete blood count (CBC), creatinine and ALT that were balanced by group. No SAEs were reported through study day 364.

**Table 7 Summary of Incidence of Adverse Events (Malaria-037)**

| Group | Grade 1 | Grade 2 | Grade 3 |
|---|---|---|---|
| | First 7 days after vaccinations | First 7 days after vaccinations | First 7 days after vaccinations |
| ~25 µg FMP2.1 in 0.25 mL AS02A | 191 | 32 | 6 |
| ~50 µg FMP2.1 in 0.5 mL AS02A | 191 | 26 | 21 |
| Rabies vaccine (RabAvert®) | 142 | 4 | 5 |
| **Total** | **524** | **62** | **32** |

**Table 8 Solicited Adverse Event Totals (Malaria-037)**

| | ~25 µg FMP2.1 in 0.25 mL AS02A | | | ~50 µg FMP2.1 in 0.5 mL AS02A | | | Rabies vaccine (RabAvert®) | | |
|---|---|---|---|---|---|---|---|---|---|
| | Grade1 | Grade2 | Grade3 | Grade1 | Grade2 | Grade3 | Grade1 | Grade2 | Grade3 |
| Pain | 44 | 1 | 0 | 49 | 4 | 0 | 22 | 0 | 0 |
| Erythema | 0 | 0 | 0 | 0 | 0 | 0 | 0 | 0 | 0 |
| Swelling | 3 | 22 | 6 | 0 | 10 | 21 | 3 | 2 | 5 |
| Limitation of arm movement | 4 | 1 | 0 | 3 | 0 | 0 | 0 | 0 | 0 |
| Headache | 7 | 0 | 0 | 13 | 4 | 0 | 9 | 0 | 0 |
| Fever (Oral T >37.5$^{o}$C) | 6 | 0 | 0 | 6 | 0 | 0 | 1 | 0 | 0 |
| Malaise | 4 | 0 | 0 | 4 | 1 | 0 | 3 | 0 | 0 |
| Myalgia | 3 | 0 | 0 | 5 | 1 | 0 | 3 | 0 | 0 |
| Arthralgia | 0 | 0 | 0 | 1 | 1 | 0 | 1 | 0 | 0 |
| Chills | 4 | 0 | 0 | 2 | 0 | 0 | 1 | 0 | 0 |
| Nausea | 4 | 0 | 0 | 1 | 0 | 0 | 1 | 0 | 0 |
| **Total** | **86** | **26** | **6** | **90** | **23** | **21** | **47** | **3** | **5** |

**Figure 3 Incidence of Grade 3 solicited symptoms during the eight-day follow-up period after third immunization (Malaria-037)**

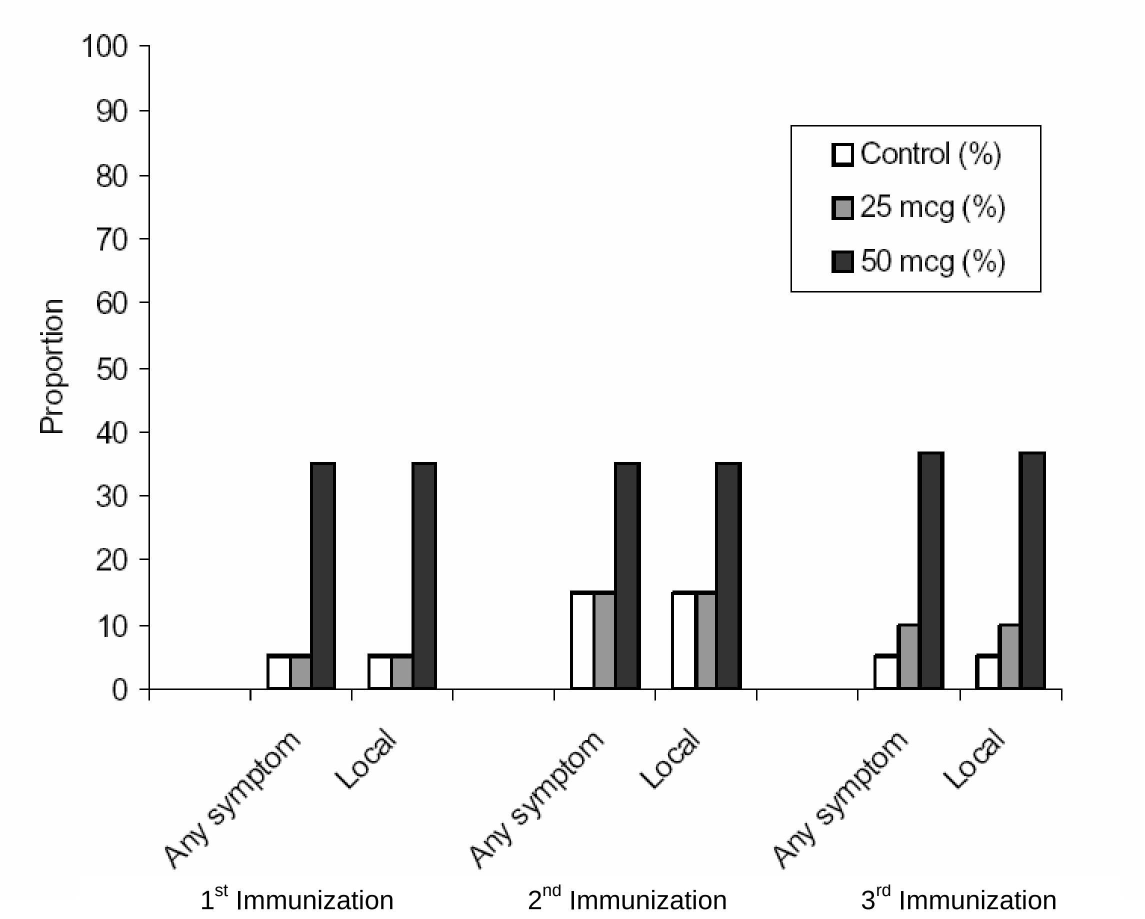


**Immunogenicity data**

**Humoral Immune Response**: Volunteers receiving either the 25 or 50 µg dose of FMP2.1 developed anti-FMP2.1 antibody responses that were significantly higher than the rabies vaccinated volunteers. Two weeks after the third immunization there were 6- and 11- fold increases in anti-FMP2.1 titers respectively in the 25 and 50 µg dosage groups over the rabies control group. After the second immunization there was a significant difference between the geometric mean titers of the FMP2.1 vaccinated individuals and the rabies vaccinated individuals achieved significant difference ($p<0.05$ on log-transformed data). There was a trend, but no significant difference between the 25 and 50 µg dosage groups after the second immunization ($p=0.07$) (Table 9). Similar to the malaria-naïve volunteers immunized in Malaria-033, the coefficients of variance for the log transformed data from the malaria-exposed volunteers immunized in Malaria-037 were 10.1% or less after the second immunization whereas in the rabies vaccinated individuals the average coefficient of variance was 20.9% and did not vary significantly over time (Figure 4). The baseline antibody titers were higher in this malaria-exposed population than in the malaria-naïve participants in Malaria-033, but the magnitude of the post-immunization responses was similar.

**Table 9 Anti-FMP2.1 Geometric Mean Antibody Titers by ELISA (Malaria-037 [Malaria-Exposed Adults])**

| | Day 0[1] | Day 14 | Day 30[1] | Day 44 | Day 60[1] | Day 74 | Day 90 | Day 180 |
|---|---|---|---|---|---|---|---|---|
| ~25 µg FMP2.1 in 0.25 mL AS02A | | 30794 | 31805 | 55246 | 61439 | 89857 | 60967 | 50750 |
| ~50 µg FMP2.1 in 0.5 mL AS02A | 23500 | 69752 | 55086 | 100590 | 119191 | 150738 | 102813 | pending |
| Rabies vaccine (RabAvert®) | 14355 | 13857 | 12691 | 13941 | 16030 | 14064 | 10425 | 7846 |

**Figure 4 Anti-FMP2.1 Antibody Response by ELISA (log scale) (Malaria-037). Immunizations were performed on days indicated by arrows.**

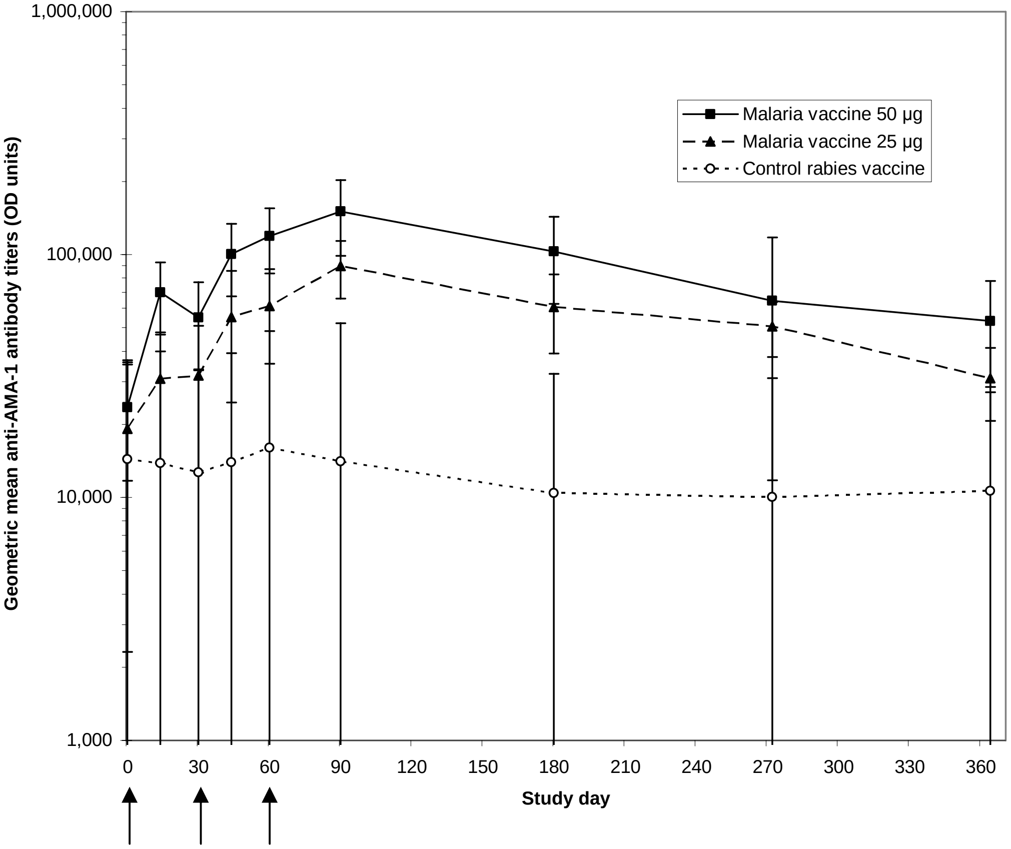


## Phase 1 trial in Malian children

As the next step in the development of this vaccine, a Phase 1 trial in children was begun in Mali in November 2006. This trial, entitled "Randomized, Controlled, Dose Escalation Phase 1 Clinical Trial To Evaluate The Safety And Immunogenicity Of WRAIR's AMA-1 Malaria Vaccine (FMP2.1) Adjuvanted In GSK's AS02A Vs. Rabies Vaccine In 1-6 Year Old Children In Bandiagara, Mali" is

presently in progress. The data included in this protocol are cumulative safety data for this study, now available through Day 30 after the third and final vaccination for all participants. The study remains blinded pending performance of post-vaccination immunogenicity assessments. The DSMB, which will also serve as the DSMB for the present Phase 2 study, reviewed these data on 23 March 2007 and recommended proceeding with this Phase 2 trial.

### Study design

This study is a randomized, controlled dose-escalation Phase 1 trial of the FMP2.1/AS02A malaria vaccine, using rabies vaccine as a control. Children aged 1-6 years were randomized to three dose escalation cohorts. Twenty participants were enrolled in Cohort 1, 40 participants were enrolled in Cohort 2, and 40 in Cohort 3. Children within each cohort were stratified by age and randomized in a 3:1 ratio to receive 10, 25 or 50 μg of FMP2.1 (in Cohorts 1, 2 and 3, respectively) adjuvanted vaccine with a proportionate volume of the AS02A, or rabies vaccine. Thus, a total of 75 children have received the malaria vaccine and 25 the rabies vaccine. Immunizations were given on days 0, 30 and 60 in a staggered fashion, with the first administrations of the 25 and 50 μg dose levels of FMP2.1 following the first administration of the 10 and 25 μg dose levels, respectively, by 2-3 weeks.

Solicited adverse events were recorded on the days of immunization and 1, 2, 3 and 7 days after each immunization, and unsolicited adverse events were recorded for 30 days after each immunization. Children will be followed for one year after the last immunization to record SAEs and measure the dynamics of immune responses. Sera are collected for anti-FMP2.1 antibody titers on the days of immunization and 14 days after each immunization, as well as three, six, nine and 12 months after the first immunization.

**Figure 5 Schematic of study design**

| | | | |
|---|---|---|---|
| Cohort 1 | ARM 1 | N=15 | FMP2.1/AS02A 10 μg dose |
| | ARM 2 | N=5 | Rabies vaccine |

↓

Interim blinded safety evaluation by DSMB after 1st 10 μg dose

↓

| | | | |
|---|---|---|---|
| Cohort 2 | ARM 1 | N=30 | FMP2.1/AS02A 25 μg dose |
| | ARM 2 | N=10 | Rabies vaccine |

↓

Interim blinded safety evaluation by DSMB after 1st 25 μg dose

↓

| | | | |
|---|---|---|---|
| Cohort 3 | ARM 1 | N=30 | FMP2.1/AS02A 50 μg dose |
| | ARM 2 | N=10 | Rabies vaccine |

### Methods for safety assessment

All adverse events occurring within 30 days following administration of each study immunization were recorded irrespective of severity or whether or not they are considered related to vaccination. Solicited adverse events were elicited for a seven-day surveillance period (day of vaccination and

study days 1, 2, 3 and 7) and unsolicited adverse events are recorded during a 30-day surveillance period. SAEs are recorded throughout the study.

Solicited systemic AEs include: fever (axillary temperature ≥ 37.5°C); drowsiness, loss of appetite; irritability/fussiness; and vomiting. Solicited injection site AEs include: pain/tenderness; induration/swelling; and erythema (redness).

For events not included in the protocol-defined severity grading criteria, the following guidelines are used to categorize severity. Intensity is assigned to one of the following categories:

0 = No adverse event

1 = An adverse event that is easily tolerated by the participant, causing minimal discomfort and not interfering with everyday activities.

2 = An adverse event that is sufficiently discomforting to interfere with normal everyday activities.

3 = An adverse event that prevents normal, everyday activities. Such an adverse event would, for example, prevent normal play or attendance at school and would require the administration of corrective therapy.

Reactogenicity events are AEs that are known to occur with this type of vaccine. Solicited reactogenicity is analyzed using the following grading scales:

This summary includes the following data:

- Cohort 1: Data are included for 20 children who were enrolled and vaccinated in Cohort 1. They received the first dose on 3 and 4 November 2006, the second dose on 4 - 6 December 2006, and the third dose on 7 – 9 January 2007.

- Cohort 2: Data are included for 40 children who were enrolled and vaccinated in Cohort 2. They received the first dose on 23 and 24 November 2006, the second dose on 26 - 28 December 2006, and the third dose on 24 and 26 January 2007

- Cohort 3: Data are included for 40 children who were enrolled and vaccinated in Cohort 3. They received the first dose on 13 - 15 December 2006, the second dose 17 - 19 January 2007, and the third dose on 9 -13 February 2007.

**Table 10 Toxicity grading scale for reactogenicity events**

| Adverse Event (AE) | Grade | Intensity Definition |
|---|---|---|
| Pain/tenderness at injection site | 0 | Absent |
| | 1 | Minor reaction to touch |
| | 2 | Cries/protests on touch |
| | 3 | Cries when limb is moved/spontaneously painful |
| Swelling at injection site | 0 | Absent |
| | 1 | < 5 mm |
| | 2 | 5-20 mm |
| | 3 | > 20 mm |
| Erythema at injection site | 0 | Absent |
| | 1 | < 5 mm |
| | 2 | 5-20 mm |
| | 3 | > 20 mm |
| Limitation of arm motion (Abduction at the shoulder) | 0 | None |
| | 1 | >90° but <120° |
| | 2 | >30° but ≤90° |
| | 3 | ≤30° |
| Fever | 0 | < 37.5°C |
| | 1 | 37.5-38.0°C |
| | 2 | 38.1-39.0°C |
| | 3 | > 39.0°C |
| Irritability/fussiness | 0 | Behavior as usual |
| | 1 | Crying more than usual/ no effect on normal activity |
| | 2 | Crying more than usual/ interferes with normal activity |
| | 3 | Crying that cannot be comforted/ prevents normal activity |
| Drowsiness | 0 | Behavior as usual |
| | 1 | Drowsiness easily tolerated |
| | 2 | Drowsiness that interferes with normal activity |
| | 3 | Drowsiness that prevents normal activity |
| Loss of appetite | 0 | Normal |
| | 1 | Eating less than usual/ no effect on normal activity |
| | 2 | Eating less than usual/ interferes with normal activity |
| | 3 | Not eating at all |
| Vomiting | 0 | Absent |
| | 1 | Occasional but able to eat/drink normal amounts |
| | 2 | Repeated with limited oral intake |
| | 3 | Continuous, unable to keep down liquids or solids |

### Summary of the safety data

A total of 100 participants were enrolled in the study and all immunizations have been administered. One hundred participants received the first immunization, 98 received second immunizations, and 91 participants received all three immunizations. Of the nine participants who did not receive all three immunizations, two were because of anemia on the scheduled day of immunization, five due to elevated ALT and/or documented hepatitis A or hepatitis B, one because of a systolic heart murmur on the day of immunization, and one because of parental refusal of blood draws. Two SAEs were reported by the data cutoff date; both events were considered SAEs based on criteria for Grade 4 laboratory abnormalities and were assessed to be not associated with the vaccine (Cohort 1 participant Grade 4 WBC day 37; Cohort 2 participant Grade 4 ALT day 37). Ninety-eight participants experienced 486 unsolicited adverse events, of which 31 were assessed to be

associated with the vaccine. Fifty-three percent of unsolicited adverse events were classified as “infections and infestations,” and 22% were classified as “respiratory, thoracic and mediastinal disorders.”

Systemic reactions were typically mild and most prevalent within two days following immunization (Figure 6). No severe systemic reactions were reported. Fever, the most common systemic reaction, was experienced by 40% of all participants. Over the three immunizations, the frequency of fevers increased with vaccine dose. In Cohort 1, 15% participants experienced fever, with 42.5% and 50% of participants in Cohort 2 and Cohort 3, respectively. Other systemic reactions were infrequent and mild. Four participants experienced loss of appetite with a maximum severity of mild and one participant experienced mild vomiting.

Local reactions were observed mostly within three days of immunization, with a peak occurring on the day following immunization (Figure 7). The most common local reaction, swelling, was experienced by 70% of Cohort 1, 80% of Cohort 2, and 90% of Cohort 3. Overall, 80% of the participants had maximum swelling of severe (swelling was graded “severe” based on exceeding 20 mm at its widest dimension). Pain and tenderness was observed in 70% of the participants. The severity of pain/tenderness decreased on subsequent doses. Erythema was observed in one participant with a maximum severity of severe (Cohort 3).

There were 91 post-immunization clinical laboratory values of Grade 1 of higher toxicity reported by 43 participants. Eleven participants in Cohort 1 (55%) had abnormal post-immunization values, 17 in Cohort 2 (43%), and 15 in Cohort 3 (38%). Eighteen participants were reported to have abnormal lymphocyte counts; maximum severity for any participant was Grade 1 toxicity and 23 participants had abnormal white blood cell count. Elevated ALT was observed in 11 participants with a maximum severity of Grade 4; most abnormal ALTs were found to be associated with Hepatitis A or Hepatitis B.

**Study status and demographics.** Fifteen children received the FMP2.1/AS02A at the 10 µg dose and 5 received rabies vaccine in Cohort 1. In Cohort 2, 30 children received the FMP2.1/AS02A at the 25 µg dose and 10 received rabies vaccine. In Cohort 3, 30 children received the FMP2.1/AS02A at 50 µg dose and 10 received the rabies vaccine. In each cohort, the children were randomized into 3 age strata within the cohort: 1-2 year olds, 3-4 year olds, and 5-6 year olds. All participants have submitted data through Day 90 (30 days after the third scheduled immunization). Of the 20 enrolled participants in Cohort 1, 18 have data submitted through Day 120 (7 days after the second immunization). One child in Cohort 3 discontinued study participation due to parental decision to withdraw and discontinue immunizations before the second immunization. Seven additional children have discontinued the immunization schedule because of other clinical conditions or laboratory abnormalities, but remain in the study for follow-up.

**Adverse events and serious adverse events.** Two serious adverse events (SAEs) were reported by the data cutoff. One experienced a Grade 4 elevation of WBC in the context of an acute malaria episode. The other was a Grade 4 elevated ALT with no clinical symptoms. (Individual laboratory values that meet Grade 4 criteria according to the protocol toxicity table are reported as SAEs, irrespective of presence or absence of associated clinical manifestations.)

Ninety-eight of the 100 participants have had a total of 486 unsolicited adverse events reported. Table 11 and Table 12 summarize these events by severity and relationship and number of events per participant. The maximum severity of unsolicited adverse events for each participant is reported by cohort in Table 12. Overall, 70 participants had maximum severity of mild, 25 had maximum severity of moderate, and 3 (1 from Cohort 1 and 2 from Cohort 2) had a maximum severity of severe. There were no reports of unsolicited adverse events that were both assessed as severe and associated.

**Figure 6 Maximum systemic reactogenicity by time after vaccination**

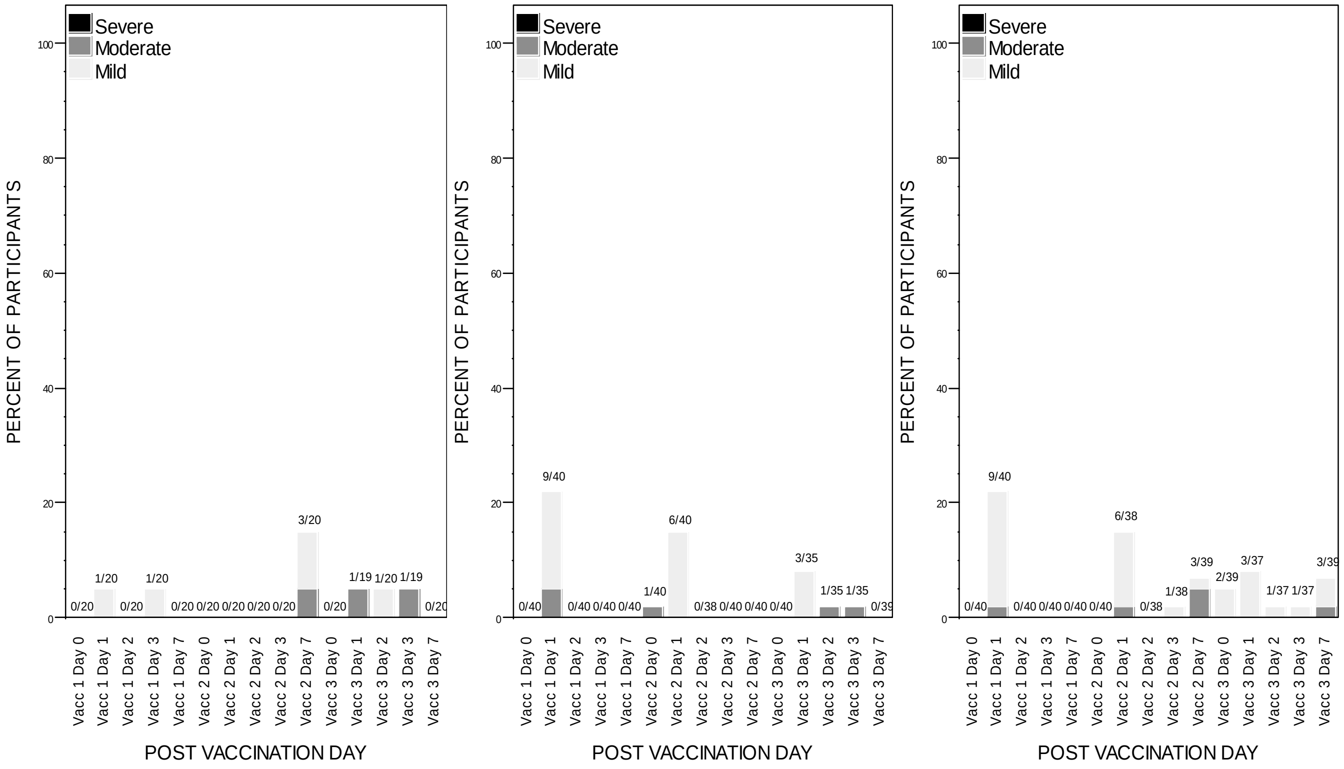


Cohort 1 Cohort 2 Cohort 3

**Table 11 Maximum severity of unsolicited adverse events**

| Cohort | No Adverse Events | Maximum Severity of Adverse Event | | |
|---|---|---|---|---|
| | | Mild | Moderate | Severe |
| 1 (N=20) | 0 | 9 | 10 | 1 |
| 2 (N=40) | 0 | 31 | 7 | 2 |
| 3 (N=40) | 2 | 30 | 8 | 0 |
| **TOTAL** | **2** | **70** | **25** | **3** |

**Table 12 Associated adverse events: Maximum severity by cohort**

| Cohort | No Associated Events | Maximum Severity of Associated Event | | |
|---|---|---|---|---|
| | | Mild | Moderate | Severe |
| 1 (N=20) | 15 | 5 | 0 | 0 |
| 2 (N=40) | 32 | 8 | 0 | 0 |
| 3 (N=40) | 30 | 7 | 3 | 0 |
| **TOTAL** | 77 | 20 | 3 | 0 |

In all, there were 23 participants with associated events: 5 participants in Cohort 1, all with a maximum of mild; 8 participants in Cohort 2, all with a maximum of mild; and, 10 participants in Cohort 3, 7 of whom had a maximum of mild and 3 moderate (one was classified as injection site pain and 2 were classified as asthenia). The moderate associated AEs were all Cohort 3 cases. Thirty-one events were assessed as associated with immunization, classified in the following MedDRA Preferred Term classification: diarrhoea (N=3), asthenia (N=3), injection site pain (N=2), pyrexia (N=17)

**Figure 7 Maximum local reactogenicity by time after vaccination**

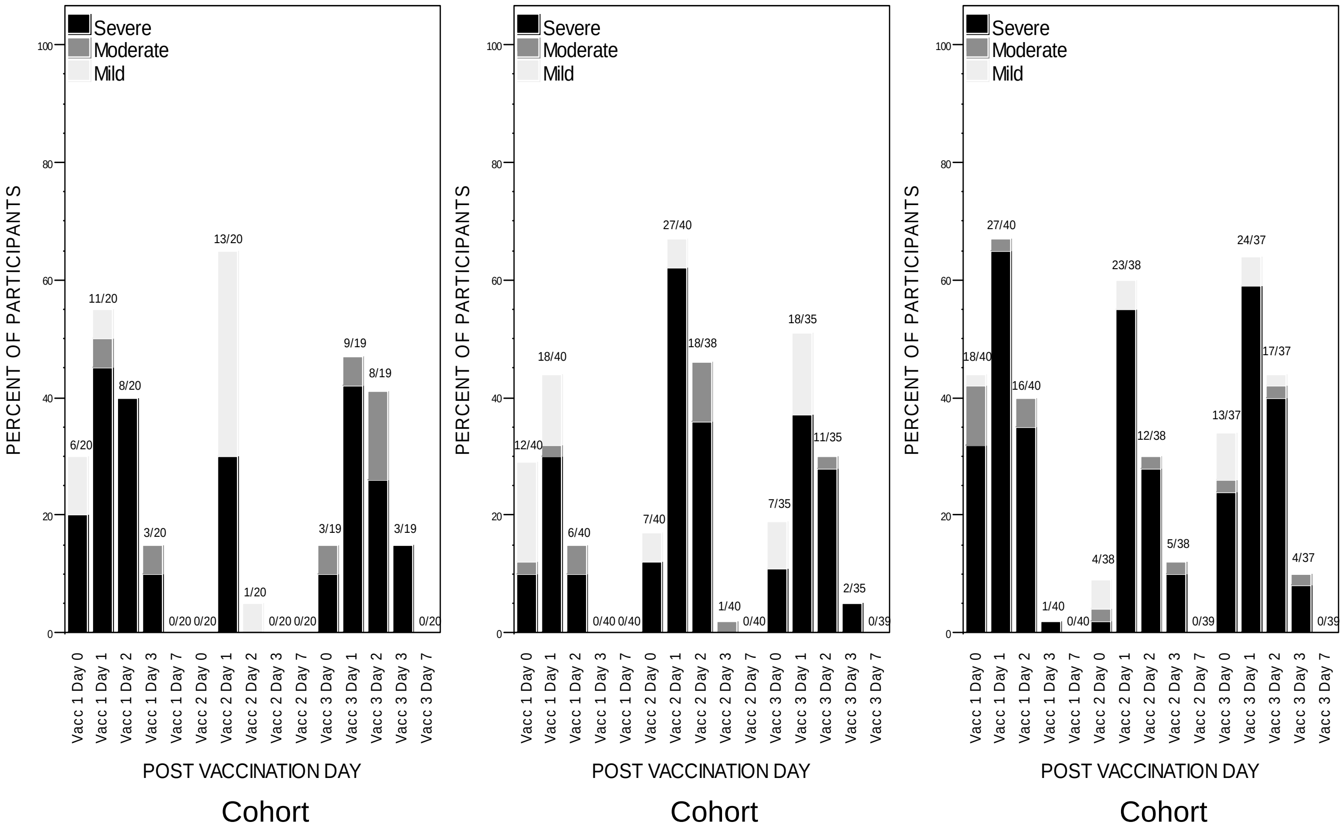

**Clinical laboratory evaluations.** The most frequently abnormal laboratory results of Grade 1 or greater severity were elevated ALT, abnormal WBC results and abnormal lymphocyte counts. Elevated ALT results of Grade 1 or greater occurred in 11/100 participants. Of those, 7 participants had abnormal ALTs with a maximum of Grade 2 or greater - 3 with a Grade 2 maximum, 3 with a Grade 3 maximum, and 1 with a maximum of Grade 4. Of the 7 cases of abnormal ALT, two were determined to be associated with hepatitis A infection, two with hepatitis B infection, and two remain under investigation at the time of this summary.

Abnormal WBC counts of Grade 1 or greater occurred in 23/100 participants. Abnormal lymphocyte counts of Grade 1 or greater occurred in 18/100 participants. Of these participants, 9 had maximum WBC abnormalities of Grade 2, one Cohort 2 participant had a maximum Grade 3 elevation of WBC and one Cohort 1 participant had a maximum Grade 4. All participants with lymphocyte count abnormalities had a maximum of Grade 1.

For the other laboratory parameters measured, no Grade 3 or 4 abnormalities were reported. The only other Grade 2 abnormality was a Cohort 3 participant with a hemoglobin value of 7.0 g/dL at Day 30. Four participants had Grade 1 creatinine abnormalities.

**Summary.** In general, this data from this Phase 1 study are consistent with the good safety record from previous studies of the vaccine in North American and Malian adults. The most common adverse events related to immunization are local reactions at the injection site. The vast majority of these reactions consist of local swelling, which is transient, usually unaccompanied by pain or other local symptoms, and not associated with any systemic symptoms. This swelling is classified as Grade 3 if it exceeds 20 mm at its widest dimension, but it does not cause any functional impairment and does not interfere with normal activity. The swelling is not noted to be of concern to parents. Other local and systemic unsolicited adverse events are consistent with the normal childhood diseases in this population. The study remains blinded, but even assuming a worst case scenario in which a preponderance of the reactogenicity occurred in the FMP2.1/AS02A group, all three dose levels of the vaccine are safe and well tolerated enough to proceed with efficacy testing. The DSMB has concurred with this assessment and recommended proceeding with the Phase 2 trial of 50 µg FMP2.1/0.5 mL AS02A.

### Clinical experience with the AS02A adjuvant

The adjuvant system AS02A, previously known as SBAS2, consists of an oil-in-water emulsion and phosphate buffered saline combined with two immunostimulants, monophosphoryl lipid A (MPL) and a saponin derivative known as Stimulon QS21 (a triterpene glycoside purified from the bark of *Quillaja saponaria)* (32-34). MPL is a detoxified, deacylated form of monophosphoryl lipid A, derived from the lipopolysaccharide (LPS) of *Salmonella minnesota*. LPS, and more specifically, its lipid A component, has long been known for its strong adjuvant effects; however, until recently, its high toxicity precluded its use in a vaccine formulation. Ribi et al. (35) showed that the monophosphorylated form of lipid A retains its adjuvant function and almost completely loses its endotoxin effects. Subsequently, the 3-deacylated form of MPL was shown to have a further decrease in its toxicity as tested in small animals, but retains its immunopotentiating effect (36). Several immunogenicity studies performed in mice, guinea pigs, monkeys, and humans have

shown that inclusion of 3D-MPL into a vaccine preparation potentiates both specific antibody and cellular immune responses (36-38). The term MPL in this protocol refers to the 3-deacylated form of the compound. To date, the bulk of the experience with this formulation in malaria vaccines has been in conjunction with the RTS,S antigen reviewed above.

**Table 13 Summary of clinical experience with the AS02A adjuvant in African subjects.**

| Vaccine | Location (reference if published) | Age (years) | Number of recipients of AS02A | Number of doses | Dose |
|---|---|---|---|---|---|
| RTS,S/AS02A | Gambia (39) | 18-45 | 20 | 60 | 0.5mL[1] |
| RTS,S/AS02A | Gambia (40) | 18-45 | 153 | 435 | 0.5mL[1] |
| RTS,S/AS02A | Gambia (41) | 6-11<br>6-11<br>6-11 | 20<br>20<br>20 | 60<br>59<br>60 | 0.5mL[1]<br>0.25mL[2]<br>0.1mL[3] |
| RTS,S/AS02A | Gambia (41) | 1-5<br>1-5<br>1-5 | 30<br>30<br>30 | 89<br>89<br>89 | 0.5mL[1]<br>0.25mL[2]<br>0.1mL[3] |
| RTS,S/AS02A | Mozambique | 1-4 | 30 | 84 | 0.25mL[2] |
| RTS,S/AS02A | Mozambique (9;10) | 1-4 | 803 | 2326 | 0.25mL[2] |
| RTS.S/AS02A | Kenya | 18-45 | 20 | 60 | 0.5mL[1] |
| RTS,S/AS02A | Kenya | 18-35 | 85 | ~240 | 0.5mL[1] |
| FMP1/AS02A | Kenya (42) | 18-55 | 20 | 59 | 0.5mL[4] |
| FMP1/AS02A | Mali | 18-55 | 20 | 60 | 0.5mL[4] |
| FMP1/AS02A | Kenya | 1-4 | 90 | 84<br>88<br>88 | 0.5 mL[4]<br>0.25 mL[5]<br>0.10 mL[6] |
| FMP1/AS02A | Kenya | 1-4 | 200 | ~600 | 0.5 mL[4] |
| FMP2.1/AS02A | Mali | 18-55 | 40 | 19<br>20 | 0.5 mL[7]<br>0.25 mL[8] |
| FMP2.1/AS02A | Mali | 1-6 | 75 | pending | 0.5 mL[7]<br>0.25 mL[8]<br>0.10 mL[9] |

[1] 0.5mL = 50μg RTS,S + 0.5mL AS02A
[2] 0.25mL = 25μg RTS,S + 0.25mL AS02A
[3] 0.1mL = 10μg RTS,S + 0.1mL AS02A
[4] 0.5mL = 50μg FMP1 + 0.5mL AS02A
[5] 0.25mL = 25μg FMP1 + 0.25mL AS02A
[6] 0.10 mL = 10 μg FMP1 + 0.10 mL AS02A
[7] 0.5 mL = 50 μg FMP2.1 + 0.5 mL AS02A
[8] 0.25 mL = 25 μg FMP2.1 + 0.25 mL AS02A
[9] 0.1 mL = 10 μg FMP2.1 + 0.1 mL AS02A
FMP1 – MSP-1-based vaccine; FMP2.1 – AMA-1 based vaccine, RTS,S – CSP-based vaccine; N/A – Not available

These studies, with a large cumulative experience in both malaria-naïve and malaria-experienced adults and children, have demonstrated that AS02A has a good safety profile when used with the RTS,S vaccine, and suggest that it is similarly safe and well-tolerated when formulated with the FMP1 and FMP2.1 blood-stage vaccines.

### Summary of clinical experience with FMP2.1/AS02A

On the basis of preclinical and clinical studies performed to date with FMP2.1/AS02A, there are no risks of particular severity or seriousness anticipated with the formulation.

Extensive clinical data exist for AS02A administered with FMP2.1, additional malarial antigens, hepatitis B surface antigen (HBsAg), and HIV antigens. In general the AS02A formulated vaccines are safe and well tolerated when administered to adults, both naïve and malaria experienced, and in malaria experienced children. The most common side effect related to immunization is pain at the injection site. Redness and swelling are less frequent. The most frequent general side effects are headache and malaise, usually mild to moderate and short in duration.

Although the three previous clinical studies with FMP2.1/AS02A have resulted in frequent grade 3 reactions, these reactions were short-lived, with most resolving in less than 48 hours after vaccination. In malaria-exposed Malian adults followed for a year, there were more unsolicited adverse events than in North American adults, as expected. Malian adults also experienced more local injection site swelling, which was short-lived and usually not symptomatic. In Malian children increasing dose levels of the vaccine were associated with increased rates of local swelling on the day after immunization (Day 1). This swelling was usually unaccompanied by pain or other symptoms, and resolved in most cases by Day 2 and in all cases by Day 3.

Based on the experience with the FMP2.1 antigen and the AS02A adjuvant, other than standard precautions for idiosyncratic or allergic reactions, a need for special monitoring is not anticipated in the present trial.

### Justification of 0-, 1-, 2-month schedule

The overall testing program of FMP2.1 aims at developing a vaccine for administration with routine childhood vaccines. With this goal in mind, the present study uses a 0-, 1-, and 2-month immunization schedule anticipating that this schedule will be amenable to eventual incorporation into the Expanded Programme on Immunization of the WHO.

### Justification of the vaccine dosing regimen and next steps for the vaccine

The clinical development of the candidate malaria vaccine FMP2.1/AS02A as a vaccine to combat malaria in the developing world is progressing in five distinct steps: 1) a Phase 1 dose-escalation trial in malaria naïve adults (completed at WRAIR); 2) a Phase 1 dose-escalation trial in malaria-experienced adults (completed in Mali); 3) a Phase 2a safety, immunogenicity and efficacy trial in malaria naïve adults at WRAIR (ongoing); 4) a Phase 1 dose-escalation study in young children in

Mali (ongoing with final immunizations in mid-February 2007); and 4) an efficacy trial of the selected dose in the target pediatric population (the present study).

The overall goal of this Phase 2 study in malaria-experienced children is to provide proof of principle with demonstration of measurable efficacy that justifies further development of this vaccine alone or in a multi-stage multi-antigen combination vaccine. The FMP2.1/AS02A vaccine is intended to induce antibodies active against *Plasmodium falciparum* blood stage parasites. There was an apparent dose-dependent increase in antibody responses between the 25 and 50 μg dose levels in the adult Phase 1 trial of this vaccine in Mali, suggesting that the 50 μg dose level may be more likely to show measurable efficacy in a Phase 2 trial. An apparent dose-dependent increased frequency of local solicited adverse events was also noted in both the adult and pediatric Phase 1 trials in Mali, but these local adverse events consisted almost entirely of swelling that caused no functional impairment, was generally not associated with pain or other symptoms, and was not unduly concerning to parents or clinicians. The overall incidence of local reactions is similar to that of other licensed vaccines. Considering the potential value of an efficacious malaria vaccine, the risk-to-benefit ratios, both for individual participants and for the malaria-afflicted population as a whole, favor testing of the 50 μg/0.5 mL dose of FMP2.1/AS02A in the first test of efficacy for this vaccine.

## Comparator vaccine

### Rationale for rabies comparator vaccine

Having a comparator vaccine as opposed to using a placebo is useful in efficacy trials conducted in malaria-endemic areas, since background immunity and natural exposure to malaria make it difficult to interpret immunogenicity data and establish correlates of protective efficacy. In this setting, rising titers of antibody to AMA-1 could be due to immunization or to natural exposure or both. The use of a control group will permit comparison of immune responses and permit clearer interpretation of immunogenicity and efficacy results. While a placebo control group would accomplish this same end, using a vaccine that is beneficial to the subjects further increases the benefit-to-risk ratio. We have chosen to use rabies vaccine as the comparator for several reasons: 1) the dosing schedule is compatible with that of FMP2.1; 2) to be able to compare results directly with recent studies of the FMP1/AS02A and FMP2.1/AS02A vaccines in Mali and Kenya that used rabies vaccine as a comparator; and 3) the available evidence supports a benefit for participants who receive rabies vaccine. A placebo would offer no benefit.

Rabies prevalence in Mali is not known but available data from the Ministry of Health's Division of Epidemiology suggest that the rabies burden is high. Approximately 1,500 dog bites were reported to public health officials in Bamako, the capital of Mali, from 1996 through 1999. The vast majority of dogs are unvaccinated and in most cases the status of rabies infection is unknown. The heads of 124 dogs were examined for evidence of rabies infection: rabies infection was found in 34 (27%); 7 cases were negative and there were no reported results for 74 cases (60%). These data allow us to estimate that 25-30% or more of dogs that bite humans may be carrying and potentially transmitting rabies infection. In the Bandiagara district health center, approximately one case of human rabies is reported per year. This is likely a considerable underestimate of the true incidence of rabies cases

given the general population's reliance on traditional healers and the relatively low utilization of the district health center. Of note, three dog bites were reported among the 40 study subjects during a Phase 1 trial of malaria vaccine FMP1/AS02A in Kenya that used the rabies vaccine as a comparator (43). Because of the potential benefit to Bandiagara residents, participants in the three previous malaria vaccine trials who received malaria vaccine were offered rabies vaccine at the end of the trials.

When the RabAvert/Rabipur® rabies vaccine is administered according to the recommended immunization schedule (days 0, 7, 21), nearly 100% of subjects attain a protective titer. In two studies carried out in the US in 101 subjects, protective antibody titers >0.5 IU/mL were obtained by day 28 in all subjects. In studies carried out in Thailand in 22 subjects, and in Croatia in 25 subjects, antibody titers of >0.5 IU/mL were obtained by day 14 (injections on days 0, 7, 21) in all subjects (44-47).

High antibody titers have also been demonstrated with off-label immunization with rabies vaccines. Among participants in England, Germany, France and Belgium who received two immunizations one month apart, nearly 100% of the participants developed specific antibody and the geometric mean titer for the group was 10 IU (48-51). The proposed immunization schedule of 0, 1, and 2 months is therefore expected to be highly successful in conferring protective immunity against rabies among the control participants.

### Safety of RabAvert/Rabipur® rabies vaccine

The rabies vaccine is sold under the name RabAvert for use in the U.S.under FDA approval and as Rabipur outside the U.S. under WHO approval. The two names refer to the same product, manufactured and sold by Chiron Vaccines. Local and/or mild systemic reactions may occur after injection of RabAvert/Rabipur rabies vaccine but these are usually transient and do not contraindicate continuing immunization. Local reactions such as induration, swelling and reddening have been reported more often than systemic reactions. In a comparative trial in normal volunteers, the most commonly reported adverse reaction was pain at the injection site, reported in 34% of 19 volunteers who received RabAvert/Rabipur. Localized lymphadenopathy was reported in about 15%. The most common systemic reactions were malaise (15%), headache (10%), and dizziness (15%). In a recent study in the USA (4), 83 subjects received RabAvert/Rabipur. The most common adverse reaction was pain at the injection site in 84%. The most common systemic reactions were headache (52%), myalgia (53%) and malaise (20%). None of the adverse events was serious; almost all were of mild or moderate intensity.

Uncommonly observed adverse events include temperatures above 38°C (100°F), swollen lymph nodes, and gastrointestinal complaints. In rare cases, patients have experienced severe headache, fatigue, circulatory reactions, sweating, chills, monoarthritis and allergic reactions; transient paresthesias and one case of suspected urticaria pigmentosa have also been reported. Human serum albumin (HSA) is present in RabAvert/Rabipur at concentrations less than 0.3 mg/dose. No type III hypersensitivity reactions have been observed with RabAvert/Rabipur. Serious systemic anaphylactic reactions or neuroparalytic events have been reported in association with RabAvert/Rabipur administration. Against a background of 11.8 million doses distributed world-

wide, ten cases of encephalitis (one death) or meningitis, seven cases of transient paralysis including two cases of Guillain-Barré Syndrome, one case of myelitis, one case of retrobulbar neuritis, and two cases of suspected multiple sclerosis have been temporally associated with the use of RabAvert/Rabipur. Also, two cases of anaphylactic shock have been reported.

The RabAvert/Rabipur and Imovax rabies vaccines have been well-tolerated in the three previous malaria vaccine trials conducted in Bandiagara.

## 1.5 Potential risks and benefits

### Potential risks

### Vaccination

Risks associated with both FMP2.1/AS02A and rabies vaccinations include local inflammatory reactions to the injected product, such as injection site pain and swelling. Systemic effects generally associated with vaccines may include flu-like syndrome, fever, chills, nausea/GI symptoms, headache, malaise, myalgia and arthralgia. While rare, allergic reactions, including life-threatening anaphylaxis, are associated with many vaccine preparations and must therefore be considered as a potential risk in this study. Risks associated with drawing blood include fainting, infection and bruising.

Free medical treatment will be provided to enrolled participants during the active immunization phase and the surveillance period, at a level that meets the local Malian standards of medical diagnosis and treatment. Medical care for ailments not related to vaccination will not extend beyond the study period. Medical care for ailments related to vaccination will extend, at minimum, until the condition has resolved or stabilized.

### Known potential benefits

Participants may not receive any direct benefit from the experimental vaccine. However, they will receive outpatient follow-up medical care at the BMP research clinic. Routine and emergency health care including hospitalization will be provided by study clinicians at the Bandiagara District Hospital, in collaboration with District Hospital physicians. Free initial medical evaluations by study clinicians and basic treatments will also be provided to family members of participants and to other non-study participants who seek care at the research clinic, with referral to the District Hospital or other sources of care as appropriate. During the conduct of the study participants randomized to receive rabies immunization will benefit from this intervention due to the reduced risk of rabies, which is present in this region. At the end of the study all participants and their parents/guardians will be informed of the vaccine they received. Participants randomized to the FMP2.1/AS02A vaccine will be offered rabies immunization at that time. These immunizations will be given at the recommended schedule of 0, 7 and 21 days.

The benefits to the community include an overall improvement of the level of medical care provided at the District Hospital resulting from the ongoing presence of the research team, including high-quality malaria diagnosis, case management of severe malaria and other conditions, and increased capacity to manage severe anemia and other complications of severe malaria. Because of heightened awareness in the community of severe malaria has resulted in more frequent and earlier treatment-seeking for severe malaria and lower case fatality rates. If this research is successful and a safe and effective malaria vaccine is developed and licensed, the community of Bandiagara and the rest of the malaria-endemic world will benefit.

### Risks to study personnel

The main risks to study personnel are from accidental exposure to blood and body-fluid borne infections. SOPs for staff safety are used in clinical and laboratory areas, including sharps management, hazardous waste management, etc. Universal precautions are used for handling all body fluids.

# 2 OBJECTIVES

## 2.1 Study objectives

### Overall study objective

Determine the efficacy of the FMP2.1/AS02A vaccine against clinical malaria and extend the safety profile of this vaccine.

### Primary specific objective

- Determine the efficacy of FMP2.1/AS02A in children aged 1-6 years against first clinical malaria episodes (axillary temperature of $\geq$ 37.5°C and parasitemia of $\geq$2500/mm$^3$) occurring between randomization and six months after the assigned date of the third immunization
- Assess the safety of the vaccine in children aged 1-6 years

### Secondary specific objectives

- To describe the dynamics of anti-AMA-1 antibody responses in recipients of the malaria vaccine compared to natural responses in the control group
- To determine whether serum anti-AMA-1 IgG titer by ELISA one month after the third immunization correlates with protection against clinical malaria episode
- To measure allele-specific efficacy against parasites with AMA-1 genotypes homologous to and heterologous to the 3D7 clone of *P. falciparum*
- To determine vaccine efficacy against clinical malaria episodes occurring between randomization and six months after the assigned date of the third immunization
- If efficacy is observed based on the primary endpoint, to determine vaccine efficacy against first clinical malaria episode and all clinical episodes (using increasing parasitemia thresholds) occurring during two years after randomization

### Exploratory objectives

- To determine vaccine efficacy of FMP2.1/AS02A in children aged 1-6 years against clinical malaria episodes using increasingly specific definitions of clinical episodes (parasitemia thresholds of any parasitemia, 100, 1000, 2500, 5000, 10,000, 20,000, 50,000 and 100,000/mm$^3$; and any symptoms consistent with malaria, with and without measured fever) occurring between randomization and six months after the assigned date of the third immunization

- To measure the age-specific vaccine efficacy in children aged 1-2, 3-4 and 5-6 years
- To determine whether three doses of vaccine significantly protect against malaria infection, as measured by the prevalence and density of parasitemia by comparing malaria and control vaccine recipients with regard to rates of asexual *P. falciparum* parasite density and gametocyte counts, as determined at active surveillance time points.
- To determine vaccine efficacy against the incidence of anemia
- To determine vaccine efficacy against the incidence of severe malaria
- To measure cellular immune responses to FMP2.1 at baseline and after immunization and assess the association of these responses with vaccine efficacy
- To determine the specificity of antibodies and cell mediated immune responses to diverse AMA-1 genotypes in addition to 3D7, by measuring by ELISA, growth inhibition assay (GIA), immune fluorescence assay (IFA) and cell-mediated immune (CMI) responses to parasites with typed AMA-1

## 2.2 Study outcome measures

### Primary outcome measures

1. Time to first clinical malaria episode with significant parasitemia (2500/mm$^3$) and temperature of $\geq$ 37.5$^o$C occurring between randomization and six months after the assigned date of the third immunization
2. Occurrence of solicited adverse events after each vaccination during a seven-day surveillance period (day of vaccination and days 1, 2, 3 and 7 after vaccination)
3. Occurrence of unsolicited symptoms after each vaccination during a 30-day surveillance period (day of vaccination and 30 subsequent days)
4. Occurrence of serious adverse events throughout the study period

### Secondary outcome measures

1. Titers of anti-FMP2.1 antibody at each time point where serology samples are analyzed, measured by ELISA
2. Time to first clinical malaria episode with parasites with AMA-1 genotype homologous to the 3D7 strain of *P. falciparum* with respect to entire AMA-1 sequence and with respect to key amino acid residues
3. Incidence density of clinical malaria episode occurring between randomization and six months after the assigned date of the third immunization

4. If efficacy is measured during eight months after the first immunization, time to first clinical malaria episode and all incidence density of clinical episodes (using increasing parasitemia thresholds) occurring during two years after randomization

# 3 STUDY DESIGN

## 3.1 Overview

- Randomized, controlled, participant-blind, observer-blind, Phase 2 study
- One study center
- 400 healthy male and female children aged 1-6 years residing in the rural town of Bandiagara, Mali
- Safety oversight from a DSMB and LMM
- Study monitoring delegated to DMID or DMID designate
- Screening will be done within 45 days prior to the first immunization
- Randomization 1:1 in three age strata to immunization with 50 μg of FMP2.1 adjuvanted with 0.5 mL of AS02A (n=200) or rabies vaccine (n=200)
- Immunization schedule will be on study days 0, 30 +/- 7, and 60 +/- 7
- Route of immunization will be IM in alternating deltoid muscles
- Source documents will be derived from the electronic CRFs. They will contain all the protocol required information including clinical laboratory test results and adverse event medical records. The information in the source document will then be entered directly into the Internet data system.
- Study duration will be approximately 26 months per participant
- Direct observation for 60 minutes after immunization
- Seven-day surveillance after each immunization (day of vaccination and study days 1, 2, 3 and 7) for solicited adverse events
- 30-day surveillance after each immunization for unsolicited adverse events
- Passive case detection of clinical malaria episodes and adverse events including SAEs
- Active surveillance for asymptomatic malaria infection and adverse events including SAEs

- Follow-up of SAEs until resolution or stability; follow-up of AEs until resolution or stability or until the end of the trial

- Beginning Study Day 120, participants will be visited and assessed by local guides at home at monthly intervals and return to clinic nine, 12, 18 and 24 months after randomization for safety surveillance.

- The primary unblinded analysis will be conducted for all primary and secondary endpoints at a data-lock-point eight months after randomization, after which the Primary Study Report will be produced.

- The study will then continue in a single-blind fashion (participants will not be informed of study assignments until the end of the study, although investigators will have access to unblinded data). Data gathered after the scheduled visit at study day 240 will be reported in an addendum report.

- At the end of the study participants and their parents/guardians will be informed which vaccine they received and those who received malaria vaccine will be offered immunization with the rabies vaccine.

# 4 STUDY ENROLLMENT AND WITHDRAWAL

## 4.1 Site description

Participants for this study will be drawn from the population of healthy children aged 1-6 years residing in the town of Bandiagara, which has been the site of MRTC malaria studies since 1993. Bandiagara (pop. 13,364), is a rural town 700 km northeast of Bamako, the capital of Mali. In a census conducted in 1999, the predominant ethnic group is Dogon (64%) followed by Peuhl (12%), Bambara (6%) and 16 other ethnic groups. About 85% of residents reported their religion to be Islam, and about 8% reported belonging to various Christian denominations. Animist beliefs and practices were also common. The most common occupations among men were in agriculture (30%) with 21% in the commercial sector, mostly self-employed as vendors of various goods, and the rest reported a wide variety of occupations including 6% who were government workers and smaller numbers of teachers, health workers, traditional healers and practitioners of spiritual and magical arts. Nearly 80% of women reported their occupation as housewife, but it is evident to the casual observer that a large proportion of the women in Bandiagara are also working in agriculture and/or as producers and sellers of various goods. Forty-six percent of adults had received no formal schooling, and only 14% reported themselves to be literate. However, 86% of children of the age to be in primary school (7-11 years) were attending school. Seventy-five percent of marriages were reported to be monogamous and 25% were polygamous with 2-4 wives.

The annual per capita income for Mali as a whole is estimated to be $600. However, this figure is based on large segments of the population who live in remote rural areas with virtually no cash economy, and per capita income in Bandiagara is certain to be higher than this. Income data specifically for Bandiagara are not available, but it is a relatively affluent town by Malian standards, with a lively market economy and a significant tourist trade. Most homes are single story structures made of mud brick and/or concrete organized into compounds with several small dwellings surrounding a central courtyard. A main road runs through the length of the town, which extends about 2 km from end to end. All homes of potential participants in this trial are within walking distance of the BMP research clinic.

From 1998 to 2006 Bandiagara was the site of an NIAID-supported program for developing a site for testing malaria vaccines, known as the Bandiagara Malaria Project (BMP). Support for the BMP from 2006 through 2010 is provided by a cooperative agreement from NIAID awarded to the Universities of Maryland and Bamako, with supplemental support from USAID and the US Department of Defense, to conduct Phase 1 and 2 pediatric trials of the FMP2.1/AS02A malaria vaccine. The BMP has completed censuses of Bandiagara, established a laboratory with the capability of preserving sera, peripheral blood mononuclear cells (PBMCs), live parasites and DNA; a clinical research center with a GCP-compliant clinical laboratory; and a severe malaria pediatric inpatient unit at the Bandiagara District Hospital in partnership with local healthcare workers. Thousands of children have been included in malaria research studies and treated for

uncomplicated and severe malaria (5;6;52-54). Loss to follow-up has consistently been less than 7%.

The BMP research clinic is on the campus of the Bandiagara District Hospital, where two buildings were renovated in 2003 for the first of two NIAID-supported malaria vaccine clinical trials that have been conducted. The clinical research facility includes an air-conditioned clinical laboratory and vaccine storage preparation room, six private consultation rooms, a procedure room, covered waiting area, two vaccination rooms, a resuscitation suite, an observation room, and storage and administrative space. Locked cabinets with restricted access are used for data storage.

The BMP team has been managing severe malaria at this site since 1998, providing 24-hour inpatient care. In addition to the present capacity for 24-hour nursing care and physician coverage, and laboratory tests for hematological and biochemistry parameters, new and upgraded pediatric inpatient care facilities were established for the Phase 1 pediatric trial in 2006. These facilities include the capacity for blood transfusion and oxygen supplementation. Children requiring X-rays will be transported to the District Hospital in Mopti, 60 km from the site on a paved road. Children requiring diagnostic or therapeutic interventions not available locally will be transported to the National Pediatric Hospital Gabriel Toure in Bamako.

The BMP team has been trained to conduct GCP-compliant studies, using source documents, written informed consent and standardized case report forms. The site has been monitored several times by DMID's contractor PPD, Inc., the US Army, and GSK as well as by the World Health Organization. The BMP team has established a strong trust and rapport with the community, which is very accepting of conducting malaria vaccine trials there.

The site is connected to the MRTC central laboratory in Bamako via a VSAT system, which allows high-speed communication link with Bamako, US partner institutions and the Internet. For the pediatric Phase 1 trial of FMP2.1/AS02A, data are entered in real time and uploaded daily via the internet to a central database in Rockville, MD, allowing ongoing data checking and safety monitoring. Back-up Internet service for E-mail and data uploading is available locally.

### Availability of medical and preventive care

In Mali citizens are encouraged to contribute minimally for medical care under the cost recovery principle. In Bandiagara, medical care is provided through the Bandiagara community-based health care center and the district hospital that hosts the BMP research team. For acute illness the medical consultation fee is CFA500 (US$1). Essential drugs are available at subsidized cost, and drugs for chronic diseases such as tuberculosis and AIDS are provided free of charge. Preventive care including vaccination against EPI target diseases and antenatal care are provided free of charge.

### Insecticide treated nets

Insecticide treated bed nets (ITN) are distributed in Bandiagara through the EPI program, which in 2006 distributed 7,321 ITNs to mothers of children completing the EPI immunization series in Bandiagara. With additional support from the Global Fund for Malaria, TB and HIV and the U.S.

President's Malaria Initiative, the National Malaria Control Program is planning to scale up ITN distribution in Mali. The senior Malian investigators, who advise the National Malaria Control Program, will ensure that Bandiagara is included in the early stages of this scale-up.

## 4.2 Subject inclusion criteria

- Age 1-6 years inclusive at the time of screening
- Residing in Bandiagara town
- Appear to be in generally good health based on clinical and laboratory investigation
- Separate written informed consent obtained from the parent/guardian before screening and study start, respectively
- Available to participate in follow-up for the duration of study (26 months)

## 4.3 Subject exclusion criteria

- Previous vaccination with an investigational vaccine or a rabies vaccine
- Use of a investigational or non-registered drug or vaccine other than the study vaccine(s) within 30 days preceding the first study immunization, or planned use up to 30 days after the third immunization
- Chronic administration (defined as more than 14 days) of immunosuppressants or other immune-modifying drugs within six months prior to the first immunization. This exclusion includes any dose level of oral steroids or inhaled steroids, but not topical steroids
- Confirmed or suspected immunosuppressive or immunodeficient condition, including human immunodeficiency virus (HIV) infection
- Confirmed or suspected autoimmune disease
- History of allergic reactions or anaphylaxis to immunizations or to any vaccine component
- History of serious allergic reactions to any substance, requiring hospitalization or emergent medical care
- History of allergy to tetracycline, doxycycline, nickel, Imidazole, eggs, neomycin, chlortetracycline or amphotericin B
- History of splenectomy

- Laboratory evidence of liver disease (alanine aminotransferase [ALT] greater than the upper limit of normal of the testing laboratory = 49.6 U/L).

- Laboratory evidence of renal disease (serum or plasma creatinine greater than 62 μmol/L), or more than trace protein or blood on urine dipstick testing).

- Laboratory evidence of hematologic disease (absolute leukocyte count <5,300/mm$^3$ or >15,300/mm$^3$, absolute lymphocyte count <2,300mm$^3$, platelet count <133,000/mm$^3$, or hemoglobin <9.0 g/dL).

- Hepatitis B surface antigen positive

- Chronic skin condition that could interfere with vaccine site reactogenicity assessment

- Administration of immunoglobulins and/or any blood products within the three months preceding the first study immunization or planned administration during the study period.

- Simultaneous participation in any other interventional clinical trial

- Acute or chronic pulmonary, cardiovascular, hepatic (including hepatomegaly), renal or neurological condition, severe malnutrition, or any other clinical findings that in the opinion of the PI may increase the risk of participating in the study

- Other condition that in the opinion of the PI would jeopardize the safety or rights of a participant in the trial or would render the participant unable to comply with the protocol

### Rationale for using clinical assessment of immunosuppression

We will not test for HIV routinely at the time of screening. HIV seroprevalence is 1.7% in Mali, one of the lowest rates in sub-Saharan Africa. Although no serosurveys have been done in Bandiagara, this site is in a remote rural area and almost certainly has a lower prevalence rate than the average for Mali. After working at this site for the past nine years, we have only very rarely encountered young adults, and virtually no young children, with illnesses that raised clinical suspicion of an immunosuppressive disease. In addition, the age distribution of HIV infection in Africa is bimodal, with most infection detected in young infants infected through maternal-to-child transmission or in sexually active teens and adults. Infection in children aged 1-6 years is almost certainly extremely rare in this setting. Therefore, testing for HIV would likely yield few, if any, cases of HIV in this study.

This study is intended to produce comparable results to other Phase 1 and 2 trials of recombinant protein blood stage malaria vaccines adjuvanted with AS02A, including recent and ongoing trials conducted in Kenya and Mali. In these studies, a similar approach was taken to exclude persons with clinical evidence of immunosuppressive disease but not test for asymptomatic HIV infection, based on the rationale that it is necessary to assess the safety and immunogenicity of this vaccine in generally healthy persons who are representative of the population from which they are drawn.

Based on this rationale, a WHO expert panel has recommended that HIV infection not be an exclusion criterion for malaria vaccine trials. In Kenya, where rates of HIV infection are much higher, this general population includes many persons living with HIV and a study that excluded them would be of less value. Eventually, it will be necessary to demonstrate the safety and immunogenicity in persons living with HIV for any malaria vaccine to be employed in Africa, but because of the low rates of HIV infection in Mali these studies will have to be conducted elsewhere. Meaningful subanalyses of HIV-infected and uninfected vaccinees are not possible in this trial.

Voluntary counselling and testing for HIV by rapid test is now available at the Bandiagara District Hospital, and antiretroviral treatment is available at the nearby Mopti District Hospital. In the event that HIV infection is suspected at the time of screening or later in the trial, participants and their parents/guardians will be referred to these sources of HIV testing and treatment.

## 4.4 Treatment assignment procedures

### Randomization procedures

Four hundred participants will be randomized to receive 50 μg of FMP2.1 adjuvanted with 0.5 mL of AS02A (n=200) or rabies vaccine (n=200) in the order they are enrolled with stratification for age by two-year increments (1-2 years, 3-4 years, 5-6 years) but without stratification for gender. Treatments will be assigned to participant ID numbers in randomized blocks of a size that will not be stated in this protocol to avoid compromising masking, per DMID guidelines. Randomization to either of the two vaccines will be done using a computer-generated randomization list. The randomization list will contain sequential codes linking a participant ID number to a vaccine assignment. Participant ID numbers will be assigned to participants of each cohort in the order in which they are enrolled in the trial.

### Masking procedures

Measures will be taken to keep participants, their parents/guardians and clinical investigators (including the PI) and all other staff involved in measuring study outcomes blinded to treatment allocation. Masking procedures are described in SOPs for randomization and vaccine preparation and administration. The FMP2.1/AS02A vaccine will have an opaque milky white appearance. The comparator rabies vaccine will appear as a clear to slightly opaque, colorless suspension after reconstitution. Therefore, blinding of the individuals preparing the study vaccine ("study pharmacists") will not be possible. Since the test article and comparison vaccines can be distinguished by appearance, the vaccine preparation area and the immunizing area will be physically separated. The two study pharmacists are both experienced pharmacists, and they will be dedicated exclusively to vaccine handling and preparation.

Immunizations will be carried out simultaneously in dedicated vaccination rooms adjacent to the vaccine preparation room and connected by small pass-through sliding doors. Despite the fact that the volumes of the study vaccine preparations are different, every attempt will be made to maintain blinding. The syringe barrels will be covered with opaque tape. Vaccinators will be physicians who

are not involved with surveillance activities, so that even if they realize which vaccine they are injecting, they will not be involved in the assessment of adverse events following vaccination. Each participant will be vaccinated in a closed room out of view of anyone other than the vaccinators, so that each parent sees only the syringe her child is injected with and never sees other participants being injected. The parents will not be told that the vaccines vary with respect to volume.

The Statistician at the EMMES Corporation in the US, the Local Medical Monitor (LMM) and USAMMDA will have the randomization list. The LMM will be provided sealed code-break envelopes and may unblind herself to the vaccine allocation of individual study participants if deemed urgently necessary for medical and/or ethical reasons (for example, in the case of a participant in urgent need of rabies immunization). USAMMDA will also be provided a sealed copy which will be held by the Division of Medical Affairs. Access to unsealed copies of the randomization list will be limited exclusively to the study pharmacists and the EMMES Corporation statistician. These individuals will be unblinded and will not be involved in study participants' further evaluation.

### Reasons for withdrawal

The following criteria will be checked at each visit. If any become applicable during the study, the subject will not be required to discontinue the study, but a separate immunogenicity analysis may be done that excludes these individuals.

- Use of any investigational drug or vaccine other than the study vaccine(s) during the study period.
- Chronic administration (defined as more than 14 days) of any dose level of immunosuppressants or other immune-modifying drugs during the study period and chronic daily use of inhaled steroids. Intermittent use of inhaled and topical steroids is allowed.
- Administration of a vaccine not foreseen by the study protocol during the period starting from 30 days before the first study immunization and ending 30 days after the third immunization.
- Administration of immunoglobulins and/or any blood products up to 30 days after the last study immunization.
- Unresolved laboratory abnormalities. Transient laboratory abnormalities that have been documented to have returned to normal range prior to immunization may not necessitate withdrawal, depending on the judgment of the PI in consultation with the LMM.

The following criteria will be checked prior to each immunization and are contraindications to further immunization. However, the study participants and their parents/guardians will be encouraged to continue to participate in the surveillance schedule for safety evaluation.

- Systemic hypersensitivity reaction following administration of the study vaccine. Severe (i.e., Grade III) local reactions will be evaluated to determine whether or not further study immunizations should be administered.

Subjects may also withdraw voluntarily from receiving the study intervention or from continuing study follow-up upon request for any reason.

### Handling of withdrawals

Every effort will be made to collect safety data on any subject discontinued from receipt of additional vaccinations because of an AE or SAE by continuing the safety follow-up procedures. If voluntary withdrawal from receipt of additional vaccinations occurs, the subject will be asked to continue scheduled evaluations and be given appropriate care under medical supervision until the symptoms of any AE resolve or the subject's condition becomes stable. If possible, subjects who leave the study area will be traced and visited by clinical investigators to collect safety follow-up data.

### Termination of study

The trial may be terminated by USAMMDA, or suspended by DMID, or the PI due to development of serious laboratory toxicities or other major safety concern identified by the LMM or DSMB. The trial may also be suspended by the IRBs if deemed necessary.

# 5 STUDY INTERVENTION/INVESTIGATIONAL PRODUCT

## 5.1 Study product descriptions

### Acquisition

The candidate antigen, FMP2.1, has been developed and manufactured and will be provided by WRAIR, and will be shipped from the US to MRTC in Bamako, under documented temperature-controlled conditions with temperature monitoring following SOPs.

The adjuvant AS02A is manufactured and provided by GSK and will be shipped from Rixensart, Belgium, to the MRTC in Bamako, under documented temperature-controlled conditions with temperature monitoring following SOPs.

The rabies vaccine will be purchased by the University of Maryland from the manufacturer and shipped to MRTC in Bamako, under documented temperature-controlled conditions with temperature monitoring following SOPs.

All three of these products have been successfully shipped to Mali for the two previous trials of the FMP2.1/AS02A vaccine. All are single-use products.

### Formulation and packaging

**FMP2.1**

Each vial of the FMP2.1 antigen contains 60 μg of lyophilized protein.

Active Ingredient: 0.100 mg/mL Formulated Bulk FMP2.1 when reconstituted to 0.60 mL volume

Formulation Buffer: 23.5 mM Sodium phosphate
0.1 mM EDTA
3.15% Sucrose
30 mM Sodium chloride

**AS02A adjuvant**

The FMP2.1 will be reconstituted in AS02A adjuvant. AS02A contains 50 μg MPL, 50 μg QS21, and 0.250 mL of proprietary oil/water emulsion in phosphate buffered saline (PBS) per volume of 0.5 mL. The AS02A adjuvant will be supplied as pre-filled syringes. The prefilled syringes will contain approximately 0.60 mL of liquid and will be stored at 2ºC to 8ºC with temperature monitoring, following SOPs.

**RabAvert/Rabipur® vaccine**

Our three previous Phase 1 malaria vaccine trials of the FMP2.1/AS02A vaccine have used as a control the RabAvert/Rabipur rabies vaccine, produced by Chiron Vaccines and sold under the name RabAvert in the U.S. under FDA approval and as Rabipur outside the U.S. under WHO approval. It is a sterile freeze-dried vaccine obtained by growing the fixed-virus strain Flury LEP in primary cultures of chicken fibroblasts. The strain Flury LEP was obtained from American Type Culture Collection as the 59th egg passage. The growth medium for propagation of the virus is a synthetic cell culture medium with the addition of human albumin, polygeline (processed bovine gelatin) and antibiotics. The virus is inactivated with propiolactone, and further processed by zonal centrifugation in a sucrose density-gradient. The vaccine is lyophilized after addition of a stabilizer solution which consists of buffered polygeline and potassium glutamate. One dose of reconstituted vaccine contains less than 12 mg polygeline (processed bovine gelatin), 1 mg potassium glutamate and 0.3 mg sodium EDTA. Small quantities of bovine serum are used in the cell culture process. Bovine components originate only from source countries known to be free of bovine spongiform encephalopathy. Minimal amounts of chicken protein may be present in the final product; ovalbumin content is less than 3 ng/dose (1 mL), based on ELISA. Antibiotics (neomycin, chlortetracycline, amphotericin B) added during cell and virus propagation are largely removed during subsequent steps in the manufacturing process. In the final vaccine, neomycin is present at < 1 µg, chlortetracycline at < 20 ng, and amphotericin B at < 2 ng per dose. RabAvert/Rabipur is intended for intramuscular (IM) injection. The vaccine contains no preservative and should be used immediately after reconstitution with the supplied Sterile Diluent for RabAvert/Rabipur (Water For Injection). The potency of the final product is determined by the NIH mouse potency test using the US reference standard. The potency of one dose (1.0 mL) RabAvert/Rabipur is at least 2.5 IU of rabies antigen. RabAvert/Rabipur is a white, freeze-dried vaccine for reconstitution with the diluent prior to use; the reconstituted vaccine is a clear to slightly opaque, colorless suspension.

## Product storage and stability

The FMP2.1 antigen, rabies vaccine, and AS02A pre-filled syringes will be stored at 2 to 8 ºC. Vaccines will be kept in a cold room or refrigerator that has 24-hour temperature recording. A back-up refrigerator and generator will be available in case of breakdown/power failure. The refrigerator that holds the vaccines and adjuvant will be locked. Records will be maintained that document receipt, release for immunization, disposal or return to the manufacturer of all vaccine vials. Copies of these records will be provided to the sponsors upon request for archiving. All study records will be kept in locked metal boxes.

The FMP2.1 antigen vials will be transported under controlled temperature conditions to the MRTC in Bamako, Mali. Temperature recorders will document maintenance of required temperature ranges. The AS02A adjuvant will be similarly transported from Belgium. FMP2.1 antigen, comparator vaccine and adjuvant will be stored in a cold room in the MRTC laboratory in Bamako until a few days before each vaccination is scheduled in Bandiagara. These will be transported in temperature-controlled conditions from Bamako to Bandiagara in the same containers used for

shipping to Mali. Cold chain deviations for vaccines or adjuvant will be recorded and reported promptly to sponsors and partners.

## 5.2 Dosage, preparation and administration of investigational product

The Investigators will maintain detailed SOPs for vaccine transport, storage, formulation, reconstitution and administration. Staff and investigators will be trained by senior investigators, the clinical coordinator and/or the senior study pharmacist in the SOPs relevant to their duties and will sign copies of the SOPs to document this training. Copies of SOPs will be available for inspection and review by the DMID, USAMMDA, GSK, WRAIR and study monitors. The study pharmacist and assistant study pharmacist will bear sole responsibility for storing, preparing, monitoring, and reconstituting vaccine and adjuvant from the time of delivery to the MRTC in Mali to the time syringes containing reconstituted vaccine and adjuvant are passed to physicians to administer to participants.

### Administration of vaccines

Vaccines will be administered by physician-investigators not involved with assessing adverse events. Each 0.5 mL of FMP2.1/AS02A or 1.0 mL of rabies vaccine will be administered by intramuscular injection in alternating deltoid muscles promptly after formulation/reconstitution. Each vial will be used for only one dose for one participant. If the preferred site for injection is contraindicated or not advisable such as in the case of a condition involving pain, infection, scarring that will make local reactions difficult to assess, or if the study participant or parent/guardian declares a preference for immunization in the alternative site, either deltoid muscle may be used.

## 5.3 Accountability procedures for the investigational product

In Mali, only the study pharmacists will have access to vaccines. The Vaccine Log Book will be used to record use and final disposition of each vial of FMP2.1 and adjuvant. Used vaccine vials, as well as unused vials, will be kept until such time as the PI and USAMMDA agree that there are no concerns about vaccine accountability and that they may be destroyed or returned according to instructions from USAMMDA.

## 5.4 Concomitant medications/treatments

At each study visit/contact, investigators will question the participant and their parents/guardians about any medication taken, including traditional medicines. Concomitant medication, including any vaccine other than the study vaccines, and any other medication relevant to the protocol, including any specifically contraindicated or administered during the period starting from one week before each study immunization and ending one month (maximum 30 days) after will be recorded with trade name and/or generic name of the medication, medical indication, start and end dates of treatment.

# 6 STUDY SCHEDULE

## 6.1 Screening

Recruitment will be progressive until 400 children of either gender who fulfill the inclusion criteria are included. Approximately 800-1000 children are likely to need to be screened to identify 400 eligible for the study. Participants will be recruited among children aged 1-6 years of age residing in Bandiagara. After community information is disseminated as described in Section 15.3, all interested parents of potentially eligible children will be invited to visit the study clinic on a specific date. After the study has been explained to the parents of the potential participants they will be provided with a copy of the consent form and may leave and return later with their decision; this will allow time for them to discuss the study with their family and carefully consider their child's involvement in the study. The individual consent process will be conducted in private rooms in the BMP clinic to ensure confidentiality.

All screening tests, medical history and examinations will be performed only after screening consent is obtained. Study clinicians will generally handle acute, simple conditions such as malaria or other acute infections. More complicated or chronic conditions, such as chronic renal or heart disease, will be referred to appropriate sources of medical care.

A screening form will be prepared for each participant and will later become part of the CRF for participants enrolled in the vaccine trial. A unique screening identification number will be assigned to each study participant. A medical history will be taken with special attention to recurrent infections to suggest immune suppression, previous history of splenectomy and prior vaccine reactions. Physical examination and laboratory screening tests will include: complete blood count (CBC) creatinine, ALT, and urine dipstick analysis. Unused blood from screening tests may be preserved for parasite genotyping. A participant who meets any of the exclusion criteria will be excluded. Participants excluded from this study because of significant abnormalities will be managed initially by study clinicians and referred to the local health center for evaluation as necessary. Screening tests will be completed within 45 days prior to entry into the study. Laboratory studies may be conducted at other times during the course of the trial if the investigators judge it necessary for the safety of the participant. Screening and follow-up diagnostic laboratory testing will be performed at the MRTC laboratory in Bandiagara and if applicable at the MRTC clinical laboratory in Bamako. Recruitment will continue until 400 eligible participants have fulfilled all of the inclusion criteria and none of the exclusion criteria, and signed the study consent form. Should parents of participants change their mind and decline to participate prior to immunization additional participants will be screened until 400 participants are enrolled. At the end of the screening process, a list of eligible children will be generated and used to identify the participants eligible for immunization.

## 6.2 Enrollment/baseline

Participant ID numbers will be assigned to participants in the order in which they come to BMP clinic on the day of immunization. Immediately after the first immunization, each study subject will receive a photo ID card. The vaccine that is assigned during the first immunization will be maintained for second and third immunizations.

## 6.3 Vaccination process

Before each vaccination, criteria for continued eligibility will be reviewed and verified. A history-directed physical examination will be done and temperature, blood pressure, pulse, respiratory rate and baseline general symptoms will be recorded. Venous blood will be collected for laboratory analysis.

After the participant's identity is checked by comparing his/her name with the list of eligible children, he/she will be vaccinated by intramuscular injection into alternating deltoid muscles. If any local impairment prevents administration of the vaccine into the preferred deltoid for that particular immunization, the vaccine may be administered into the opposite deltoid. Vaccination will be done on study days 0, 30 +/- 7 and 60 +/- 7.

The participant ID number will be assigned only to children immunized with the first dose. After immunization, an ID photo card with the unique participant ID number assigned to children will be prepared.

## 6.4 Follow-up

After each immunization, participants will be observed for local and systemic reactions for a minimum of 60 minutes. Physicians trained in pediatric resuscitation will be present on site on immunization days. Signs and symptoms will be solicited from participants and parents/guardians and recorded by the investigators. Participants will then be followed for a seven-day surveillance period after each vaccination (including day 0, the day of vaccination). All adverse events occurring during this seven-day period will be followed until resolution or stabilization. If any symptom persists beyond the seven-day surveillance period the participant will be followed daily until resolution of the adverse event.

After the seven-day surveillance period, participants and their parents/guardians will be asked to come to the BMP clinic center on day 30 +/- 7 after vaccination. At each visit, a study physician will evaluate the participants. A clinical examination will be performed and information on any solicited or unsolicited symptoms since the last visit will be collected. Every effort will be made to ensure compliance with visits. Local guides will conduct home visits to participants 1-2 days before their scheduled visit at the clinic. If a participant does not appear for a scheduled clinic visit, the local guide will visit him/her again and accompany the participant to the clinic center. If an SAE has occurred, appropriate measures will be taken to notify the PI, Local Medical Monitor, DSMB, USAMMDA, DMID, and all IRBs as described in Section 10.7.

# 7 METHODS FOR MEASURING EFFICACY

The goal for a blood stage malaria vaccine is to reduce severe disease and death, rare events that would require very large samples sizes. An expert panel convened by the WHO in October 2006 reached consensus that time to first clinical malaria episode using a site-specific definition of clinical malaria is the most suitable study endpoint that is most likely to be predictive of severe disease and death in early efficacy trials of malaria vaccines (V. Moorthy et al., report in preparation). However, no blood stage malaria vaccine has shown protective efficacy in clinical trials, so there is no validated endpoint for such trials. It is therefore prudent to include clinical, parasitological, molecular, and immunological secondary and exploratory endpoints in this trial, for three reasons: 1) to confirm and characterize any efficacy measured using the primary endpoint; 2) to allow for th e possibility that other endpoints might show efficacy when the primary endpoint does not, and avoid discarding a promising vaccine candidate; and 3) so that these surrogate means of measuring vaccine effect can be assessed and refined for future malaria vaccine efficacy trials.

## 7.1 Follow up methods

### Passive case detection

For the primary efficacy endpoint, passive case detection will consist of continuous availability of free, expeditious, high quality medical care at the BMP research clinic and Bandiagara District Hospital, including rapid microscopic diagnosis of malaria. Based on ten years of longitudinal outpatient studies at this site we expect near-exclusive utilization of the clinic and hospital. All study participants will reside within 2 km of the clinic. The only other modern health facility in the town is a maternity clinic that does not treat children. Local traditional healers are used but we have enlisted the support of this group with referring children with medical problems to the clinic.

The outpatient research clinic will be staffed by study physicians and laboratory staff 7 days a week, and the study physicians will be on call 24 hours a day, 7 days a week to assess cases of severe malaria or other medical emergencies. An effective on-call system has been in effect since we began studies of severe malaria at this site in 1997. Nursing staff are present at the Bandiagara District Hospital pediatric ward 24 hours a day, 7 days a week. When a study participant presents at any time when the outpatient research clinic is closed, a guard is promptly dispatched to the project staff residence to summon a research physician.

### Active surveillance

Active surveillance to detect asymptomatic malaria infection and anemia will consist of the scheduled visits during the immunization phase of the trial followed by six additional scheduled monthly visits over the course of the 2007 malaria transmission season, for clinical assessment, malaria smear and hemoglobin determination. An extended follow-up period will include active

follow-up visits on study days 364 (± 14 days), 547 (± 30 days) and 730 (± 30 days), for safety evaluations and for the secondary assessment of duration of efficacy and dynamics of antibody responses.

Consistent with standard clinical practice in settings with high prevalence of asymptomatic malaria infection and with many previous clinical and epidemiological studies at this site, routine scheduled malaria smears will not be read immediately unless symptoms are present. Asymptomatic infections will therefore only be detected retrospectively. Based on our past experience at this site, very few clinical cases of malaria are expected to be identified through active case detection, but should symptoms suggestive of malaria be identified during scheduled visits, participants will be brought to the clinic for the same full assessment given to self-referred participants.

## 7.2 Case definitions

### Clinical malaria

For the primary endpoint, a clinical malaria episode will be defined as symptoms generally consistent with malaria (including but not limited to headache, body aches, fever, chills, weakness), accompanied by documented axillary temperature of 37.5 $^{o}$C or greater, and an asexual *P. falciparum* parasitemia of 2500/mm$^3$ or greater.

This parasitemia was determined for Bandiagara based on the methods of Smith et al. (55). This method for modeling fever as a continuous function of parasitemia was recently recommended by a WHO expert panel as the most appropriate method for choosing parasitemia thresholds for efficacy trials of malaria vaccines. A threshold that results in at least 80% specificity was recommended by that group, but others have proposed using 95% specificity when possible. We conducted two surveys in Bandiagara in 2000 and 2001 and found that any level of parasitemia had more than 85% specificity for clinical malaria. To improve the specificity of the case definition at this site, we chose 2500/mm$^3$, which had 95% specificity and more than 85% sensitivity for clinical malaria in both years of the study.

### Anemia

Anemia is defined as hemoglobin of less than 8.4 g/dL. Severe anemia is defined as hemoglobin of less than 5 g/dL.

### Severe malaria

Severe malaria is defined according to WHO protocols (56) modified to include two additional criteria based on our published results from this site (6). These criteria are:

- Coma (Blantyre Coma Scale ≤ 2)
- Seizure (one or more witnessed by the investigators)
- Obtundation (depressed consciousness with BCS >2)

- Parasitemia $\geq$ 500,000/mm$^3$
- Lethargy or prostration (clinical judgment or child $\geq$ 7 months unable to sit unassisted)
- Severe anemia (hemoglobin $\leq$ 5 g/dl)
- Respiratory distress (intercostal muscle retraction, deep breathing, grunting)
- Hypoglycemia (glucose $\leq$ 40 mg/dl)
- Jaundice
- Renal insufficiency as indicated by lack of urination for $\geq$ 1 day
- Gross hematuria
- State of shock (systolic blood pressure $\leq$ 50 mm Hg, rapid pulse, cold extremities)
- Inability to eat or drink or protracted vomiting (added based on our experience)

## 7.3 Primary efficacy endpoint

- Time to first clinical malaria episode (as defined above) occurring between randomization and six months after assigned date of the third immunization

## 7.4 Secondary efficacy endpoints

- Time to first clinical malaria episode with parasites with AMA-1 genotypes identical to the 3D7 vaccine strain with respect to designated polymorphic codons

- Incidence density of clinical malaria episodes occurring between randomization and six months after assigned date of the third immunization

- Time to first clinical malaria episode occurring during two years after randomization

- Incidence density of clinical malaria episode occurring during two years after randomization

## 7.5 Exploratory efficacy endpoints

- Time to first clinical episodes of malaria using increasingly specific definitions of clinical episodes (parasitemia thresholds of any parasitemia, 100, 1000, 2500, 5000, 10,000, 20,000, 50,000 and 100,000/mm$^3$; and any symptoms consistent with malaria, history of fever) occurring between randomization and six months after assigned date of the third immunization

- Asexual *P. falciparum* parasite density measured as area under the curve

- Gametocyte counts measured as area under the curve

- Incidence of anemia and severe anemia

# 8 STUDY PROCEDURES/EVALUATIONS

## 8.1 Standard operating procedures (SOPs)

The Investigators will maintain detailed SOPs for vaccine transport, storage, formulation, reconstitution and administration. All staff and investigators will be trained in the SOPs relevant to their duties and will sign copies of the SOPs to document this training. Copies of SOPs will be available for inspection and review by the DMID, USAMMDA, GSK, WRAIR and study monitors. During the study SOPs may be modified to improve them and new SOPs may be developed as needed to improve operations and ensure adherence with the protocol.

The following SOPs will be followed (additional SOPs may be developed and added):

- Recruitment
- Community informed consent
- Individual informed consent
- Field team organization
- Physical examination
- Management of anaphylaxis
- Case management of uncomplicated and severe malaria
- Management of abnormal laboratory tests
- Completion of CRFs
- Transport of vaccines and adjuvants
- Investigational product labeling
- Vaccine preparation and administration
- Vaccine accountability, handling, storage, disposition and destruction
- Randomization
- Active and passive follow up
- Unblinding
- Quality control and quality assurance
- Study documentation
- Adverse event reporting
- Protocol deviations
- SAE reporting
- Provisions for study suspension
- Malaria thick smear preparation and reading
- Clinical management of uncomplicated and severe malaria
- Specimen collection and handling
    - Blood collection
    - Urine collection
    - Specimen transport

    - Specimen reception
    - Serum separation
    - Specimen storage
    - Specimen shipping overseas
- Waste management
    - Sharps handling and disposal
    - Biologic waste handling and disposal
    - General waste handling and disposal
- Safety
    - Spill management
    - Exposure to body fluids
- Equipment maintenance and quality control SOPs (Reflotron PLUS, AcT-Diff, Centrifuge)

## 8.2 Daily study procedures

**Day -45 to –1 Screening /inclusion of participants**

Meetings will be held with town administrative and medical authorities to explain the purpose of the study. These meetings will be followed by meetings with the traditional authorities and community leaders to request community-level "permission to enter." Subsequently, general information about the study will be disseminated through the local radio station. The target population will be invited for screening as described in subject recruiting SOPs. Screening will be performed until 400 eligible participants are identified.

**Screening (may take place over more than one visit) up to 45 days prior to vaccination**

- Written informed consent for screening
- Medical history of participant
- Complete physical examination
- Collect 2-3 mL venous blood sample for:
    - Hematology: white blood cell count, lymphocyte count, hemoglobin, platelets.
    - Biochemistry: serum creatinine and ALT
    - Hepatitis B surface antigen
    - Remaining unused blood will be preserved for parasite genotyping
- Collect urine: dipstick for blood, glucose and protein
- Check of inclusion and exclusion criteria
- Written informed study consent for vaccination

**Day 0: Vaccination 1**

Before vaccination:

- Review screening laboratory test results
- Review inclusion/exclusion criteria and check of contraindications/precautions

- Record any complaints, symptom-directed physical examination, and examination of the immunization site(s) for any abnormalities.
- Record vital signs: temperature, blood pressure, pulse, respiratory rate
- Record baseline data for solicited general symptoms
- Collect finger-stick blood for hemoglobin determination, malaria smear and parasite genotyping
- Collect 7-10 mL venous blood sample for:
  - CBC, creatinine, ALT
  - Serum for anti-FMP2.1 antibodies and GIA
  - Peripheral blood mononuclear cells (PBMC) for cell-mediated immune responses (CMI) to AMA-1
  - Parasite genotyping
- Assignment of unique participant ID number
- Administer study immunization 1

After vaccination:

- Observe for a minimum of 60 minutes
- Record blood pressure, pulse, respiratory rate, temperature
- Record solicited and unsolicited events
- Prepare an ID card containing participant's unique participant ID number and photo
- Instruct participants and their parents/guardians to return to the BMP clinic center immediately should they manifest any signs or symptoms they perceive as serious.

**Days 1-3: Days 1, 2, 3 post-vaccination 1 surveillance visits**

- Brief medical history
- Record temperature
- Record solicited and unsolicited signs/symptoms
- Targeted physical examination including immunization site

**Day 7± 1 day: 7 days post-vaccination 1 surveillance visit**

- Brief medical history
- Record temperature
- Targeted physical examination including immunization site
- Record any solicited and unsolicited adverse events occurring after the last study immunization
- Collect 2-3 mL of venous blood for CBC, creatinine and ALT and parasite genotyping

**Day 30± 7 days: 1 month post-vaccination 1 surveillance visit and vaccination 2**

Before vaccination:

- Check participant's ID to confirm identity
- Brief medical history
- Targeted physical examination including immunization site(s)
- Check of contraindications/precautions

- Record vital signs: temperature, blood pressure, pulse, respiratory rate
- Review medical history and record any unsolicited adverse events occurring since last visit
- Record baseline data for solicited general symptoms
- Collect finger-stick blood for hemoglobin determination, malaria smear and parasite genotyping
- Collect 7-10 mL venous blood sample for:
    - CBC, creatinine, ALT
    - Serum for anti-FMP2.1 antibodies and GIA
    - PBMC for CMI
    - Parasite genotyping
- Administer study immunization 2

After vaccination:

- Observe for at least 60 minutes
- Record blood pressure, pulse, respiratory rate, temperature
- Record solicited and unsolicited adverse events
- Instruct participants and their parents/guardians to return to the BMP clinic center immediately should they manifest any signs or symptoms they perceive as serious.

**Days 31-33: Days 1, 2, 3 post-vaccination 2 surveillance visits**

- Brief medical history
- Record temperature
- Record solicited and unsolicited signs/symptoms
- Targeted physical examination including immunization site(s)

**Day 37 ± 7 days: Day 7 post-vaccination 2 surveillance visit**

- Brief medical history
- Record temperature
- Record solicited and unsolicited signs/symptoms
- Targeted physical examination including immunization site(s)
- Collect 2-3 mL of venous blood for CBC, creatinine and ALT and parasite genotyping

**Day 60± 7 days: 1 month Post-vaccination 2 surveillance visit and vaccination 3**

Before vaccination:

- Check participant’s ID to confirm identity
- Brief medical history
- Targeted physical examination including immunization site(s)s
- Check of contraindications/precautions
- Record vital signs: temperature, blood pressure, pulse, respiratory rate
- Review medical history and record any unsolicited adverse events occurring since last visit
- Record baseline data for solicited general symptoms

- Collect finger-stick blood for hemoglobin determination, malaria smear and parasite genotyping
- Collect 7-10 mL venous blood sample for:
  - CBC, creatinine, ALT
  - Serum for anti-FMP2.1 antibodies and GIA
  - PBMC for CMI
  - Parasite genotyping
- Administer study immunization 3

After vaccination:
- Observe for at least 60 minutes
- Record blood pressure, pulse, respiratory rate, temperature
- Record solicited and unsolicited adverse events
- Instruct participants and their parents/guardians to return to the BMP clinic center immediately should they manifest any signs or symptoms they perceive as serious.

**Days 61-63: Days 1, 2, 3 post-vaccination 3 surveillance visits**
- Brief medical history
- Record temperature
- Record solicited and unsolicited signs/symptoms
- Targeted physical examination including immunization site(s)

**Day 67 ± 7 days: Day 7 post-vaccination 3 surveillance visit**
- Brief medical history
- Record temperature
- Record solicited and unsolicited signs/symptoms
- Targeted physical examination including immunization site(s)
- Collect 2-3 mL of venous blood for CBC, creatinine and ALT and parasite genotyping

**Day 90± 10 days: 30 days Post-vaccination 3 surveillance visit**
- Brief medical history
- Record temperature
- Targeted physical examination including immunization site(s)
- Record any unsolicited adverse events occurring since last visit
- Collect finger-stick blood for hemoglobin determination, malaria smear and parasite genotyping
- Collect 7-10 mL of venous blood for:
  - Serum for anti-FMP2.1 antibodies and GIA
  - PBMC for CMI
  - CBC, creatinine and ALT
  - Parasite genotyping

**Day 120± 14 days: Monthly Post-vaccination surveillance visit**

- From Day 120 through Day 240 (six months after the third immunization) participants and their parents/guardians are encouraged to continue to attend the BMP clinic any time they are sick. A malaria smear and hemoglobin determination will be done whenever symptomatic malaria is suspected. During this period participants and their parents/guardians will be asked to return to BMP clinic center every month ±14 days.
- Brief medical history
- Record temperature
- Targeted physical examination including immunization site(s)
- Record any serious adverse events occurring after the last study immunization
- Collect finger-stick blood for hemoglobin determination, malaria smear and parasite genotyping

**Day 150± 14 days: Monthly Post-vaccination surveillance visit with lab tests**

- Brief medical history
- Record vital signs
- Targeted physical examination including immunization site(s)
- Collect finger-stick blood for hemoglobin determination, malaria smear and parasite genotyping
- Collect 7-10 mL venous blood for:
    - Serum for anti-FMP2.1 antibodies and GIA
    - PBMC for CMI
    - Parasite genotyping

**Day 180± 14 days, Day 210± 14 days: Monthly post-vaccination surveillance visits**

- Participants and their parents/guardians are encouraged to continue to attend the BMP clinic any time they are sick. A malaria smear and hemoglobin determination will be done whenever symptomatic malaria is suspected.
- During this period participants and their parents/guardians will be asked to return to BMP clinic center every month ±14 days.
- Brief medical history
- Record temperature
- Targeted physical examination including immunization site(s)
- Record any serious adverse events occurring after the last study immunization
- Collect finger-stick blood for hemoglobin determination and parasite genotyping

**Day 240± 14 days: Final Post-vaccination surveillance visit for primary analysis**

- Brief medical history
- Record vital signs
- Targeted physical examination including immunization site(s)
- Collect finger-stick blood for hemoglobin determination, malaria smear and parasite genotyping
- Collect 7-10 mL venous blood for:
    - Serum for anti-FMP2.1 antibodies and GIA

- PBMC for CMI
- Parasite genotyping

**NOTE:** Data up to this point will be included in the primary unblinded data analysis. Although clinical investigators will not be directly informed of vaccine assignments, the study will technically be single-blind from this point forward. Participants will remain blinded to vaccine assignment until the end of the study.

**Day 364± 14 days Day 547± 30 days, Day 730± 30 days: Post-vaccination safety and extended efficacy surveillance period**

- Participants and their parents/guardians are encouraged to continue to attend the BMP clinic any time they are sick. A malaria smear and hemoglobin determination will be done whenever symptomatic malaria is suspected.
- Brief medical history
- Record vital signs
- Targeted physical examination including immunization site(s)
- Collect finger-stick blood for hemoglobin determination, malaria smear and parasite genotyping
- Collect 7-10 mL venous blood for:
    - Serum for anti-FMP2.1 antibodies and GIA
    - PBMC for CMI
    - Parasite genotyping

## Outline of study procedures

**Table 14 Summary of study procedures**

| **Study Days** | **-45 to –1 Screening** | **0** | **1-3** | **7** | **30** | **31-33** | **37** | **60** | **61-63** | **67** | **90** | **120** | **150** | **180-210** | **240** | **364-730** |
|---|---|---|---|---|---|---|---|---|---|---|---|---|---|---|---|---|
| **Clinic Visit** | **1** | **2** | **3-5** | **6** | **7** | **8-10** | **11** | **12** | **13-15** | **16** | **17** | **18** | **19** | **20-21** | **22** | **23-25** |
| Village & family information & discussion | ● | | | | | | | | | | | | | | | |
| Written individual Screening Consent | ● | | | | | | | | | | | | | | | |
| Check of inclusion/exclusion criteria | ● | ● | | | | | | | | | | | | | | |
| Check of contraindications to immunization | | ● | | | ● | | | ● | | | | | | | | |
| Written individual Study Consent | | ● | | | | | | | | | | | | | | |
| Medical history<br>Physical examination | ●<br>● | ●<br>● | ●<br>● | ●<br>● | ●<br>● | ●<br>● | ●<br>● | ●<br>● | ●<br>● | ●<br>● | ●<br>● | ●<br>● | ●<br>● | ●<br>● | ●<br>● | ●<br>● |
| **Vaccination** | | ● | | | ● | | | ● | | | | | | | | |
| Post-vaccination recording of solicited AE | | ● | ● | ● | ● | ● | ● | ● | ● | ● | | | | | | |
| Recording of unsolicited AE occurring up to one month post-vaccination | | ● | ● | ● | ● | ● | ● | ● | ● | ● | ● | | | | | |
| Recording of medication | | ● | ● | ● | ● | ● | ● | ● | ● | ● | ● | ● | ● | ● | ● | ● |
| Recording of SAEs during the study period | | ● | ● | ● | ● | ● | ● | ● | ● | ● | ● | ● | ● | ● | ● | ● |
| Urine analysis for blood, glucose and protein | ● | | | | | | | | | | | | | | | |
| CBC | ● | ● | | ● | ● | | ● | ● | | ● | ● | | | | | |
| Serum chemistry (Creatinine, ALT) | ● | ● | | ● | ● | | ● | ● | | ● | ● | | | | | |
| Hepatitis B surface antigen | ● | | | | | | | | | | | | | | | |
| Serum and cells for anti-AMA-1 response | | ● | | | ● | | | ● | | | ● | | ● | | ● | ● |
| Venous blood for parasite genotyping | ● | ● | | ● | ● | | ● | ● | | ● | ● | | ● | | ● | ● |
| Fingerstick blood for parasite genotyping, malaria smear and hemoglobin | | ● | | | ● | | | ● | | | ● | ● | ● | ● | ● | ● |
| Scheduled blood volume (mL) | 2-3 | 7-10 | 0 | 2-3 | 7-10 | 0 | 2-3 | 7-10 | 0 | 2-3 | 7-10 | ≤0.2 | 7-10 | ≤0.2 | 7-10 | 7-10 |
| Cumulative Blood Volume (mL) | 2-3 | 9-13 | 9-13 | 11-16 | 18-26 | 18-26 | 20-29 | 27-39 | 27-39 | 29-42 | 36-52 | 36-52 | 43-62 | 43-62 | 50-72 | 71-102 |

## Health care provision

Routine and emergency health care will be provided by study clinicians at the Bandiagara District Hospital, in collaboration with District Hospital physicians. The clinical research facility includes private consultation rooms, a procedure room, a resuscitation suite with oxygen, suction and resuscitation kits, and a post-immunization observation room. An ambulance with suction and

oxygen will be on site on immunization days. The pharmacy at the BMP clinic will have sufficient stocks to provide participants with oral and parenteral drugs for the treatment of common illnesses (including uncomplicated and severe malaria) free of charge, using essential medicines and treatment regimens that meet or exceed standards recommended by the Mali Ministry of Health. Blood transfusion will be available at all times. Twenty-four hour hospitalization and basic emergency surgery services are available at the Bandiagara Hospital adjacent to the BMP clinic, in a pediatric ward that was renovated and upgraded in 2006. Twenty-four hour nursing staff and on call physicians will be available.

If the investigators or the Local Medical Monitor judge that a participant requires hospitalization at the National Hospital in Bamako (approximately 8 hours drive from Bandiagara), referral and transportation will be arranged and the medical management of the participants will be monitored by senior physician investigators and/or the Local Medical Monitor. The regional hospital in Mopti, a 45-minute drive from Bandiagara, has radiography, medical subspecialty and surgical capabilities. The national hospital in Bamako has these capacities and in addition has an intensive care unit with mechanical ventilation, a computerized axial tomography scanning facility and other advanced medical and surgical care.

Cases of clinical malaria will be managed according to Malian National Malaria Control guidelines and the SOP “Case management of uncomplicated and severe malaria”.

## 8.3 Final study visit

At the time of the final study visit (day 730 +/- 30 days), participants and their parents/guardians will be debriefed, including instruction to return to the BMP clinic at any time in the future if they feel they may have a medical problem related to their participation in the study. Any AEs or SAEs that are unresolved at that time will be continue to be followed until resolution, or, if a chronic condition has developed, until it has stabilized.

## 8.4 Early termination visit

If early termination after the first vaccination occurs more than one week after a scheduled visit has been completed and the participant and their parents/guardians are willing to have evaluations performed, a physical examination should be performed and, if more than two weeks have elapsed since the last time venous blood was drawn, 7-10 mL venous blood may be drawn for CBC, creatinine and ALT determination, and/or for serology. Any participant who has received one or more immunizations will be followed for the duration of the study even if it is determined that the second or third immunization should not be administered. Participants who exit the study after receiving one or more immunizations will not be replaced by new participants.

## 8.5 Unscheduled visit

Unscheduled visits will prompt a history and physical examination, clinical laboratory tests including malaria smear if indicated, documentation of any AEs, and any other medically indicated diagnostic or therapeutic procedures. These will be recorded as observations in the participant's study record.

# 9 Sample handling and analysis

## 9.1 Overview of sample collection, handling, transport and shipping.

Detailed SOPs are maintained for these activities. Briefly, finger-prick blood and venous blood are obtained at the BMP research clinic and processed in the sample processing laboratory according to SOPs. Filter paper blood samples are stored at room temperature in sealed desiccant pouches, and sera, plasma, cells and parasites are frozen at -80$^{o}$C or in liquid nitrogen containers. Frozen samples are transported in project vehicles to the main MRTC immunology laboratory in Bamako in liquid nitrogen dry shippers, and either stored there in a liquid nitrogen storage system or shipped to the CVD in dry shippers. This cold chain has been successfully maintained since 1999.

## 9.2 Overview of collection time points

Blood will be collected from study participants by venipuncture up to 14 times during the study, including screening. The maximum amount of blood requested from any participant for standard collection during the study for research purposes will not exceed 102 mL over the one-year study period. However, additional blood may be obtained as deemed necessary by the investigators or clinicians to evaluate a medical illness or condition. Fingerstick blood ($\leq$ 0.2 mL) will be collected for research purposes on five occasions and whenever deemed clinically necessary to diagnose malaria and measure hemoglobin.

### Safety

Tests for CBC, creatinine, and ALT, at screening, and on study days 0, 7, 30, 37, 60, 67 and 90 (intervals between study visits may deviate from this schedule by the ranges indicated in Section 8 above) will be performed at the MRTC clinical laboratory in Bandiagara, with back-up testing (serological testing for hepatitis and other conditions, liver function tests, etc.) available at the MRTC clinical laboratory in Bamako if needed.

### Serology

Separation of serum/plasma from venous blood will be performed at the BMP clinic and samples will be aliquoted for later use to determine anti-FMP2.1 antibody assays at Day 0, 30, 60, 90, 150, 240, 364, 547 and 730 (intervals between study visits may deviate from this schedule by the ranges indicated in Section 8.2 above). The antibody assays on samples collected at day 90 and thereafter will provide information on the duration of vaccine-boosted antibody responses.

### CMI

CMI assays will be performed on PBMC samples collected on Day 0, 30, 60, 90, 150, 240, 364, 547 and 730 (intervals between study visits may deviate from this schedule by the ranges indicated in Section 8.2 above). The CMI assays on samples collected at day 90 and thereafter will provide information on the duration of vaccine-boosted CMI responses.

## 9.3 Laboratory Assays

The Investigators will maintain detailed SOPs for all laboratory assays at the BMP and central laboratories at MRTC, in Bamako. These SOPs will include sample collection, handling (e.g. serum separation), labeling, preservation (e.g. PBMC cryopreservation), storage, transport, and shipping. All staff and investigators will be trained in the SOPs relevant to their duties and sign copies of the SOPs to document this training. Copies of SOPs will be available for inspection and review by study monitors. The general methods that will be used are summarized in the following section.

### Hematology and biochemistry

A complete blood count (CBC) with automated differential cell count, serum creatinine and ALT tests will be measured at defined time points throughout the study period. The Principal Investigator will maintain laboratory reference intervals in the study file, and copies will be made available upon request to study monitors and sponsors. Hematology and serum biochemistry assays will be performed at the MRTC clinical laboratory in Bandiagara. In the rare event that a participant has traveled to Bamako, hematology and biochemistry tests may also be done in the MRTC laboratory in Bamako under study-specific SOPs.

Rapid test kits (Cortez Diagnostics, Calabasas, CA) will be used to test for Hepatitis B surface antigen at screening.

Urine will be collected for glucose, blood and protein determination using FDA-approved urinary reagent dipsticks.

### Serology

Serological assays for anti-FMP2.1 antibodies will be performed at the WRAIR laboratories in Silver Spring, US, following SOPs for methods that have been used for the four previous trials of this vaccine.

Immunogenicity will be determined by evaluating antibody (IgG) responses to the *P. falciparum* AMA-1 protein as measured using standard ELISA methodologies with appropriate capture antigens, namely the recombinant AMA-1 antigen that the vaccine is based upon. These results will be the main immunogenicity outcome measure for this trial. Secondary serological assays described below will not be begun until all primary humoral immunogenicity studies have been completed at WRAIR.

## Malaria smears

Both thick and thin smears will be obtained during the study. Parasite density will be quantified each time malaria smears are obtained, either at the time of the visit if the participant is ill or at a later time. Standard operating procedures are followed to assure uniform and high quality malaria smears. Thick smears are read by counting the number of parasites seen per 500 white blood cells. Parasite density will be calculated based on the most recent exact white blood cell count if one was performed within two weeks of the malaria smear. Otherwise, quantification will be based on an expected 8,000 white blood cells/$mm^3$. Thin smears are read by counting the number of parasites seen per 500 red blood cells. Exact parasite density is calculated based on the hemoglobin. Thin smears will be used to identify the malaria species each time the diagnosis of malaria is made.

Accurate speciation and quantification will be assured based on SOPs. Slides will be read by at least two readers with a tie-breaker in the case of significant discrepancies. Readers will have been trained in malaria diagnostic microscopy using the Malaria Research Reference and Reagent Resource (MR4) standardized slide set designed for this purpose, and, subject to continued availability of the slide set, will be evaluated annually using the slide set to assess their competency.

## Additional assays

Titers of AMA-1 antibodies measured by ELISA will be the main immunological endpoint. However, several other means of measuring the immune response are being assessed. If protective efficacy is measured in this trial, it will be possible to determine which measures of the immune response best correlate with this protection. These additional studies may help refine our understanding of the immune response to AMA-1 vaccines in ways that lead to development of improved immunogenicity assays for future vaccine trials. These immunological studies, in conjunction with AMA-1 sequencing studies and related in vitro studies conducted separate from this protocol, are aimed to improve our understanding of the degree to which the immune response to AMA-1 immunization is allele-specific or cross-reactive. These studies may inform further development and optimization of AMA-1 vaccines. To ensure appropriate use of sera and PBMC, these additional assays will be done in an order of priority agreed upon by the investigators.

### CMI and antibody avidity assays

PBMCs will be cryopreserved and transported to the University of Maryland for CMI assays. The objectives of CMI and secondary serological studies are twofold: (1) Accurately measure humoral immunity by quantifying antibody responses including subclass ($IgG_1$, $IgG_2$, $IgG_3$ and $IgG_4$) and assessing antibody avidity to the antigen AMA-1 and (2) Systematically analyze T-cell responses to both full-length recombinant AMA-1 (rAMA-1) antigen and to synthesized peptides derived from domain I of the AMA-1 ectodomain. Based on these results, we will address three important questions: (1) Is the level of anti-AMA-1 antibody levels in serum (as measured in end-point dilutions by ELISA) correlated with T-helper cell responses? (2) What are the characteristics of memory T cell responses? And (3) are the avidity and/or classes and IgG sub-classes of anti-AMA-1 antibodies correlated with memory and effector T-helper cell responses?

In addition to antibody levels as measured by ELISA titers, avidity of antibodies may be important measures of a protective immune response. Antibody avidity and affinity assays will be performed at the University of Maryland to test the hypothesis that the avidity of antibodies to AMA-1 correlates with allele-specific T-helper cell responses to AMA-1.

A list of assays follows. Results of these studies will not be used as measures of immunogenicity for purposes of choosing a dose for further clinical development. Methods for some of these assays are currently being developed and refined. As noted above, these assays will be prioritized by the investigators to ensure optimal use of limited sera and cells.

- Antibody avidity studies
- Antibody subclass analysis
- Identification of immunodominant T cell epitopes involved in class I (e.g., CTL) and II (e.g., Th1, Th2) MHC restricted T cell responses
- Measurement of cytokine-producing cells to soluble antigens by ELISPOT
- Cytokine measurements using optimized Cytometric Bead Array (CBA) technology
- Measurement of memory T cell subpopulations
- Measurement of memory B cell pools
- Lymphoproliferative responses to AMA-1

**Additional serology**

As a capacity-building exercise, WRAIR investigators will assist MRTC investigators with establishing the ability to perform serological assays for antibody (IgG) responses to the *P. falciparum* AMA-1 by using the same ELISA methodologies with appropriate capture antigens that will be used for the immunogenicity study endpoint at WRAIR. This process has begun with a three-month visit by an MRTC scientist to the WRAIR ELISA laboratory in 2005.

No additional blood will be drawn for these capacity-building assays. After final serological results from the reference immunology laboratory at WRAIR have been fully analyzed and reported, and if sufficient serum remains, these capacity building assays may be conducted and the results of the Mali assays may be compared to the WRAIR serological results, solely for the purposes of assessing how well the Malian laboratory was able to replicate the WRAIR results. It is emphasized that this is a capacity-building exercise and that only the serological results from the WRAIR Department of Immunology will be analyzed as trial endpoints.

**AMA-1 sequencing and genotyping**

We hypothesize that the frequency of malaria infections with AMA-1 sequence similar to the 3D7 vaccine strain will decrease in the FMP2.1/AS02A group relative to the control group, and that this decrease will be measurable in survival analysis of time to first clinical malaria episode with 3D7-type parasites with respect to polymorphisms in AMA-1. To obtain the samples to test this hypothesis, whenever venous blood is obtained for research purposes, blood cell pellets will be frozen for subsequent DNA extraction and genotyping. When sufficient volumes are available after blood is allocated for other uses, pellets may be cryopreserved for later expansion in culture for further parasite characterization by molecular and in vitro methods such as growth inhibition assays.

Whenever malaria smears are obtained by digital puncture, a few drops (less than 0.2 mL) of blood will be blotted onto filter paper and preserved for parasite DNA analysis. These samples will be subject to sequencing of AMA-1 to identify polymorphic amino acid residues that may be selected by the FMP2.1/AS02A vaccine and other parasite genotyping studies including microsatellite and genome-wide analyses to aid with characterization of vaccine-resistant strains.

# 10 ASSESSMENT OF SAFETY

## 10.1 Specification of safety parameters

The primary outcome measures for this trial are:

1. Occurrence of solicited symptoms after each vaccination during a seven-day surveillance period (day of vaccination and days 1, 2, 3 and 7 after vaccination)

2. Occurrence of unsolicited symptoms after each vaccination during a 30-day surveillance period (day of vaccination and 30 subsequent days)

3. Occurrence of serious adverse events throughout the study period

## 10.2 Methods and timing for assessing, recording, and analyzing safety parameters

### Adverse events

An adverse event includes any noxious, pathological or unintended change in anatomical, physiological or metabolic functions as indicated by physical signs, symptoms and/or laboratory detected changes occurring in any phase of the clinical study whether associated with the study vaccine or active comparator vaccine and whether or not considered vaccination related. This definition includes an exacerbation of pre-existing conditions or events, intercurrent illnesses, or vaccine or drug interaction. Symptomatic uncomplicated or severe malaria infection will be coded as adverse events as will any other acute illness, throughout the study. Anticipated day-to-day fluctuations of pre-existing conditions that do not represent a clinically significant exacerbation need not be considered adverse events. Discrete episodes of chronic conditions occurring during a study period will be reported as adverse events to assess changes in frequency or severity.

Adverse events will be documented in terms of a medical diagnosis. When it is not possible to make a specific medical diagnosis, the adverse event will be documented in terms of signs and/or symptoms observed by the investigator or reported by the subject at each study visit. Pre-existing conditions or signs and/or symptoms (including any that are not recognized at study entry but are recognized during the study period) present in a participant prior to the start of the study will be recorded on the participant's CRF. Any hospitalization will be considered a serious adverse event. Adverse events to be recorded as endpoints are described below. All other adverse events will be recorded as unsolicited adverse events.

### Surveillance period for adverse events

All adverse events occurring within 30 days following administration of each study immunization will be recorded irrespective of severity or whether or not they are considered vaccination-related.

Solicited adverse events will be elicited for a seven-day surveillance period (day of vaccination and study days 1, 2, 3 and 7) and unsolicited adverse events will be recorded during a 30-day surveillance period. Serious adverse events will be recorded throughout the study.

### Recording adverse events

At each visit/assessment, the investigator will evaluate all adverse events observed by the investigators or reported by the participant or their parents/guardians. New adverse events will be recorded in the Adverse Event form within the participant's CRF. Solicited and unsolicited adverse events will be recorded on separate pages of the CRF. The nature of each event, date and time (where appropriate) of onset, outcome, intensity and relationship to vaccination will be established. Any corrective treatment will be recorded in the CRF. As a consistent method of soliciting adverse events, the participant and their parents/guardians will be asked a non-leading question such as: "Have you felt different in any way since receiving the vaccine or since the last visit?" The investigator will record only those adverse events having occurred within the time frames defined above.

Adverse events already documented in the CRF, *i.e.* at a previous assessment and designated as 'ongoing' will be reviewed at subsequent visits, as necessary. If these events have resolved, the documentation in the CRF will be completed, including the date that the adverse event resolved. If an adverse event changes in frequency or intensity during a study period, the record will be updated to reflect the maximum intensity or describe the frequency.

### Solicited adverse events

**Local (injection site) adverse events**
Pain or tenderness at injection site
Swelling at injection site
Erythema at injection site

**General adverse events**
Fever (body temperature ≥ 37.5°C)
Drowsiness
Loss of appetite
Vomiting
Irritability/fussiness

Temperature will be recorded at the time of the clinic visit. If additional temperature measurements are recorded at another time of the day, the highest temperature will be recorded.

### Unsolicited adverse events

Unsolicited adverse events will be recorded in the CRF. Unsolicited adverse events are adverse events reported by the participants or accompanying guardians that are different from those solicited symptoms defined in the preceding section and/or that begin after the 7-day surveillance period for solicited adverse events.

## 10.3 Assessment of intensity of non-serious adverse events

For each solicited symptom the participants and their parents/guardians will be asked if they sought medical advice for this symptom. For all other adverse events than those in Table 15, maximum intensity will be assigned to one of the following categories:

0 = No adverse event

1 = An adverse event which is easily tolerated by the subject, causing minimal discomfort and not interfering with everyday activities.

2 = An adverse event that is sufficiently discomforting to interfere with normal everyday activities.

3 = An adverse event that prevents normal, everyday activities. Such an adverse event would for example prevent normal play or attendance at school and would require the administration of corrective therapy.

Intensity of the following adverse events will be assessed as described in Table 15:

**Table 15 Assessment of Solicited Adverse Event (AE) Intensity**

| AE | Grade | Intensity Definition |
|---|---|---|
| Pain/tenderness at injection site | 0 | Absent |
| | 1 | Minor reaction to touch |
| | 2 | Cries/protests on touch |
| | 3 | Cries when limb is moved/spontaneously painful |
| Swelling at injection site | 0 | Absent |
| | 1 | < 5 mm |
| | 2 | 5-20 mm |
| | 3 | > 20 mm |
| Erythema at injection site | 0 | Absent |
| | 1 | < 5 mm |
| | 2 | 5-20 mm |
| | 3 | > 20 mm |
| Limitation of arm motion - Abduction at the shoulder | 0 | None |
| | 1 | >90° but <120° |
| | 2 | >30° but ≤90° |
| | 3 | ≤30° |
| Reported arm motion limitation | 0 | Normal |
| | 1 | Using arm somewhat less than usual but easily tolerated |
| | 2 | Using arm less than usual / interferes with normal activity |
| | 3 | Avoiding use of arm |

| | | |
|---|---|---|
| Fever | 0<br>1<br>2<br>3 | < 37.5°C<br>37.5-38.0°C<br>38.1-39.0°C<br>> 39.0°C |
| Irritability/fussiness | 0<br>1<br>2<br>3 | Behavior as usual<br>Crying more than usual/ no effect on normal activity<br>Crying more than usual/ interferes with normal activity<br>Crying that cannot be comforted/ prevents normal activity |
| Drowsiness | 0<br>1<br>2<br>3 | Behavior as usual<br>Drowsiness easily tolerated<br>Drowsiness that interferes with normal activity<br>Drowsiness that prevents normal activity |
| Loss of appetite | 0<br>1<br>2<br>3 | Normal<br>Eating less than usual/ no effect on normal activity<br>Eating less than usual/ interferes with normal activity<br>Not eating at all |
| Vomiting | 0<br>1<br>2<br>3 | Absent<br>Occasional but able to eat/drink normal amounts<br>Repeated with limited oral intake<br>Continuous, unable to keep down liquids or solids |

**Table 16 Laboratory toxicity grading**

| **HEMATOLOGY** | | | | | |
|---|---|---|---|---|---|
| | Normal Range | Grade 1 | Grade 2 | Grade 3 | Grade 4 |
| Hemoglobin<br>Male and female | 8.4 – 12.4 g/dL[a] | 7.5 – 8.3 g/dL | 6.1 – 7.4 g/dL | 5.0 – 6.0 g/dL | < 5.0 g/dL |
| Platelets | 133,000 – 523,000/mm$^3$ [a] | 75,000 - 132,999/mm$^3$ | 50,000 - 74,999/mm$^3$ | 20,000 - 49,999/mm$^3$ or > 1,000,000/mm$^3$ | <20,000/mm$^3$ |
| WBCs | 5,300 – 15.300/mm$^3$ [a] | 15,299 – 16,500/ mm$^3$ or 3,501-5,299/mm$^3$ | 16,501-18,000 /mm$^3$ or 2,001-3,500/mm$^3$ | 18,001-30,000/mm$^3$ or 1,000-2,000/mm$^3$ | >30,000 or <1,000 /mm$^3$ |
| Absolute Lymphocyte Count | 2,300-9,500/mm$^3$ [b] | 1,000-2,299/mm$^3$ | 750-999/mm$^3$ | 500-749/mm$^3$ | <500/mm$^3$ |
| **CHEMISTRIES** | | | | | |
| | Normal Range | Grade 1 | Grade 2 | Grade 3 | Grade 4 |
| Creatinine<br>(μmol/L units used) | 27–62 μmol/L[c]<br>(0.3–0.7 mg/dL) | 62.1 – 91.5 μmol/L<br>(0.7-1.0 mg/dL) | 91.6 – 183 μmol/L<br>(1.1-2.0 mg/dL) | 183.1 – 366 μmol/L<br>(2.1-4.0 mg/dL) | > 366 μmol/L<br>(>4.0 mg/dL) or dialysis required |
| ALT | 3.9 – 49.6 U/L[a] | 49.7-124 U/L | 124.1 – 248 U/L | 248.1 – 496 U/L | > 496 U/L |

[a]Determined and adapted from normal children in Donéguébougou, a rural Malian village and malaria vaccine trial site

[b] From Lugada, E. S. et al. Population-Based Hematologic and Immunologic Reference Values for a Healthy Ugandan Population. *Clin Diag Lab Immunol*, 11, 29-34.
[c] From Johns Hopkins: The Harriet Lane Handbook: A Manual for Pediatric House Officers, 17th edition, 2005, p. 665.

## 10.4 Assessment of causality

Every effort will be made by the investigator to explain each adverse event and assess its causal relationship, if any, to administration of the study vaccine(s).

The degree of certainty with which an adverse event can be attributed to administration of the study vaccine(s) (or alternative causes, e.g., natural history of the underlying diseases, concomitant therapy, etc.) will be determined by how well the event can be understood in terms of one or more of the following:

- Reaction of similar nature having previously been observed with this type of vaccine and/or formulation.
- The event having often been reported in literature for similar types of vaccines.
- The event being temporally associated with vaccination or reproduced on re-vaccination.

All solicited local (injection site) reactions will be considered causally related to vaccination. The investigators will assess the causality of all other adverse events using the following method:

In your opinion, did the vaccine(s) possibly contribute to the adverse event?

NO : The adverse event is not causally related to administration of the study vaccine(s). There are other, more likely causes and administration of the study vaccine(s) is not suspected to have contributed to the adverse event.

YES : There is a reasonable possibility that the vaccine contributed to the adverse event.

If an AE is determined not to be causally related to administration of a study vaccine, the investigators will specify the likely cause if one can be determined.

## 10.5 Following-up of adverse events and assessment of outcome

Investigators will follow up subjects with serious adverse events until the event has resolved or until the condition has stabilized regardless of when this outcome occurred in relation to the study conclusion. Investigators will follow up participants with non-serious adverse events until the participant completes the study. Clinically significant laboratory abnormalities, as well as any adverse event, will be followed up until they have returned to normal, or until a satisfactory

explanation has been provided. Reports relative to the subsequent course of an adverse event noted for any subject will be submitted to the DMID Medical Monitor.

Outcome will be assessed as Resolved without Sequelae, Resolved with Sequelae, Ongoing, or Death.

## 10.6 Clinical malaria

For adverse event reporting, clinical malaria will be recorded in the same fashion as other adverse events. In general, uncomplicated malaria will constitute an AE and severe malaria may constitute an SAE. The formal clinical case definitions of these conditions used for efficacy endpoints will not be used to define adverse events or to determine treatment. Study clinicians may exercise their judgment to hospitalize cases that do not meet standard definitions of severe malaria.

## 10.7 Serious adverse events

In 2004 the under-5 year age group mortality rate in Mali was 21.9%. While the children participating in this study will receive better medical care than most Malian children do, some hospitalizations and even deaths are likely in the normal course of events in this population.

### Definition of a serious adverse event

A serious adverse event is any untoward medical occurrence that results in death, is life threatening, results in persistent or significant disability/incapacity, requires in-patient hospitalization or prolongation of existing hospitalization or is a congenital anomaly/birth defect in the offspring of a study subject. In addition, important medical events that may jeopardize the participant or may require intervention to prevent one of the other outcomes listed above will be considered serious.

- Life threatening—definition: An adverse event is life threatening if the participant was at risk of death at the time of the event; it does not refer to an event, which hypothetically might have caused death, if it were more severe.

- Disabling/incapacitating—definition: An adverse event is incapacitating or disabling if the event results in a substantial disruption of the participant's ability to carry out normal life functions. This definition is not intended to include experiences of relatively minor medical significance such as headache, nausea, vomiting, diarrhea, influenza, and accidental trauma (e.g. sprained ankle).

- Hospitalization: In general, hospitalization signifies that the participant has been detained (usually involving at least an overnight stay) at the hospital or emergency ward for treatment that would not have been appropriate in the physician's office or outpatient setting.

## Reporting serious adverse events

All serious adverse events will be:

- Recorded on the appropriate serious adverse event case report form
- Followed through resolution by a study physician
- Reviewed by a study physician

Any AE considered serious by the Principal Investigator or Subinvestigator or meets the aforementioned criteria must be submitted on an SAE form to PPD Development, NIAID's pharmacovigilance contractor, which will notify USAMMDA as the Sponsor as well as DMID, USAID, GSKBio, and the University of Maryland International Regulatory Affairs Coordinator of any SAEs, with in the timelines required by each of these parties.

### USAMMDA SAE reporting contact information

US Army Medical Research and Materiel Command / US Army Medical Materiel Development Activity Regulatory Affairs Office:

- E-mail USAMRMCREGAFFAIRS@amedd.army.mil (Preferred method)
- facsimile (301-619-0197) or
- telephone (301-619-0317) – Provide voice mail message during non-duty hours

USAMMDA will notify the HSRRB of any SAEs.

### PPD SAE reporting contact information

Medical Affairs/Pharmacovigilance
PPD Development
3151 17th St.
Wilmington, NC 28412
SAE Fax line: 888 488-9697
Email: dmidpvg@wilm.ppdi.com

Questions about SAE reporting may be referred to the SAE Hotline (available 24 hours a day/7 days a week) at 800 201-8725

### GSK SAE reporting contact information

| **Study Contact at GSK Biologicals for Reporting Serious Adverse Events Manager Clinical Safety Vaccines** | **Clinical Development Manager** |
|---|---|
| GSK Biologicals Clinical Safety Physician,<br>GlaxoSmithKline Biologicals,<br>Rue de l'Institut 89, 1330 Rixensart,<br>Belgium.<br>Tel: +32.2.656.87.98 | Amanda Leach,<br>Clinical Development Manager,<br>GlaxoSmithKline Biologicals,<br>Rue de l'Institut 89, 1330 Rixensart,<br>Belgium. |

Fax: +32.2.656.80.09
Mobile phone for 7/7 day availability:
+32.477.40.47.13
email: rix.ct-safety-vac@gskbio.com

Tel: +32.2.656.77.88
Fax: +32.2.656.80.44
email: amanda.leach@gskbio.com

**SAEs will also be reported within 24 hours by telephone, fax or E-mail to the Local Medical Monitor and the FMPOS Ethics Review Committee in Mali.**

**HSRRB SAE reporting contact information**

All unanticipated problems involving risk to subjects or others, serious adverse events related to participation in the study and subject deaths related to participation in the study should be promptly reported by phone (301-619-2165), by email (hsrrb@amedd.army.mil), or by facsimile (301-619-7803) to the USAMRMC, Office of Research Protections, Human Research Protection Office and by phone (301-319-9940), by email (ResearchManagement@na.amedd.army.mil), or by facsimile (301-319-9961) to the Office of Research Management, Walter Reed Army Institute of Research. A complete written report will follow the initial notification. In addition to the methods above, the complete report will be sent to the U.S. Army Medical Research and Materiel Command, ATTN: MCMR-ZB-PH, 504 Scott Street, Fort Detrick, Maryland 21702-5012 and to the Walter Reed Army Institute of Research, Office of Research Management, 503 Robert Grant Avenue, Silver Spring, MD 20910.

**Regulatory reporting**

Following notification via PPD from the investigator, USAMMDA, the IND sponsor, will report events that are both serious and unexpected and that are associated study product(s) to the FDA within the required timelines as specified in 21 CFR 312.32: fatal and life threatening events within 7 calendar days (by phone or fax) and all other serious adverse events in writing within 15 calendar days. All serious events designed as “not associated” to study product(s), will be reported to the FDA at least annually in a summary format.

**SAE reporting procedures**

The study clinician will complete a Serious Adverse Event Form within the following timelines:

- All deaths, whether related or unrelated, will be recorded on the Serious Event Form and sent by fax or electronic mail immediately upon discovery of the death regardless of availability of complete information. For initial reports provided via telephone, a written report via electronic mail or fax will be provided within 24 hours of discovery of death by investigators. Follow-up reports will be provided as complete or additional relevant information is available.

- Serious adverse events other than death, regardless of relationship, will be reported via fax by the site immediately upon discovery of the experience regardless of availability of complete information. For initial reports provided via telephone, provide a written report via electronic mail or facsimile within 72 hours of becoming aware of the event. Follow-up reports will be provided as complete or additional relevant information is available.

Other supporting documentation of the event may be requested by the pharmacovigilance contractor and should be provided as soon as possible.

All SAEs will be followed until satisfactory resolution or until the Principal Investigator or Subinvestigator deems the event to be chronic or the patient to be stable.

Every serious adverse event that is not resolved at the time the initial written report is filed will have a follow-up report submitted when information is available. Any submitted report will be identified as “initial”, “follow-up”, or “medical monitor”.

The initial notification will include:

- The study protocol number and the name of the PI
- The participant ID number, gender and age
- The date of onset of the SAE, and date of administration of study vaccine(s)
- Description of event

The PI will not wait to collect additional information to fully document the event before making notification of a serious adverse event. The telephone/e-mail report will be followed by a full written report using the SAE form within the CRF, detailing relevant aspects of the adverse events in question.

Formal surveillance will continue until stability or resolution of the event. However, in the event that instances of death or cancer in a study participant are brought to the attention of the investigator AT ANY TIME after cessation of the study AND suspected by the Investigators to be related to study medication will be reported to USAMMDA, DMID and GSK within four weeks of coming to the knowledge of the PI.

## 10.8 Treatment of adverse events

The investigators will provide treatment of adverse events. Advice will be sought from the Local Medical Monitor and other medical specialists as indicated for severe, unusual or complicated medical conditions. Treatment events will be fully documented. Clinical trials insurance will cover medical liability of Malian clinical investigators.

## 10.9 Halting rules

Decisions to halt or pause the study will be made by USAMMDA and DMID based on the recommendations of the DSMB. DMID and USAMMDA retain the authority to halt or pause the study based on recommendations of the DSMB. The following criteria will be used as rules to put the study on hold until reviewed by the LMM, DSMB, USAMMDA and DMID. Any one or more of the following will result in pausing the trial for a safety review:

1. One or more participants experience an SAE that is determined to be related to the vaccine.

2. One or more participants experiences systemic allergic reaction (i.e., bronchospasm, allergy-related edema/angiedema, hypotension or anaphylaxis) associated with the vaccine.

3. The incidence of Grade 3 systemic AEs recorded in the seven day follow-up period following any immunization exceeds 20% after at least 50 participants have received that immunization.

If the study is halted or paused, unanimous agreement among USAMMDA, DMID, investigators and partners including WRAIR, USAID and GSK will be required to resume the study.

## 10.10 Safety oversight

### Local Medical Monitor

A qualified Malian pediatrician will serve as the Local Medical Monitor (LMM) for this study. The LMM's *curriculum vitae* will be maintained on record. She is a qualified and experienced pediatrician not otherwise associated with this protocol, who is able to provide medical care to research subjects for conditions that may arise during the conduct of the study. If safety concerns are identified, the LMM may request a meeting of the DSMB to review safety data. The medical monitor is required to review all unanticipated problems involving risk to subjects or others, SAEs, and all subject deaths associated with the protocol and provide an unbiased written report of the event. For SAEs and deaths, she will provide an unbiased written report of the event within 10 calendar days of the initial report. At a minimum, the medical monitor should comment on the outcomes of the event or problem and, in the case of an SAE or death, comment on the relationship to participation in the study. The medical monitor should also indicate whether he/she concurs with the details of the report provided by the study investigator. Reports for events determined by either the investigator or medical monitor to be possibly or definitely related to participation and reports of events resulting in death should be promptly forwarded to the sponsors and the IRBs through the DMID contractor PPD.

The involvement of the LMM will be particularly important when decisions related to safety of participants have to be made quickly. A copy of treatment codes will be in her safekeeping and she may unblind individual study participants if deemed necessary for medical and/or ethical reasons (for example, in the case of a participant in urgent need of rabies immunization). In exceptional circumstances, for example a death possibly related to vaccination, she would have the authority to temporarily suspend the whole or any specific aspect of the trial pending review by the USAMMDA and DMID Medical Monitors and the DSMB.

The LMM will be on-site during active phases of immunization and during the immediate post-vaccination surveillance period. A physician on staff at the Bandiagara Health Center who resides full time in Bandiagara will act as the on-site LMM in support of the LMM between the vaccinations.

The LMM's role will include:

- Acting as the study participants' advocate

- Providing advice to the investigators on whether a set of clinical circumstances in a study warrants formal notification to the DMID and USAMMDA Medical Monitors and the DSMB.

- Providing clinical advice on any illness in study subjects especially in circumstances in which treatment might influence the course of the trial.

- Review all SAEs as outlined above

The LMM will liaise closely with the PI throughout the course of the trial and relay relevant safety information to the PI. The PI and co-PI will subsequently inform the FMPOS IRB, the DSMB, DMID, GSK, WRAIR, USAMMDA and USAID of safety concerns that arise.

## 10.11 Data and safety monitoring board (DSMB)

An independent DSMB will be constituted by DMID to review safety data at the request of the PI, the LMM, USAMMDA or DMID. The role of the DSMB is to provide safety oversight over the conduct of the trial. The DSMB will hold a conference call prior to study initiation, to approve their Charter. The DSMB will be the same as that for the Phase 1 pediatric trial, and has reviewed the safety data generated from the Phase 1 trial, and approved the dose selection for this Phase 2 trial. It will also meet on an ad hoc basis to help evaluate any safety concerns that arise during the immunization phase of the trial. In the event that the DSMB identifies a safety concern, the collaborative group including the investigators, DMID, WRAIR, GSK and USAID will review all safety data to decide whether to give the next sequential dose. Senior investigators and representatives from USAMMDA, DMID, WRAIR, GSK and USAID will be invited to but not obligated to participate in the conference calls during open sessions, which will be followed by closed sessions including only DSMB members.

The investigator will inform the DSMB of:

- All subsequent protocol amendments, informed consent form changes or revisions of other documents originally submitted for review

- Serious adverse events (SAEs) and grade 3 adverse experiences occurring during the study, regardless of relationship to the study vaccine

- New information that may affect adversely the safety of the subjects or the conduct of the study.

A safety reporting website will be established for medical monitors and members of the DSMB to monitor in near-real time all grade 3 or higher SAEs and grade 2 or higher abnormal laboratory test results.

The DSMB may recommend that the Sponsor, USAMMDA, in conjunction with DMID, put the study vaccinations on temporary hold pending review of potential safety issues. The DSMB will request additional information from the Principal Investigator as needed and will request any appropriate

statistical calculations to support discussions with the Sponsor, USAMMDA, in conjunction with DMID. All documentation provided to members of the DSMB for information and review will be treated in a confidential manner.

# 11 CLINICAL MONITORING

## 11.1 Site monitoring plan

Site monitoring will be conducted by DMID or its designated monitoring contractor, to ensure that GCP standards and regulatory guidelines are being followed. Pre-trial monitoring visits will be made to the site, including the clinical laboratory. All records will be made available to monitors, including regulatory files, CRFs and other source documents, QA/QC documentation, SOPs, etc. At the discretion of the monitor, additional site visits may be made during the course of the trial and at the end of the surveillance period.

In addition, monitors from USAMMDA and GSK may make site visits, in coordination with the primary monitoring group designated by DMID. DMID or its designated monitoring contractor and monitors from USAMMDA and GSK should coordinate their visits to reduce the burdens to the clinical study site, clinical investigation staff and study participants.

In conjunction with the monitoring body designated by DMID, a detailed monitoring plan, subject to approval by OCRA, will be developed and included in the Manual of Procedures. The monitoring plan will include the number of subject charts to be reviewed, which/what proportion of data fields and what will be monitored, and who will be responsible for conducting the monitoring visits, and who will be responsible for ensuring that monitoring findings are addressed.

# 12 STATISTICAL CONSIDERATIONS

Data entry will be performed on site and in the MRTC/MMVDU data management unit in Bamako if necessary. The EMMES Corporation will perform data analysis and report primary and secondary endpoint results in collaboration with the MRTC Data Management Unit.

## 12.1 Case definition: Clinical malaria episode

For the primary endpoint, a clinical malaria episode will be defined as symptoms generally consistent with malaria (including but not limited to headache, body aches, fever, chills, weakness), accompanied by documented axillary temperature of 37.5 $^{o}$C or greater, and an asexual *P. falciparum* parasitemia of 2500/mm$^3$ or greater.

## 12.2 Primary endpoints

**Efficacy**

- Time to first clinical malaria episode (as defined above) occurring between randomization and six months after assigned date of the third immunization

**Safety**

- Occurrence of solicited symptoms during a 7-day surveillance period after vaccination (day of vaccination and study days 1, 2, 3 and 7).
- Occurrence of unsolicited symptoms during a 30-day surveillance period after vaccination.
- Occurrence of serious adverse events during the study period.

## 12.3 Secondary endpoints

**Efficacy**

- Time to first clinical malaria episode with parasites with AMA-1 genotypes identical to the 3D7 vaccine strain with respect to designated polymorphic codons
- Incidence density of clinical malaria episodes occurring between randomization and six months after assigned date of the third immunization
- Time to first clinical malaria episode occurring during two years after randomization
- Incidence density of clinical malaria episode occurring during two years after randomization

**Immunogenicity**

- Titers and activity of anti-FMP2.1 antibody at each time point where serology samples are analyzed, measured by ELISA

## 12.4 Exploratory endpoints

Additional exploratory analyses will be done in an effort to refine clinical, parasitological, molecular and immunological assessments of vaccine efficacy and identify surrogate markers of efficacy. These may include:

- Time to first clinical malaria episode using increasingly specific definitions of clinical episodes (parasitemia thresholds of any parasitemia, 100, 1000, 2500, 5000, 10,000, 20,000, 50,000 and 100,000/$mm^3$; and any symptoms consistent with malaria, history of fever) occurring during six months after receipt of the third dose of vaccine
- Asexual *P. falciparum* parasite density measured as area under the curve
- Gametocyte counts measured as area under the curve
- Incidence of anemia (hemoglobin < 8.5 g/dL) and severe anemia (hemoglobin < 5 g/dL)
- Cellular mediated immune (CMI) responses
- Growth inhibition assays (GIA)
- Processing inhibition assays (PIA)
- Microarray analyses of gene expression by PBMCs

Samples for these exploratory assays will be prioritized, and lower priority assays will be conducted only after higher priority assays are completed. The order of priority for each type of sample, in order from highest to lowest priority, will be:

- Serum: Primary ELISA, GIA, antibody avidity, antibody subclass, PIA
- PBMCs: CMI, microarray analyses
- DNA: AMA-1 genotyping, microsatellite analyses, other parasite genomic analyses

## 12.5 Study cohorts/datasets to be evaluated

**Intent to Treat Cohort**

The ‘Intent to Treat Cohort’ will include all participants randomized to one of the two study or control vaccines. The primary study analyses will be based on this cohort.

**Per Protocol Cohort**

The ‘Per Protocol Cohort’ will include all eligible participants randomized to one of the two study or control vaccines, and who received all three assigned vaccinations with the study or control vaccines. Relevant study endpoints will also be analyzed based on this cohort.

**Safety cohort**

The ‘Safety Cohort’ will consist of all participants who have received at least one dose of study vaccine or comparator and for whom any data on safety are available.

The presentation of safety data will explore separately the adverse experiences among participants who received all vaccinations, among those who received only some and among those with protocol deviations.

**Immunogenicity cohort**

The ‘Immunogenicity Cohort’ will include all evaluable participants (i.e., those meeting all eligibility criteria, and who have received at least one immunization with any of the study or control vaccines) for whom data concerning immunogenicity endpoint measures are available. This will include participants for whom assay results are available for antibodies against at least one study vaccine antigen component after vaccination.

## 12.6 Sample size considerations

The sample size is motivated by the time to first clinical malaria episode occurring between randomization and six months after the assigned date of the third immunization. The power calculations based on the Intent to Treat analysis and Per Protocol analysis are expected to be similar; the malaria attack rate during the two months between randomization and the third vaccination is thought be relatively small as this precedes the start of malaria transmission season. Further, the anticipated losses to follow-up are also expected to be very small during this interval.

The following power considerations are based on a six month malaria incidence rate of 0.75 in the control arm; this is appropriate providing that the third vaccination is administered to participants just prior to the beginning of malaria transmission season as malaria events are assumed to occur during these 6 months of the year. It is assumed that losses of follow-up due to death, withdrawal or drop out will occur in up to 15% of the sample. For the calculation, the log-rank statistic for time to event data is used. We assume that the risk ratio between the two treatment groups remains constant over the six months follow-up.

Using these six-month rates for losses to follow-up and malaria incidence, we assume an exponential distribution with a constant hazard rate of 1.38 in the control arm. The table below provides various sample size scenarios for the log-rank statistic with a one-sided hypothesis at the 0.025 significance level and 90% power. A total sample size of 83 participants is required to detect a 50% difference in the attack rates. To detect a difference such that the hazard rate in the

treatment group is 50% lower than the hazard rate in the control arm, a total of 160 participants are required. A sample size of 398 is adequate to detect a hazard rate of 0.92 in the treatment arm during the unit study period (ie attack rates of .75 for control versus .4 for vaccine arm).

| Control Arm 6 mo. Malaria Attack Rate | Treatment Arm 6 mo. Malaria Attack Rate | Minimum Required Sample Size (TOTAL) |
|---|---|---|
| 0.75 | 0.38 | 82 |
| | 0.50 | 160 |
| | 0.55 | 238 |
| | 0.60 | 398 |

(Lachin, John M. and Foulkes, Mary A. 1986. 'Evaluation of Sample Size and Power for Analyses of Survival with Allowance for Nonuniform Patient Entry, Losses to Follow-up, Noncompliance, and Stratification', Biometrics, Volume 42, September, pages 507-516) as implemented in PASS 2005, NCSS.com.

This sample size of 398 was selected for several reasons:

1) It is possible that attack rates will be lower than measured in our previous studies due to factors such as increased use of bednets, improved prevention measures or use of more highly efficacious antimalarial drugs relative to when malaria incidence was last measured at this site. If the malaria attack rate is halved, then the study power would be 88% to detect the same absolute difference (attack rates of .375 for control and .225 for the experimental vaccine). Under this scenario, the vaccine decreases the event rate 40% (.15/.375). Larger improvements, while possible, seem unlikely given prior experiences with candidate malaria vaccines.

2) Any operational disruptions that cause a delay in vaccine administration would shorten the post-vaccination portion of the malaria season and decrease the length of the follow-up period during which events will occur. The selected sample size would be able tolerate delays of about 6 weeks and still maintain 80% power.

3) A secondary interest is the examination of attack rates for various genotype groupings, in particular those homologous to the AMA1 genotypes for the 3D7 cloned vaccine source. While underpowered for infection from this genotype group alone, a study with 200 control subjects could result in 15-25 infections. Thus a highly protective vaccine against infection from this genotype group might be identified. Smaller studies would limit the number of potential cases of this type.

## 12.7 Factors that may impact malaria risk

### Age and gender

The most important determinant of risk of clinical malaria in this and other endemic settings is age, as a marker for acquired immunity (54;57). For this reason, participants will be randomized to receive malaria vaccine or rabies vaccine with stratification for age by two-year increments (1-2 years, 3-4 years, 5-6 years). In this setting there is no evidence that gender is associated with malaria risk, so randomization will not be stratified by gender.

### Insecticide treated nets

Insecticide treated nets (ITN), which are locally available and which study participants will be encouraged to use, are expected to affect malaria risk. However, reported ITN use at the time of randomization, which will occur at the end of the dry season when mosquitoes are absent, is not a reliable predictor of subsequent use during the efficacy surveillance period, during which malaria transmission will rise and peak. Therefore at each scheduled monthly visit and at the 12, 18 and 24 months visits, reported use of ITN will be recorded. Use of ITN will be treated as a time-varying covariate in the Cox proportional hazards model. Briefly, at each time point where a clinical malaria episode occurs, vaccine effect will be examined separately among users and nonusers of ITN based on the last reported ITN use. The estimate of effect will be a combined estimate across these two groups.

### Residence

Over the course of the malaria season, the entomological inoculation rates vary as much as two-fold among the three main sectors of Bandiagara town. However, more detailed patterns of infection rate differences within the town are not known, and to the extent that malaria risk is heterogeneous, it is likely that this heterogeneity exists at the level of individual household compounds. It will not be possible only to recruit from households with specified numbers of eligible children greater than one and randomize within households, since many households may have only one eligible child. It is also not feasible to stratify by quartier (eight well-defined sections of town) because recruitment of specific number of children from each age stratum cannot be predicted by quartier. Therefore to partially account for the possibility of heterogeneous risk of malaria based on residence, quartier of residence will be recorded and treated as a covariate in the analysis. However, it is recognized that heterogeneity of malaria risk very likely exists within quartiers, and therefore randomization must be relied on to distribute this risk relatively evenly among age strata and intervention groups.

### Malaria treatment

As with other medical conditions that arise, drug treatment for malaria will be based on standard local treatment recommendations. Uncomplicated and severe malaria will be treated according to antimalarial drug policy in Mali. Presently artesunate-amodiaquine is recommended for treating uncomplicated malaria and parenteral quinine for severe malaria. Should these recommendations

change, treatments will follow policy. All malaria drug treatments will be recorded and treated as a time-varying covariate in the Cox proportional hazards model in a fashion similar to that described above for ITN.

## 12.8 Final analysis plan

A final research analysis plan will be agreed upon by the investigators, USAMMDA, DMID, WRAIR, GSK and USAID prior to locking of the database for the final analysis. The primary analysis will be conducted on data and samples collected until Day 240 (six months post Immunization 3 for each cohort). The primary analysis will be used for decision-making related to the product clinical development plan. The study will continue in a single blind manner for additional safety surveillance, and prolonged efficacy and immunogenicity assessment. This additional information will be appended to the study report. It is anticipated that the results of this study will be presented to the scientific community via oral presentations at meetings and written publications in scientific journals. The data to be presented and the authorship will be discussed between partners prior to any official communication.

The official report of the primary analysis will be written by the study investigators and the statistical consultant, reviewed by all partners, and submitted through appropriate channels for approval by USAMMDA and DMID. This report will contain detailed information about the participants, their tolerance of the vaccines, their side effects and laboratory abnormalities, as well as their overall immune responses to immunization.

### Analysis of demographics

Demographic characteristics (age, gender, and quartier of residence) of each study cohort will be tabulated. The mean age (plus range and standard deviation) by sex of the enrolled participants, as a whole and per group will be tabulated.

### Analysis of efficacy

The time to first clinical malaria episode occurring from randomization through six months after the assigned date of the third dose of vaccine will be analyzed using survival methods, including Kaplan-Meier estimates and Cox proportional hazards models. The proportionality assumption will be graphically assessed.

### Analysis of allele-specific efficacy

Time to first infection with parasites with AMA-1 genotypes identical to the 3D7 vaccine strain with respect to a set of key polymorphic codons will also be analyzed. These outcomes will be analyzed within the intent to treat cohort and per protocol cohorts.

The parasite DNA from filter paper samples corresponding to clinical malaria episodes and asymptomatic infections will be subject to sequencing of the full-length *ama-1* gene. Allele-specific

efficacy will be measured for time to first clinical malaria episodes with 3D7 genotype. Reduced frequency compared to baseline of 3D7 genotypes among all clinical episodes and among all clinical and asymptomatic infections will be compared between malaria vaccine and control groups as secondary endpoints for allele-specific efficacy. These will be analyzed for the period from days 60 to 240 (corresponding to the first post-immunization malaria season) in the primary analysis, and also stratified by study time period (days 60-90, 90-180, 181-272, 273-364, 365-730) to assess the duration of any allele-specific efficacy over time. In addition, an analysis of all episodes occurring after the first immunization will be done.

Studies to identify the AMA-1 polymorphic amino acid residues most associated with risk of clinical malaria episodes and with allele-specific in vitro growth inhibition are currently underway (S. Takala, S. Dutta, unpublished). The results of these studies, which will be available before the trial is finished, will be used to guide the choice of specific amino acid residues, and sets of residues, for measures of allele-specific efficacy. It is anticipated that haplotypes based on a limited number of key residues will be identified with the result that the frequency of haplotypes identical to 3D7 with respect to these key residues will be high enough to have an adequate sample size to detect allele-specific efficacy.

To measure potential vaccine-induced selection at the level of individual codons, sequences will be compared from 1) samples collected before and after vaccination, and 2) samples from infections occurring on or after study day 60 in the vaccine and control group using two complementary approaches: (1) we will calculate an $\omega$ per codon (rate of synonymous versus non-synonymous base on a codon base model) as implemented in MrBayes and PALM; (2) we will calculate Pn (probability of a non-synonymous substitution) per codon using a new class of hierarchical generalized linear models (GLMs) that allows maximum flexibility in modeling rate heterogeneity and explicitly models parameter uncertainties using Bayesian estimation (Merl, Prado, and Escalante in preparation).

We will also measure the divergence of AMA-1 sequences from the vaccine strain in both vaccinated and control groups. This will be done by estimating heterozygosity of full-length *ama-1* sequences by the parameter $\pi$ (58), using bootstrap methods to simulate the distribution of $\pi$ in order to calculate confidence intervals and test for significant differences in heterozygosity between pre- and post-immunization time points for both vaccinated and control groups. Sequence divergence between *ama-1* sequences and the 3D7 strain will be calculated and compared between the time points. These analyses will be conducted using MEGA3 (59)

### Analysis of immunogenicity

The primary immunogenicity endpoint of ELISA titers of FMP2.1 antibodies will be assessed in several ways. A series of graphs will display immunologic responses. For each vaccine group and time point, the distribution of anti-FMP2.1 antibody levels and reverse cumulative distribution curves will be plotted. Corresponding summary statistics will show means and standard deviations, as well as median, 25th and 75th percentiles, and 10th and 90th percentiles. The results will be presented both as raw data and as log-transformed data.

In addition, for each treatment group and time point, anti-FMP2.1 antibody levels will be presented as geometric means of OD units with 95% confidence intervals. For each vaccine group and for each time point, a table will show the proportion of participants with two-fold, four-fold, and eight-fold increases in anti-FMP2.1 antibody titers relative to their pre-immunization titers.

### Analysis of safety

The overall percentage of participants with at least one local adverse event (solicited or unsolicited) and the percentage with at least one general adverse event (solicited and unsolicited) during the 7-day surveillance period after vaccination will be tabulated. The incidence, intensity and relationship of individual solicited symptoms to the vaccine over the 7-day surveillance period will be calculated per group and vaccine administration.

The number of participants with at least one report of an unsolicited adverse event, classified using MedDRA System Organ Classes and Preferred Terms, reported up to 30 days after vaccination will be tabulated per group and vaccine administration. The intensity and relationship to vaccination of the unsolicited adverse events reported will also be assessed.

Serious adverse events will be described. Comparisons between study groups of incidence of symptoms, local and general symptoms will be made based on a two-sided Fisher's Exact Tests. Analysis of safety during the 15-month surveillance period will consist of comparison of incidence of serious adverse events, as well as hemoglobin, creatinine and ALT levels.

**Clinical laboratory parameters**

Hematological (CBC) and biochemical (ALT, creatinine) laboratory parameters will be measured at specific time points, Days 0, 7, 30, 37, 60, 67 and 90. Clinically relevant abnormal values based on reference intervals determined in rural Malian children will be tabulated and a trend analysis could be performed if deemed necessary. Laboratory abnormalities will be graded on a scale of 1-4 as defined in relevant SOPs.

### Analysis of CMI responses

Correlation will be measured 1) between serum anti-AMA-1 antibody levels and T-helper cell responses; and 2) between avidity and/or classes and IgG sub-classes of anti-AMA-1 antibodies and T-helper cell responses. T-helper cell responses will be assessed by lymphoproliferative responses to AMA-1 antigenic stimulation, IFN-γ and IL-5 production as measured by ELISpot and lastly, by flow cytometric methods dependent upon PBMC availability. Correlations between continuous measures of immune response will be assessed using the standard Pearson as well as Spearman rank correlation coefficients, on $\log_{10}$ transformed data when a logarithmic transformation results in a distribution more nearly normal than the distribution of untransformed values. Association between a continuous and a categorical measure will be assessed using t-tests or analysis of variance, or the analogous tests on ranks. Association between categorical measures will be assessed using chi-square or exact tests. Appropriate summary descriptive statistics (e.g.,

means and standard deviations or medians and ranges) will be presented. Effects of covariates (e.g., gender, ethnicity) will be assessed using regression models.

# 13 SOURCE DOCUMENTS AND ACCESS TO SOURCE DATA/DOCUMENTS

Electronic CRFs (eCRFs) will be supplied by the EMMES Corporation under contract to the NIAID, and a remote data entry system will be used. Copies of the CRFs will be made available for Source Document Workbooks (SDWs). The SDW for each subject will be maintained at the site. All SDWs will be filled out completely by appropriate study personnel.

The site will permit authorized representatives of the USAMMDA and DMID, their designees, and appropriate regulatory agencies to examine (and when required by applicable law, to copy) clinical records for the purposes of quality assurance reviews, audits and evaluation of the study safety and progress. These representatives will be permitted access to all source data.

# 14 QUALITY CONTROL AND QUALITY ASSURANCE

SOPs for quality management will be developed, used to train appropriate personnel, and kept on file with documentation of training. Data will be evaluated for compliance with protocol and accuracy in relation to source documents. The study will be conducted in accordance with procedures identified in the protocol. The types of materials to be reviewed, who is responsible, and the schedule for reviews will be referenced in the SOPs. Study-specific training will be provided for all staff prior to the commencement of the trial.

The study will be conducted at a single center, the Bandiagara Malaria Project in Bandiagara, Mali, with the exception of immunological studies which will be conducted at WRAIR (ELISA, GIA, PIA), UMB (antibody avidity, CMI, sequencing) and the main campus of the University of Bamako (serology not to be used for study endpoints, and CMI).

SOPs will be used at all clinical and laboratory sites. Regular monitoring and an independent audit will be performed according to GCP/ICH (e.g., data monitoring). Following written standard operating procedures, the monitors will verify that the clinical trial is conducted and data are generated, documented (recorded), and reported in compliance with the protocol, GCP, and the applicable regulatory requirements. Reports will be submitted to DMID and USAMMDA on monitoring activities.

The investigational site will provide direct access to all trial related sites, source data/documents, and reports for the purpose of monitoring and auditing by the USAMMDA and DMID, and inspection by local and regulatory authorities.

The EMMES Corporation and the MRTC Biostatistics and Epidemiology Unit will implement quality control procedures beginning with the data entry system and generate data quality control checks that will be run on the database. Any missing data or data anomalies will be communicated to the field site for clarification/resolution.

# 15 ETHICS/PROTECTION OF HUMAN SUBJECTS

## 15.1 Ethical standard

The WRAIR FMP2.1/AS02A vaccine has been submitted as FDA IND BB-11,140 and the study described in this protocol will be conducted according to current Good Clinical Practices (US 21 CFR Part 50-Protection of Human Subjects and Part 56-Institutional Review Boards, US Army Regulation AR 40-38 and AR 70-25, US 45 CFR 46, 21 CFR 312, the Declaration of Helsinki, and the applicable rules and regulations of Mali).

The FMPOS IRB (FWA00001769) will review and approve the protocol prior to study start. In addition, the study will be reviewed by the WRAIR HURC, by DMID, and the University of Maryland IRB. Documentation of the approval by these ethical review boards will be kept in the PI's study file.

## 15.2 Institutional review board

All amendments will be submitted to the University of Bamako Faculty of Medicine Pharmacy and Odonto-stomatology (FMPOS) IRB, the UMB IRB, and the HSSRB as well as to USAMMDA and DMID. No amendments will go into effect without written approval from the FMPOS IRB, the UMD IRB, WRAIR HURC, USAMMDA and DMID except when necessary to eliminate immediate hazards to the participants. Protocol deviations will also be reported to each IRB according to the policy of each IRB. CRFs and other source documents will be examined to determine whether missing data were not transcribed, unavailable or missing for unknown reasons and this information will be coded and documented in the database.

The investigators will inform all the IRBs, USAMMDA and DMID of the following:

- All subsequent protocol amendments, informed consent changes or revisions of other documents originally submitted for review
- Serious and/or unexpected adverse events occurring during the study, where required
- New information, including any provided by GSK, USAMMDA or WRAIR, that may affect adversely the safety of the participants or the conduct of the study
- An annual update and/or continuing review reports, as required
- A final report including SAE outcomes will be provided when the study has been completed.

## 15.3 Informed consent process

The principles of informed consent in the current edition of the Declaration of Helsinki will be implemented before any protocol-specified procedures or interventions are carried out.

Information will be given in both oral and written form whenever possible. The written consent documents will embody the elements of informed consent as described in the current edition of the Declaration of Helsinki, will adhere to the ICH Harmonised Tripartite Guideline for Good Clinical Practice and 45CFR46 and 21CFR50, and will also comply with applicable Malian regulations. The oral consent process will be consistent with 45CFR46.§46.117, 21CFR50.27 and ICH E6 (R1) Section 4.8. Independent witnesses will be used to attest that illiterate potential participants have understood the contents of the informed consent document.

### Screening and study informed consent

We have published detailed descriptions of the processes we use to obtain community "permission to enter" and individual informed consent (60). We consider informed consent to be a dynamic, ongoing process, with continuous availability of investigators to answer any questions that arise in the course of the trial and to ensure that participants' parents/guardians understand trial procedures. Should new data become available that could affect participant safety and/or willingness to continue in the study, informed consent would be obtained and documented again.

The extensive contact between the team of investigators and the population of Bandiagara has led to the development of mutual trust and the establishment of an ongoing informed consent process attempting to address issues related to interventional studies in resource-limited settings. Many discussions with local community leaders, heads of families and citizens through group meetings, and more limited group interviews have reviewed the need to obtain a written informed consent from parents/guardians. The community has now become familiar with the informed consent process, including written, signed consent forms, which have been used for several studies at this site, including several interventional and observational pediatric trials, two Phase 1 adult malaria vaccine trials in 2003-2005 and a Phase 1 pediatric trial of this vaccine in 2006-2007.

Before starting any study in Bandiagara, the senior Malian and US investigators visit the local commandant (representative of the national government), the mayor, the director of the local school system, the chiefs of each of the eight quartiers of Bandiagara, the medical director of the local health center, the director of the Bandiagara Center for Research of Traditional Medicine, and the head of the Bandiagara traditional healers' association. These are courtesy visits during which results of the previous year's studies are summarized and plans for new studies are explained and any questions are answered. In accordance with the tradition in Mali, small quantities of kola nuts are given to the chiefs of the quartiers and the traditional healers as a sign of respect.

These individual meetings are followed by a larger community meeting attended by the above personages as well as numerous other local health care providers, traditional healers and notable citizens (including several respected women from the community). Planned studies are explained in more detail, and ample time is given for carefully and thoroughly addressing all questions and

concerns. This question and answer period is frequently prolonged with many detailed and often sophisticated questions being raised. Each presentation, question and response is translated from French into Dogon and Peulh so that all present understand the entire discourse.

Once this group of community leaders has expressed their approval of the planned study, they disseminate information to their various constituencies, so that when potential recruits approach the study staff they are already generally aware of the nature of the impending study. The investigators do not consider this process to constitute “community consent” in addition to or in lieu of individual informed consent, but rather a community “permission to enter” that is a necessary prerequisite to conducting any study in a tight-knit and highly organized traditional rural community such as Bandiagara.

After community meetings have been held, a brief announcement is made on local radio describing the study. Because consent must be obtained in local languages and dialects that are not written, prior to initiating screening and obtaining informed consent, the study team holds sessions to review the oral translation of the consent forms into the relevant local languages and dialects word by word, until there is consensus that the individuals responsible for giving consent in each language are conveying as accurately as possible the exact content of the IRB-approved French language consent form. These sessions are led by senior investigators and include all clinical co-investigators who will be administering informed consent, as well as local guides who are fluent in all of the local languages and dialects. The French version of the consent form is orally translated into local languages and dialects repeatedly until all investigators reach consensus on the oral translations.

Adequate time is allotted for screening and recruitment to allow plenty of time for participants’ parents/guardians to consider their decision about participating and to discuss their participation with family members and others in the community. At the times of screening and recruitment, the consent forms are read to parents/guardians who speak French, and translated orally into the language of choice of each parents/guardians. Dogon is the main language spoken in Bandiagara. It has no written form and is constituted of more than 12 distinct dialects. Other languages used in the area include Bambara and Fulani, both of which do have written forms, although neither the local population nor the study staff use the written forms of these mainly oral languages. Most of the population is illiterate; therefore, with the exception of those whose preferred language is French, informed consent will be administered by oral translation of the text in presence of a witness. In all cases, the investigator will give the parents/guardians ample opportunity to inquire about the details of the study and to ask any questions before dating and signing the consent forms, including the opportunity to take a copy of the consent form home to review with family members or others before returning on a later day with their decision. All illiterate individuals will have the study and consent forms explained to them point by point by the interviewer in the presence of a witness who will sign the consent form. Witnesses will have no association with the conduct of the study and will not be related to the study subject. Witnesses receive training that emphasizes protection of confidentiality, and assist with ensuring full and accurate oral translation of the information on the consent forms. Individual consent to participate in research studies is given freely, and is not subject to approval by village elders or others.

We will use a comprehension test developed for previous malaria vaccine trials at this site. Parents of participants must answer all questions on the comprehension exam correctly to be eligible for enrollment in the study. Study staff will use incorrect answers to identify those areas of the informed consent document that need further review with the person giving informed consent. The incorrectly answered questions will then be repeated to the participant after a review of the informed consent form. A final score of 100% will be required for the participant to be considered eligible for enrollment in the study. The test may be taken a maximum of four times. This test will be administered orally in the presence of a witness in the case of potential participants who cannot read.

Informed consent will be documented by the use of a written consent form approved by the IRBs and signed or thumbprinted and dated by the parent/guardian, and by the person who conducted the informed consent discussion. Thumb printing will be used for illiterate persons, who are expected to constitute the majority of parents/guardians. The consent form will be orally translated into native languages from the French written version of the consent form. Consent will be administered by a study clinician who is fluent in French and will either be fluent in the local language of the parents/guardians or use a translator. A witness will assist during the procedure. After the participant's parent/guardian clearly states that she/he has understood what was explained and agrees for his/her child to participate in the study, the consent forms will be completed. The parent will be asked if she/he prefers to thumbprint or to sign. In the case of the thumbprint option, the distal end of her/his left thumb will be applied to a stamp inker and then firmly applied to the space on the consent forms reserved for thumbprints. This procedure has been followed for many years by the BMP team, and thumbprints obtained by trained staff following an SOP are uniformly legible.

The signature/thumbprint confirms that the consent is based on information that has been understood. Each participant's parents/guardians' signed informed consent form is kept on file by the investigator for possible inspection by regulatory authorities. The subject will receive a copy of the signed and dated written informed consent forms and any other written information provided by the investigator, and will receive copies of any signed and dated consent form updates and any amendments to the written information.

Since the vast majority of study participants' parents/guardians do not use telephones, fax or mail, contact information is provided in terms of local physicians who can be visited directly and who can themselves reach the investigators directly or by telephone or fax.

### Screening recruitment radio announcement text

**"**The Bandiagara Research Project team from the Faculty of Medicine in Bamako has returned to Bandiagara, and sends its greetings to the population of Bandiagara. The team is here to test an experimental malaria vaccine, to see if it prevents malaria in children. Parents of boys and girls aged 1-6 years who live in Bandiagara town and are interested in having their child participate in this research study are invited to come to the Bandiagara Health Center at [*time*] on [*date*] to learn more about this study**."**

### Compensation

To compensate the participant's family for participating in the study, each will be given 100 kg rice and 100 kg millet, worth about $120. They will receive half of the rice and millet after the first shot and the other half at the end of the study. Participants who withdraw from the study after receiving at least one immunization but before the study is completed will receive a portion of the second portion prorated according to the time they remain in the study. The annual per capita income for Mali as a whole is estimated to be $250, with a more recent estimate of $600. However, these figures are based on large segments of the population who live in rural areas with virtually no cash economy, and per capita income in Bandiagara is higher than this. Income data specifically for Bandiagara are not available, but Bandiagara is a relatively affluent town by Malian standards, with a lively market economy and a significant tourist trade. As the primary IRB for our studies in Bandiagara since 1997, the Mali IRB reviews and sets appropriate compensation amounts for our studies and for studies in other specific settings in Mali, based on local considerations with the aim of providing adequate but non-coercive compensation for time away from work and for accepting risks of study participation.

## 15.4 Subject confidentiality

Subject confidentiality is held strictly in trust by the participating investigators, their staff, DMID and USAMMDA and their agents. Confidentiality is extended to cover testing of biological samples and genetic tests in addition to the clinical information relating to participating subjects.

The study protocol, documentation, data, and all other information generated will be held in strict confidence. No information concerning the study or the data will be released to any unauthorized third party without prior written approval of USAMMDA and DMID.

The study monitor or other authorized representatives of USAMMDA and DMID may inspect all documents and records required to be maintained by the investigator, including but not limited to, medical records (office, clinic, or hospital) and pharmacy records for the subjects in this study. The clinical study site will permit access to such records.

Participants will be assigned a unique participant ID number. All results will be keyed to this number. Study records will only be available to staff members and will be kept locked at the study site conforming to the investigators' SOPs. Following the conclusion of the study, all records will be maintained on site for a minimum of two years, after which they will be stored long-term in the MMVDU data storage facilities in Bamako. All records will be retained in locked metal boxes for at least two years after a marketing application is approved for FMP2.1; or, if an application is not approved for FMP2.1, until two years after shipment and delivery of the drug for investigational use is discontinued and the FDA has been so notified. After either of these conditions has been met with permission of USAMMDA and DMID, records will be destroyed. Representatives of the US Army Medical Research and Materiel Command (USAMRMC), the FDA, USAMMDA and DMID may review these records.

## 15.5 Study discontinuation

In the event that the study is discontinued before all three doses of the immunizations are administered, completion of the rabies vaccine series will be offered to participants who received one or more doses of the rabies vaccine. Depending on the reason for study discontinuation, rabies vaccine may be offered to participants who received one or more doses of the FMP2.1/AS02A malaria vaccine. If it is felt by the investigators and DMID and/or USAMMDA that immunization with rabies vaccine may interfere with further safety assessment, rabies vaccine may be deferred indefinitely for any individual in the malaria vaccine group.

At the end of the study all participants will be informed through their parents/guardians of the vaccine they received. Participants randomized to the FMP2.1/AS02A vaccine, including any who withdrew early from the study, will be offered rabies immunization at that time. These immunizations will be done at the recommended schedule of 0, 7 and 21 days.

## 15.6 Future use of stored specimens

If residual sera and cells are available following the serological and CMI assays described in this protocol, additional immunological and in vitro studies may be performed at the University of Bamako, University of Maryland or WRAIR on those samples for which permission was expressly granted for preserving samples for future studies at the time of informed consent at study enrollment. These assays may include anti-AMA-1 epitope mapping, determination of response to other allelic forms of the AMA-1 gene, or the ability of participant sera to interfere with in vitro parasite growth or invasion in an antigen-specific fashion. Additional research questions to be asked for cells include antigen-specific cytokine induction as measured by ELISPOT, flow cytometry, or both, or additional analysis to determine specificity of lymphoproliferation responses to this particular allele of AMA-1. These immunological studies will be limited to immune responses to malaria antigens unless specific permission for additional studies is obtained from the relevant IRBs. Samples from participants whose parents/guardians did not grant permission to preserve samples will be discarded after the primary and secondary analyses described in this protocol have been completed. Study participants will have the right to withdraw their permission for further use of their samples at any time during and after the study.

## 15.7 Justification for conducting the study in children

Infants and young children are the target population for a malaria vaccine in Africa, as they bear the main burden of malaria disease and death. Older children and adults living in areas of heavy transmission, such as Bandiagara, Mali, gradually acquire partial immunity that protects them against the clinical manifestations of malaria infection. This Phase 2 trial in children is an essential step toward the Phase 3 pivotal efficacy trials in the target population of infants and young children. Malaria vaccine efficacy cannot be tested in older children or adults in this setting because of their partial immunity. The vaccine was tested first in adults mainly for safety reasons. This clinical development pathway follows that of other malaria vaccines that have been tested in several other

African countries, most notably the RTS,S vaccine that underwent Phase 1 and 2 pediatric trials in Mozambique (9;10).

## 15.8 Justification for exclusion

The study design evaluates a pediatric population. Therefore adults, including women, are excluded.

# 16 DATA HANDLING AND RECORD KEEPING

The investigator is responsible to ensure the accuracy, completeness, legibility, and timeliness of the data reported. All source documents should be completed in a neat, legible manner to ensure accurate interpretation of data. Black ink is required to ensure clarity of reproduced copies. When making changes or corrections, cross out the original entry with a single line, and initial and date the change. DO NOT ERASE, OVERWRITE, OR USE CORRECTION FLUID OR TAPE ON THE ORIGINAL.

Source documents derived from the eCRF will be provided and maintained for recording data for each subject enrolled in the study. Data reported in the eCRF derived from source documents should be consistent with the source documents or the discrepancies should be explained.

DMID and/or its designee will provide guidance to investigators on making corrections to the source documents and eCRF.

A copy of the cleaned and locked eCRF will be provided to DMID and USAMMDA at the end of the study.

## 16.1 Data management responsibilities

All source documents and laboratory reports must be reviewed by the clinical team and data entry staff, who will ensure that they are accurate and complete. Adverse events must be graded, assessed for severity and causality, and reviewed by the site principal investigator or designee.

Data collection is the responsibility of the clinical trial staff at the site under the supervision of the principal investigator. During the study, the investigator must maintain complete and accurate documentation for the study.

The EMMES Corporation will serve as the Statistical and Data Coordinating Center for this study and, in collaboration with the MRTC data management unit, will be responsible for data management, quality review, analysis, and reporting of the study data.

## 16.2 Data capture methods

Clinical data (including AEs, concomitant medications, and reactogenicity data) and clinical laboratory data will be entered into a 21 CFR Part 11-compliant Internet Data Entry System (IDES) provided by The EMMES Corporation. The data system includes password protection and internal quality checks, such as automatic range checks, to identify data that appear inconsistent, incomplete, or inaccurate. Clinical data will be entered directly from the source documents.

## 16.3 Types of data

Data for this study will include safety and outcome measures (e.g., reactogenicity, immunogenicity, efficacy).

## 16.4 Study records retention

Study documents should be retained for a minimum of 2 years after the last approval of a marketing application in an ICH region and until there are no pending or contemplated marketing applications in an ICH region or at least 2 years have elapsed since the formal discontinuation of clinical development of the investigational product. These documents should be retained for a longer period, however, if required by local regulations. No records will be destroyed without the written consent of USAMMDA and DMID, if applicable. It is the responsibility of USAMMDA and DMID to inform the investigator when these documents no longer need to be retained.

## 16.5 Protocol deviations

A protocol deviation is any noncompliance with the clinical trial protocol, GCP, or MOP requirements. The noncompliance may be either on the part of the subject, the investigator, or the study site staff. As a result of deviations, corrective actions are to be developed by the site and implemented promptly.

These practices are consistent with ICH E6:

4.5 Compliance with Protocol, sections 4.5.1, 4.5.2, and 4.5.3

5.1 Quality Assurance and Quality Control, section 5.1.1

5.20 Noncompliance, sections 5.20.1 and 5.20.2.

It is the responsibility of the site to use continuous vigilance to identify and report deviations within 5 working days of identification of the protocol deviation, or within 5 working days of the scheduled protocol-required activity. All deviations must be reported promptly to DMID and USAMMDA via The EMMES Corporation's IDES.

Any deviations that impact subject safety, or that alter the risk to benefit analysis or the scientific integrity of the study will be reported to the DMID, USAMMDA and the IRBs within two business days of the PI or study personnel becoming aware of the deviation.

All deviations from the protocol must be addressed in study subject source documents. A completed copy of the DMID Protocol Deviation Form (IDES form) must be maintained in the regulatory file, as well as in the subject's source document. Protocol deviations must be sent to the local IRB per their guidelines. The site principal investigator/study staff is responsible for knowing and adhering to their IRB requirements.

# 17 OBLIGATIONS TO THE USAMRMC, OFFICE OF RESEARCH PROTECTIONS (ORP), HUMAN RESEARCH PROTECTION OFFICE (HRPO) AND WALTER REED ARMY INSTITUTE OF RESEARCH HUMAN USE REVIEW COMMITTEE

The following are reporting requirements and responsibilities of the Principal Investigator to the United States Army Medical Research and Materiel Command's (USAMRMC) Office of Research Protections (ORP), Human Research Protection Office (HRPO) and the Walter Reed Army Institute of Research Human Use Review Committee:

(a) The protocol will be conducted in accordance with the protocol submitted to and approved by the USAMRMC ORP HRPO and the Walter Reed Army Institute of Research Human Use Review Committee and will not be initiated until written notification of approval of the research project is issued by the USAMRMC ORP HRPO and the Commander of the Walter Reed Army Institute of Research.

(b) Accurate and complete study records will be maintained and made available to representatives of the U.S. Army Medical Research and Materiel Command as a part of their responsibility to protect human subjects in research. Research records will be stored in a confidential manner so as to protect the confidentiality of subject information.

(c) All unanticipated problems involving risk to subjects or others, serious adverse events related to participation in the study and subject deaths related to participation in the study should be promptly reported by phone (301-619-2165), by email (hsrrb@amedd.army.mil), or by facsimile (301-619-7803) to the USAMRMC, Office of Research Protections, Human Research Protection Office and by phone (301-319-9940), by email (ResearchManagement@na.amedd.army.mil), or by facsimile (301-319-9961) to the Office of Research Management, Walter Reed Army Institute of Research. A complete written report will follow the initial notification. In addition to the methods above, the complete report will be sent to the U.S. Army Medical Research and Materiel Command, ATTN: MCMR-ZB-PH, 504 Scott Street, Fort Detrick, Maryland 21702-5012 and to the Walter Reed Army Institute of Research, Office of Research Management, 503 Robert Grant Avenue, Silver Spring, MD 20910.

(d) Any deviation to the protocol that may have an effect on the safety or rights of the subject or the integrity of the study must be reported to the USAMRMC ORP HRPO and the WRAIR HURC as soon as the deviation is identified.

(e) Major modifications to the research protocol and any modifications that could potentially increase risk to subjects must be submitted to the USAMRMC ORP HRPO for approval prior to implementation. All other amendments will be submitted with the continuing review report to the USAMRMC ORP HRPO for acceptance. All modifications will be submitted to the WRAIR HURC for review and approval prior to implementation.

(f) A copy of the continuing review report and the local IRB approval notification will be submitted to the WRAIR HURC for review and approval. A copy of the HURC-approved continuing review report and the local IRB approval notification will be submitted to the USAMRMC ORP HRPO as soon as these documents become available. The final study report and the local IRB notification will be submitted to the WRAIR HURC for review and approval. A copy of the HURC-approved final study report and local IRB approval notification will be submitted to the USAMRMC ORP HRPO as soon as these documents become available.

(g) The knowledge of any pending compliance inspection/visit by the FDA, OHRP, or other government agency concerning clinical investigation or research, the issuance of Inspection Reports, FDA Form 483, warning letters or actions taken by any Regulatory Agencies including legal or medical actions and any instances of serious or continuing noncompliance with the regulations or requirements will be reported immediately to USAMRMC ORP HRPO and WRAIR HURC.

(h) Responsibilities of the Medical Monitor

The medical monitor is required to review all unanticipated problems involving risk to subjects or others, serious adverse events and all subject deaths associated with the protocol and provide an unbiased written report of the event. At a minimum, the medical monitor must comment on the outcomes of the event or problem and in case of a serious adverse event or death, comment on the relationship to participation in the study. The medical monitor must also indicate whether he/she concurs with the details of the report provided by the principal investigator. Reports for events determined by either the investigator or medical monitor to be possibly or definitely related to participation and reports of events resulting in death must be promptly forwarded to the USAMRMC ORP HRPO and the WRAIR HURC.

# 18 PUBLICATION POLICY

Following completion of the study, the results of this research will be published in a scientific journal. The trial will be registered in a public trials registry such as ClinicalTrials.gov, which is sponsored by the National Library of Medicine. It is the responsibility of DMID to register this trial in an acceptable registry.

# 19 LITERATURE


(1) Snow RW, Craig M, Deichmann U, Marsh K. Estimating mortality, morbidity and disability due to malaria among Africa's non-pregnant population. Bull World Health Organ **1999**;77:624-40.

(2) Alonso PL, Armstrong JR, Lindsay SW. Malaria, bednets, and mortality. Lancet **1991**;338:897.

(3) Nevill CG, Some ES, Mung'ala VO, et al. Insecticide-treated bednets reduce mortality and severe morbidity from malaria among children on the Kenyan coast. Trop Med Int Health **1996**;1:139-46.

(4) D'Alessandro U, Olaleye BO, McGuire W, et al. Mortality and morbidity from malaria in Gambian children after introduction of an impregnated bednet programme. Lancet **1995**;345:479-83.

(5) Coulibaly D, Diallo DA, Thera MA, et al. Impact of preseason treatment on incidence of falciparum malaria and parasite density at a site for testing malaria vaccines in Bandiagara, Mali. Am J Trop Med Hyg **2002**;67:604-10.

(6) Lyke KE, Dicko A, Kone A, et al. Incidence of severe Plasmodium falciparum malaria as a primary endpoint for vaccine efficacy trials in Bandiagara, Mali. Vaccine **2004**;22:3169-74.

(7) Cerami C, Frevert U, Sinnis P, et al. The basolateral domain of the hepatocyte plasma membrane bears receptors for the circumsporozoite protein of Plasmodium falciparum sporozoites. Cell **1992**;70:1021-33.

(8) Vreden SG, Verhave JP, Oettinger T, Sauerwein RW, Meuwissen JH. Phase I clinical trial of a recombinant malaria vaccine consisting of the circumsporozoite repeat region of Plasmodium falciparum coupled to hepatitis B surface antigen. Am J Trop Med Hyg **1991**;45:533-8.

(9) Alonso PL, Sacarlal J, Aponte JJ, et al. Efficacy of the RTS,S/AS02A vaccine against Plasmodium falciparum infection and disease in young African children: randomised controlled trial. Lancet **2004**;364:1411-20.

(10) Alonso PL, Sacarlal J, Aponte JJ, et al. Duration of protection with RTS,S/AS02A malaria vaccine in prevention of Plasmodium falciparum disease in Mozambican children: single-blind extended follow-up of a randomised controlled trial. Lancet **2005**;366:2012-8.

(11) Heppner DG, Jr., Kester KE, Ockenhouse CF, et al. Towards an RTS,S-based, multi-stage, multi-antigen vaccine against falciparum malaria: progress at the Walter Reed Army Institute of Research. Vaccine **2005**;23:2243-50.

(12) Narum DL, Thomas AW. Differential localization of full-length and processed forms of PF83/AMA-1 an apical membrane antigen of Plasmodium falciparum merozoites. Mol Biochem Parasitol **1994**;67:59-68.

(13) Crewther PE, Culvenor JG, Silva A, Cooper JA, Anders RF. Plasmodium falciparum: two antigens of similar size are located in different compartments of the rhoptry. Exp Parasitol **1990**;70:193-206.

(14) Waters AP, Thomas AW, Deans JA, et al. A merozoite receptor protein from Plasmodium knowlesi is highly conserved and distributed throughout Plasmodium. J Biol Chem **1990**;265:17974-9.

(15) Kocken CH, van der Wel AM, Dubbeld MA, et al. Precise timing of expression of a Plasmodium falciparum-derived transgene in Plasmodium berghei is a critical determinant of subsequent subcellular localization. J Biol Chem **1998**;273:15119-24.

(16) Deans JA, Alderson T, Thomas AW, Mitchell GH, Lennox ES, Cohen S. Rat monoclonal antibodies which inhibit the in vitro multiplication of Plasmodium knowlesi. Clin Exp Immunol **1982**;49:297-309.

(17) Thomas AW, Deans JA, Mitchell GH, Alderson T, Cohen S. The Fab fragments of monoclonal IgG to a merozoite surface antigen inhibit Plasmodium knowlesi invasion of erythrocytes. Mol Biochem Parasitol **1984**;13:187-99.

(18) Peterson MG, Marshall VM, Smythe JA, et al. Integral membrane protein located in the apical complex of Plasmodium falciparum. Mol Cell Biol **1989**;9:3151-4.

(19) Waters AP, Thomas AW, Deans JA, et al. A merozoite receptor protein from Plasmodium knowlesi is highly conserved and distributed throughout Plasmodium. J Biol Chem **1990**;265:17974-9.

(20) Marshall VM, Zhang L, Anders RF, Coppel RL. Diversity of the vaccine candidate AMA-1 of Plasmodium falciparum. Mol Biochem Parasitol **1996**;77:109-13.

(21) Oliveira DA, Udhayakumar V, Bloland P, et al. Genetic conservation of the Plasmodium falciparum apical membrane antigen-1 (AMA-1). Mol Biochem Parasitol **1996**;76:333-6.

(22) Hodder AN, Crewther PE, Matthew ML, et al. The disulfide bond structure of Plasmodium apical membrane antigen-1. J Biol Chem **1996**;271:29446-52.

(23) Thomas AW, Trape JF, Rogier C, Goncalves A, Rosario VE, Narum DL. High prevalence of natural antibodies against Plasmodium falciparum 83-kilodalton apical membrane antigen (PF83/AMA-1) as detected by capture-enzyme-linked immunosorbent assay using full-length baculovirus recombinant PF83/AMA-1. Am J Trop Med Hyg **1994**;51:730-40.

(24) Udhayakumar V, Kariuki S, Kolczack M, et al. Longitudinal study of natural immune responses to the Plasmodium falciparum apical membrane antigen (AMA-1) in a holoendemic region of malaria in western Kenya: Asembo Bay Cohort Project VIII. Am J Trop Med Hyg **2001**;65:100-7.

(25) Dolo A, Thera MA, Baby M. Strain-specific antibody responses to potential malaria vaccine antigens in Mali. 69 ed. **2003**. p. 495.

(26) Hodder AN, Crewther PE, Anders RF. Specificity of the protective antibody response to apical membrane antigen 1. Infect Immun **2001**;69:3286-94.

(27) Cortes A, Mellombo M, Mueller I, Benet A, Reeder JC, Anders RF. Geographical structure of diversity and differences between symptomatic and asymptomatic infections for Plasmodium falciparum vaccine candidate AMA1. Infect Immun **2003**;71:1416-26.

(28) Kennedy MC, Wang J, Zhang Y, et al. In vitro studies with recombinant Plasmodium falciparum apical membrane antigen 1 (AMA1): production and activity of an AMA1 vaccine and generation of a multiallelic response. Infect Immun **2002**;70:6948-60.

(29) Kocken CH, Withers-Martinez C, Dubbeld MA, et al. High-level expression of the malaria blood-stage vaccine candidate Plasmodium falciparum apical membrane antigen 1 and induction of antibodies that inhibit erythrocyte invasion. Infect Immun **2002**;70:4471-6.

(30) Healer J, Murphy V, Hodder AN, et al. Allelic polymorphisms in apical membrane antigen-1 are responsible for evasion of antibody-mediated inhibition in Plasmodium falciparum. Mol Microbiol **2004**;52:159-68.

(31) Plowe CV, Thera MA, Takala SL, et al. Dynamics of *Plasmodium falciparum* apical membrant antigen-1 sequence variation over three years at a malaria vaccine testing site in Bandiagara, Mali. 73 ed. **2005**. p. 277.

(32) Gordon DM, McGovern TW, Krzych U, et al. Safety, immunogenicity, and efficacy of a recombinantly produced Plasmodium falciparum circumsporozoite protein-hepatitis B surface antigen subunit vaccine. J Infect Dis **1995**;171:1576-85.

(33) Wu JY, Gardner BH, Murphy CI, et al. Saponin adjuvant enhancement of antigen-specific immune responses to an experimental HIV-1 vaccine. J Immunol **1992**;148:1519-25.

(34) Soltysik S, Bedore DA, Kensil CR. Adjuvant activity of QS-21 isomers. Ann N Y Acad Sci **1993**;690:392-5.

(35) Ribi E, Cantrell J, Feldner T. Microbiology. Washington, DC: American Society of Microbiology, **1986**.

(36) Myers KR, Truchot AT, Ward J. Cellular and molecular aspects of endotoxin reactions. In: Nowotny A, ed.Amsterdam: **1990**. p. 145-56.

(37) Loppnow H, Durrbaum I, Brade H, et al. Lipid A, the immunostimulatory principle of lipopolysaccharides? Adv Exp Med Biol **1990**;256:561-6.

(38) Johnson AG, Tomai MA. A study of the cellular and molecular mediators of the adjuvant action of a nontoxic monophosphoryl lipid A. Adv Exp Med Biol **1990**;256:567-79.

(39) Doherty JF, Pinder M, Tornieporth N, et al. A phase I safety and immunogenicity trial with the candidate malaria vaccine RTS,S/SBAS2 in semi-immune adults in The Gambia. Am J Trop Med Hyg **1999**;61:865-8.

(40) Bojang KA, Milligan PJ, Pinder M, et al. Efficacy of RTS,S/AS02 malaria vaccine against Plasmodium falciparum infection in semi-immune adult men in The Gambia: a randomised trial. Lancet **2001**;358:1927-34.

(41) Bojang KA, Olodude F, Pinder M, et al. Safety and immunogenicty of RTS,S/AS02A candidate malaria vaccine in Gambian children. Vaccine **2005**;23:4148-57.

(42) Stoute JA, Gombe J, Withers MR, et al. Phase 1 randomized double-blind safety and immunogenicity trial of Plasmodium falciparum malaria merozoite surface protein FMP1 vaccine, adjuvanted with AS02A, in adults in western Kenya. Vaccine **2005**.

(43) Withers MR, McKinney D, Ogutu BR, et al. Safety and Reactogenicity of an MSP-1 Malaria Vaccine Candidate: A Randomized Phase Ib Dose-Escalation Trial in Kenyan Children. PLoS Clin Trials **2006**;1:e32.

(44) Dreesen DW, Fishbein DB, Kemp DT, Brown J. Two-year comparative trial on the immunogenicity and adverse effects of purified chick embryo cell rabies vaccine for pre-exposure immunization. Vaccine **1989**;7:397-400.

(45) Nicholson KG, Farrow PR, Bijok U, Barth R. Pre-exposure studies with purified chick embryo cell culture rabies vaccine and human diploid cell vaccine: serological and clinical responses in man. Vaccine **1987**;5:208-10.

(46) Vodopija I, Sureau P, Lafon M, et al. An evaluation of second generation tissue culture rabies vaccines for use in man: a four-vaccine comparative immunogenicity study using a pre-exposure vaccination schedule and an abbreviated 2-1-1 postexposure schedule. Vaccine **1986**;4:245-8.

(47) Wasi C, Chaiprasithikul P, Chavanich L, Puthavathana P, Thongcharoen P, Trishanananda M. Purified chick embryo cell rabies vaccine. Lancet **1986**;1:40.

(48) Cox JH, Schneider LG. Prophylactic immunization of humans against rabies by intradermal inoculation of human diploid cell culture vaccine. J Clin Microbiol **1976**;3:96-101.

(49) Kuwert EK, Marcus I, Werner J, Iwand A, Thraenhart O. Some experiences with human diploid cell strain-(HDCS) rabies vaccine in pre- and post-exposure vaccinated humans. Dev Biol Stand **1978**;40:79-88.

(50) Ajjan N, Soulebot JP, Stellmann C, et al. [Results of preventive rabies vaccination with a concentrated vaccine of the PM/WI38-1503-3M rabies strain cultured on human diploid cells. Preparation of mixed antirabies-antitetanus hyperimmune immunoglobulin by plasmapheresis of blood taken from vaccinated veterinary students]. Dev Biol Stand **1978**;40:89-100.

(51) Costy-Berger F. [Preventive rabies vaccination using vaccine prepared from human diploid cells]. Dev Biol Stand **1978**;40:101-4.

(52) Lyke KE, Diallo DA, Dicko A, et al. Association of intraleukocytic Plasmodium falciparum malaria pigment with disease severity, clinical manifestations, and prognosis in severe malaria. Am J Trop Med Hyg **2003**;69:253-9.

(53) Lyke KE, Burges R, Cissoko Y, et al. Serum Levels of the Proinflammatory Cytokines Interleukin-1 Beta (IL-1{beta}), IL-6, IL-8, IL-10, Tumor Necrosis Factor Alpha, and IL-12(p70) in Malian Children with Severe Plasmodium falciparum Malaria and Matched Uncomplicated Malaria or Healthy Controls. Infect Immun **2004**;72:5630-7.

(54) Djimde A, Doumbo OK, Cortese JF, et al. A molecular marker for chloroquine-resistant falciparum malaria. N Engl J Med **2001**;344:257-63.

(55) Smith T, Schellenberg JA, Hayes R. Attributable fraction estimates and case definitions for malaria in endemic areas. Statistics in Medicine **1994**;13:2345-58.

(56) Severe falciparum malaria. World Health Organization, Communicable Diseases Cluster [In Process Citation]. Trans R Soc Trop Med Hyg **2000**;94 Suppl 1:S1-90.

(57) Djimde AA, Doumbo OK, Traore O, et al. Clearance of drug-resistant parasites as a model for protective immunity in Plasmodium falciparum malaria. Am J Trop Med Hyg **2003**;69:558-63.

(58) Nei M. Molecular Evolutionary Genetics. Columbia University Press, **1987**.

(59) Kumar S, Tamura K, Nei M. MEGA3: Integrated software for Molecular Evolutionary Genetics Analysis and
sequence alignment. Brief Bioinform **2004**;5:150-63.

(60) Diallo DA, Doumbo OK, Plowe CV, Wellems TE, Emanuel EJ, Hurst SA. Community permission for medical research in developing countries. Clin Infect Dis **2005**;41:255-9.

# 20 SUPPLEMENTS/APPENDICES

Consent forms are attached as separate files.
